# Supplementary material for: A multi-level gene-diet interaction analysis of fish oil and 14 polyunsaturated fatty acid traits identifies the FADS and GPR12 loci
Source: HGG Adv. 2025 May 21;6(3):100459. doi: 10.1016/j.xhgg.2025.100459 (PMC12172259; doi:10.1016/j.xhgg.2025.100459)
Supplement: Document S1. Figures S1–S5 and Table S1 [file mmc1.pdf]

HGGA, Volume *6*

Supplemental information

A multi-level gene-diet interaction analysis of fish oil and 14 polyunsaturated fatty acid traits identifies the *FADS* and *GPR12* loci

Susan Adanna Ihejirika, Alexandra Huong Chiang, Aryaman Singh, Eunice Stephen, Han Chen, and Kaixiong Ye

Table of Contents

Figure S1. Participant flowchart.......... 2

Figure S2. Manhattan and QQ plots of p-values for gene-FOS interactions in 14 PUFAs and MUFAs-related phenotypes for 85,708 participants in the Phase One dataset.......... 9

Figure S3. Scatter plots of p-values, across the genome, of gene-FOS interactions across 14 PUFAs and MUFAs-related phenotypes in the Phases One and Two datasets.......... 13

Figure S4. Manhattan and QQ plots of p-values for gene-fish oil interactions in 14 PUFAs and MUFAs-related phenotypes for 114,352 participants in the Phase Two dataset.......... 20

Figure S5. Manhattan and QQ plots of p-values for gene-fish oil interactions in 14 PUFAs and MUFAs-related phenotypes for 200,060 participants in the combined dataset.......... 27

Table S1. Baseline characteristics of participants in Phase One, Phase Two, and combined releases of NMR metabolite data of individuals of European ancestry in UK Biobank.......... 28

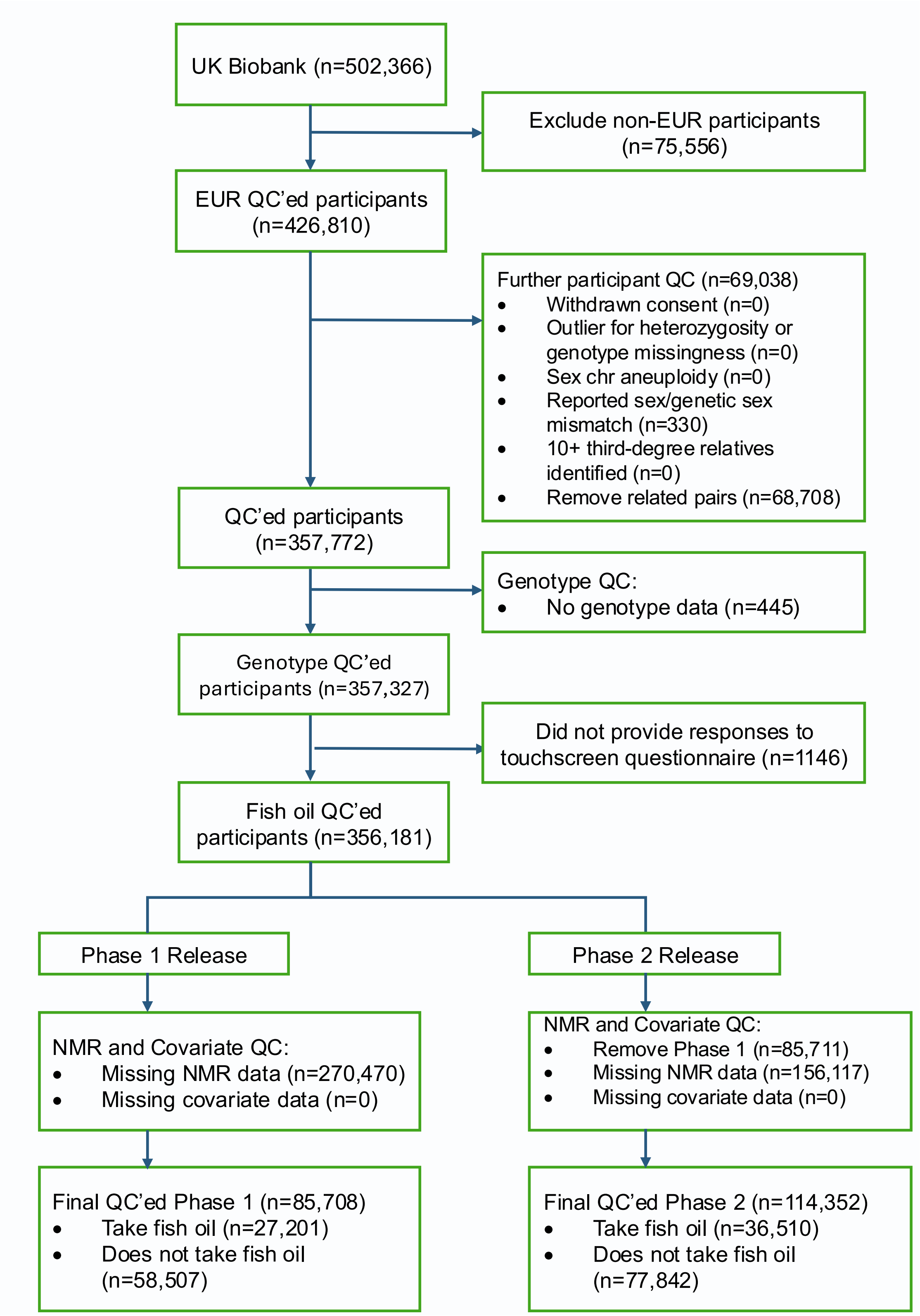

Figure S1. Participant flowchart

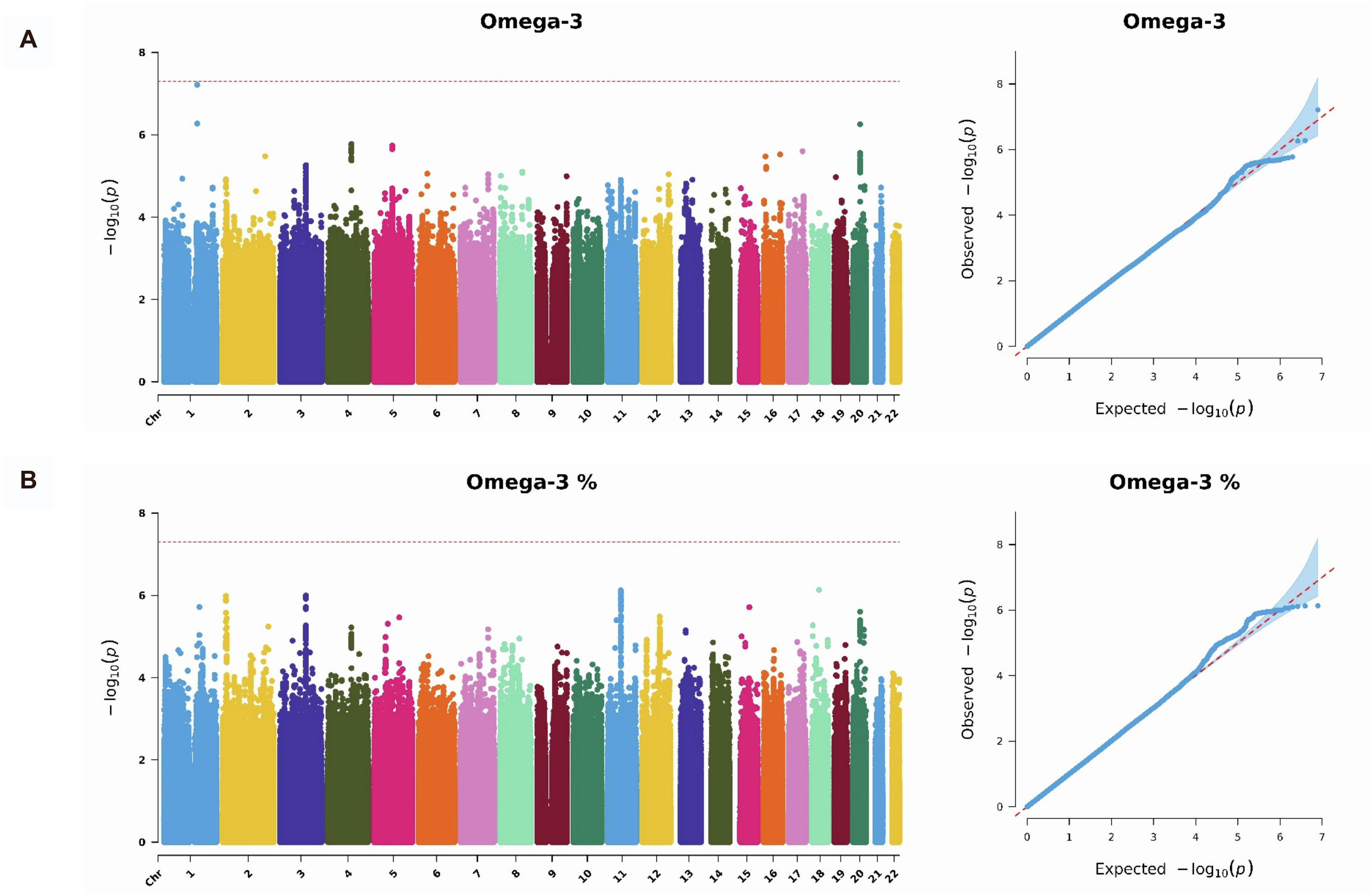
A
Omega-3
Omega-3
$-\log_{10}(p)$
Observed $-\log_{10}(p)$
Expected $-\log_{10}(p)$
Chr
B
Omega-3 %
Omega-3 %
$-\log_{10}(p)$
Observed $-\log_{10}(p)$
Expected $-\log_{10}(p)$
Chr

C

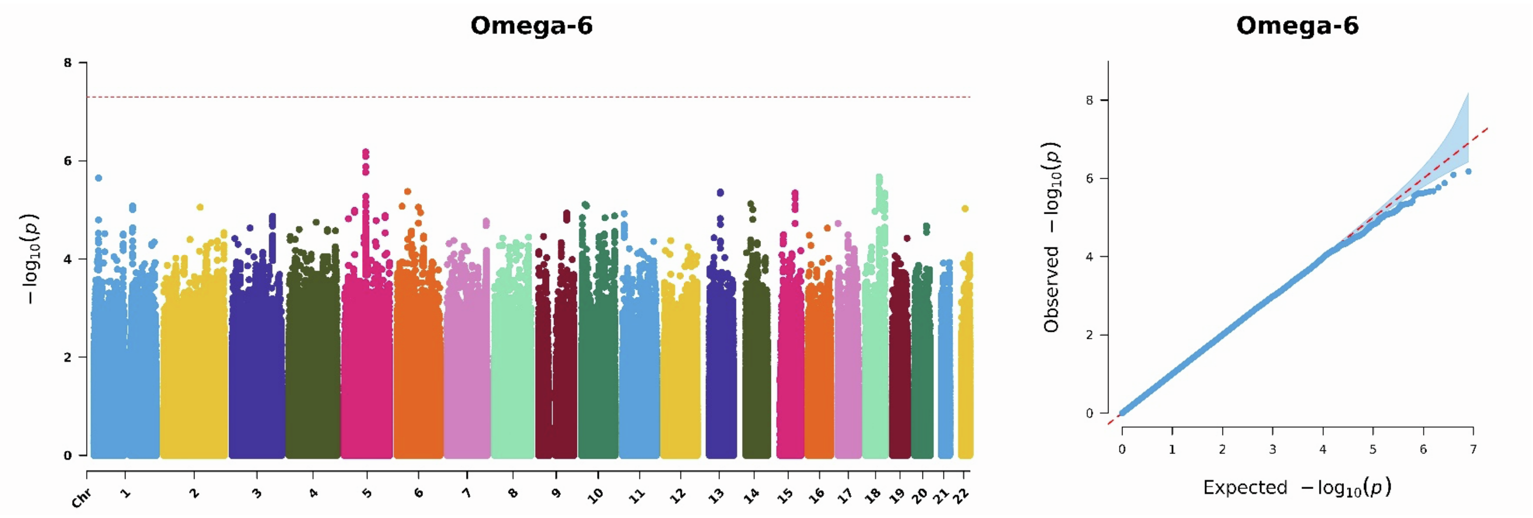
Omega-6
Omega-6
$-\log_{10}(p)$
Chr
1 2 3 4 5 6 7 8 9 10 11 12 13 14 15 16 17 18 19 20 21 22
Observed $-\log_{10}(p)$
Expected $-\log_{10}(p)$

D

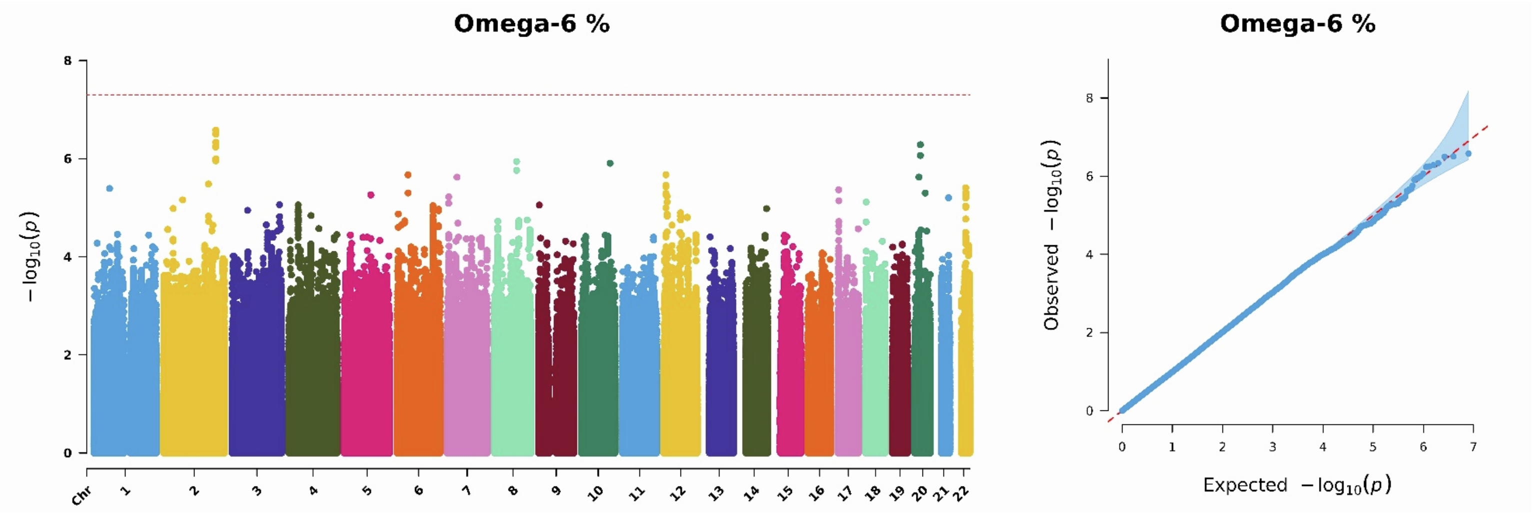
Omega-6 %
Omega-6 %
$-\log_{10}(p)$
Chr
1 2 3 4 5 6 7 8 9 10 11 12 13 14 15 16 17 18 19 20 21 22
Observed $-\log_{10}(p)$
Expected $-\log_{10}(p)$

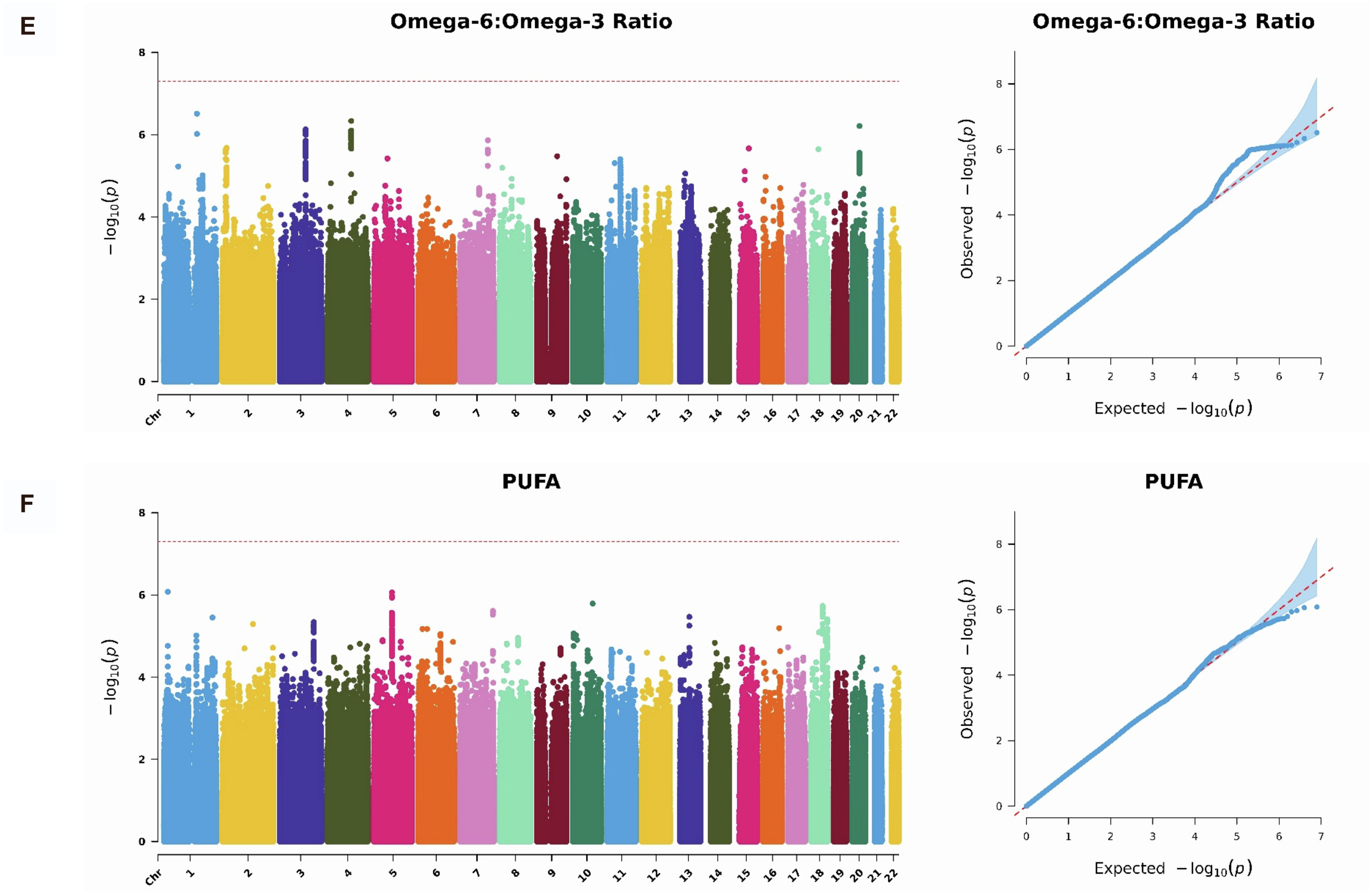
E
Omega-6:Omega-3 Ratio
−log10(p)
Chr 1 2 3 4 5 6 7 8 9 10 11 12 13 14 15 16 17 18 19 20 21 22
Omega-6:Omega-3 Ratio
Observed −log10(p)
Expected −log10(p)
F
PUFA
−log10(p)
Chr 1 2 3 4 5 6 7 8 9 10 11 12 13 14 15 16 17 18 19 20 21 22
PUFA
Observed −log10(p)
Expected −log10(p)

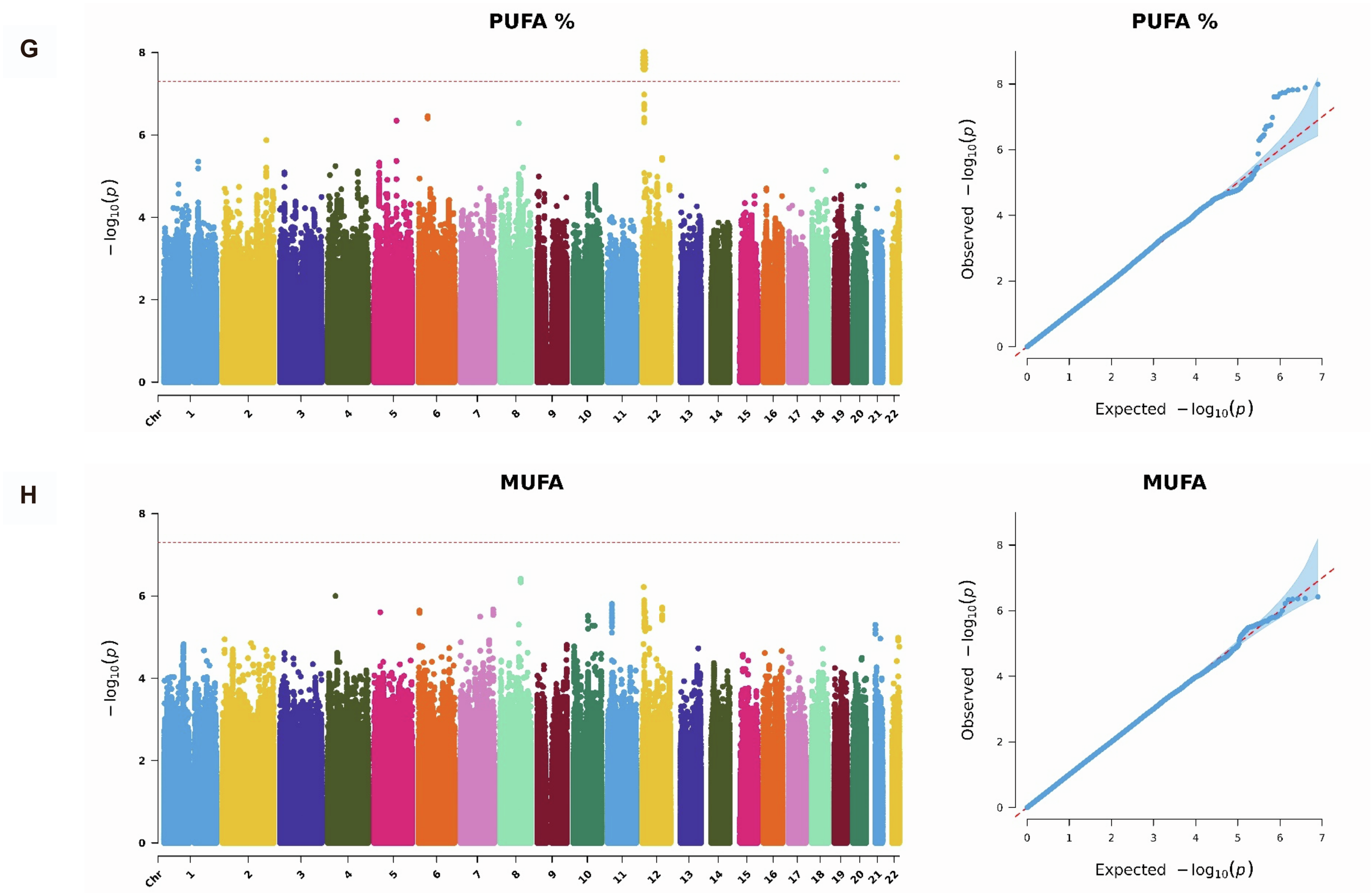
G
PUFA %
$-\log_{10}(p)$
Chr
1
2
3
4
5
6
7
8
9
10
11
12
13
14
15
16
17
18
19
20
21
22
PUFA %
Observed $-\log_{10}(p)$
Expected $-\log_{10}(p)$
H
MUFA
$-\log_{10}(p)$
Chr
MUFA
Observed $-\log_{10}(p)$
Expected $-\log_{10}(p)$

I

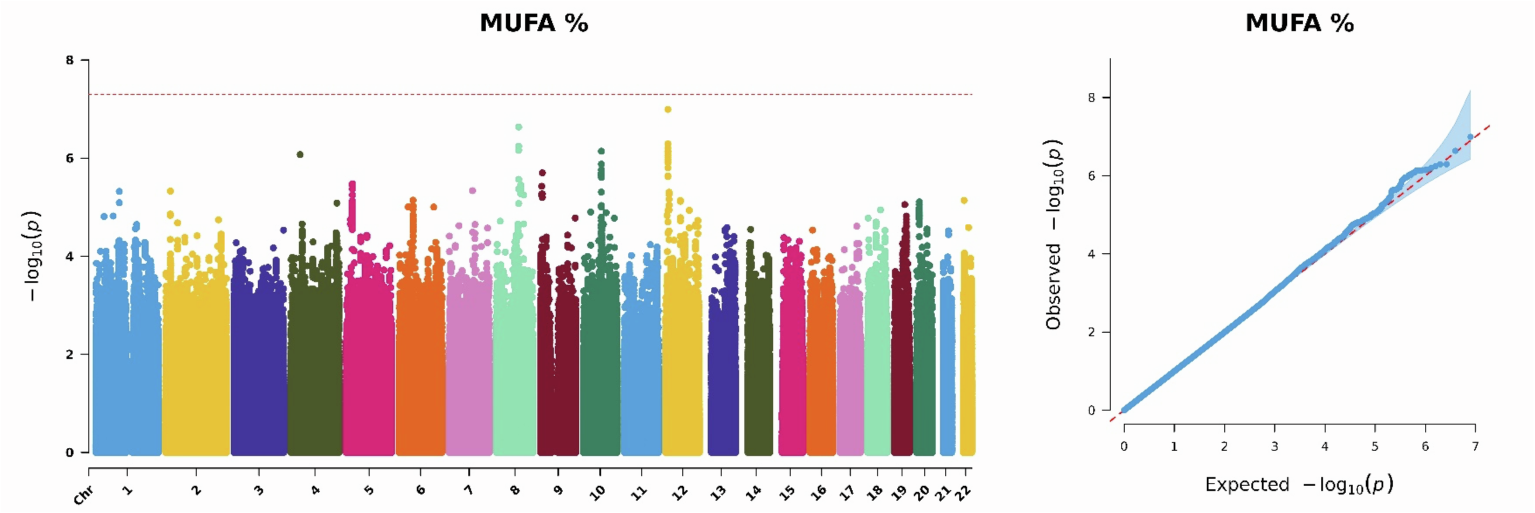
MUFA %
MUFA %
$-\log_{10}(p)$
Observed $-\log_{10}(p)$
Expected $-\log_{10}(p)$
Chr

J

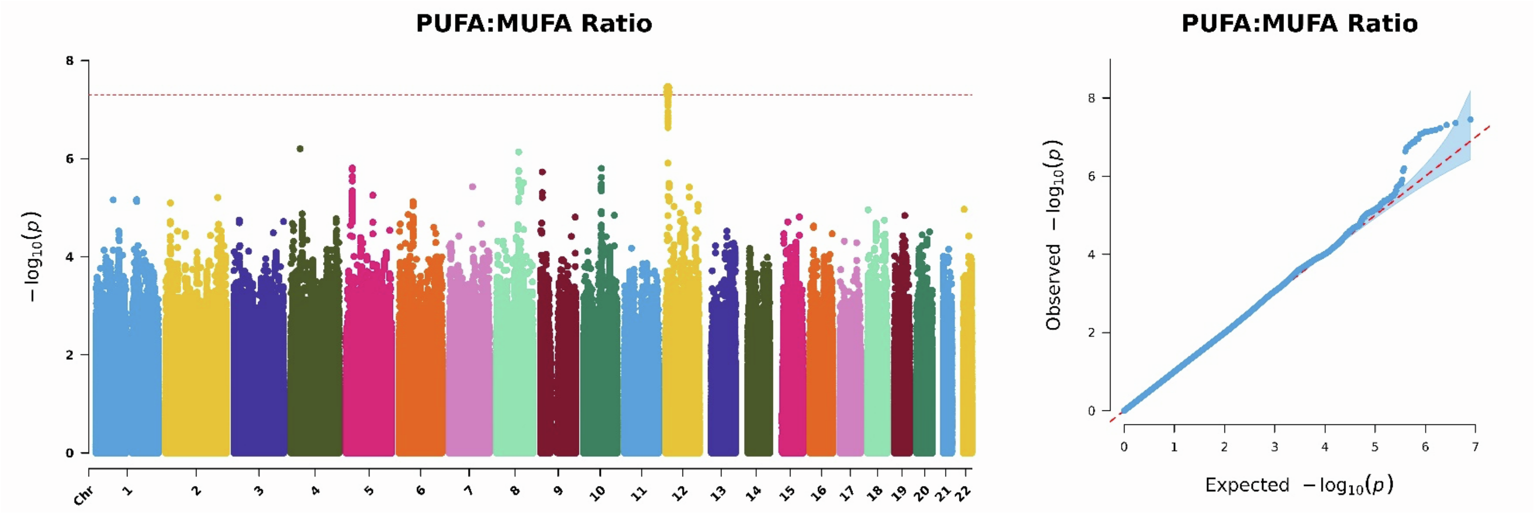
PUFA:MUFA Ratio
PUFA:MUFA Ratio
$-\log_{10}(p)$
Observed $-\log_{10}(p)$
Expected $-\log_{10}(p)$
Chr

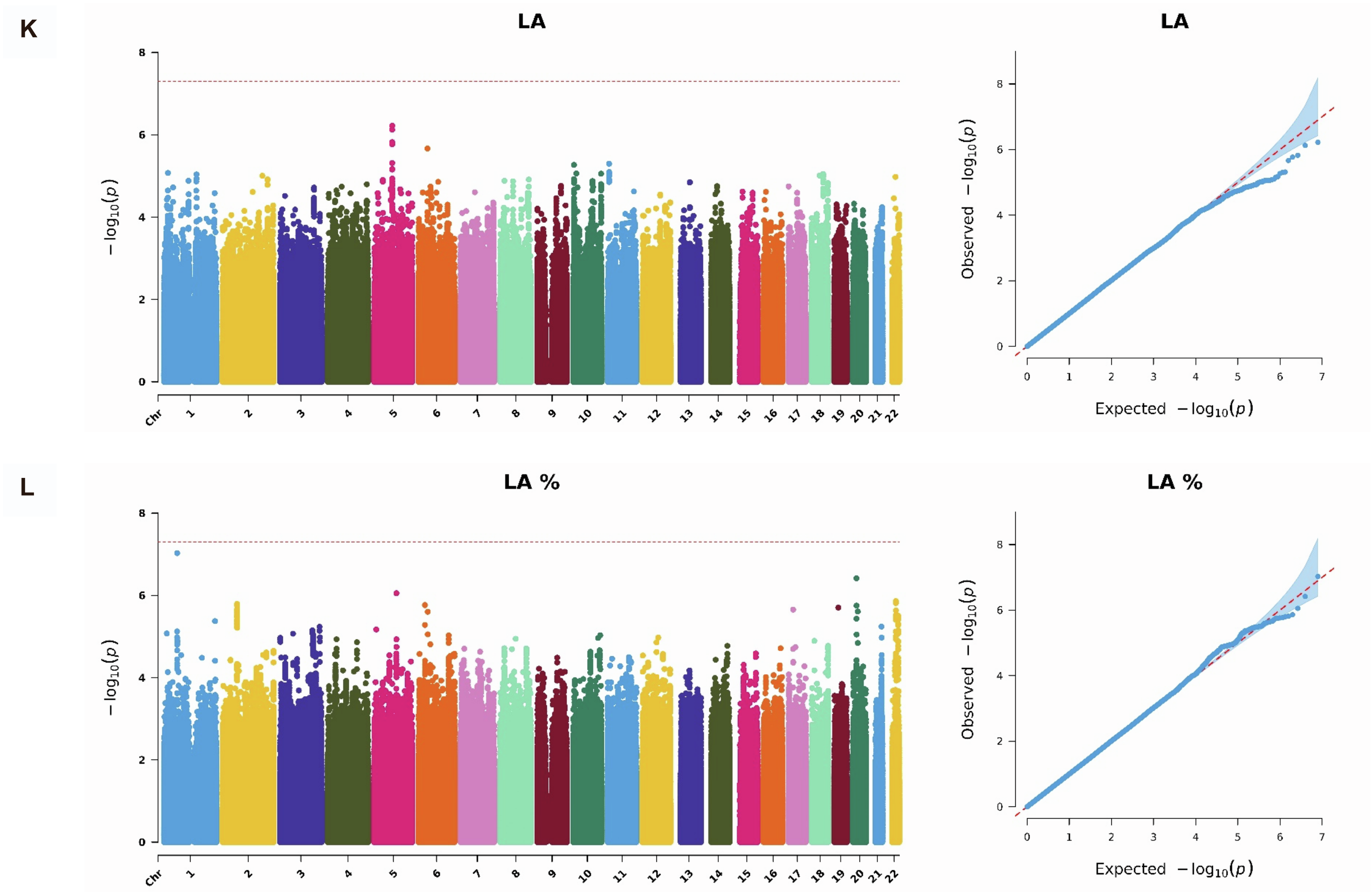
K
LA
LA
$-\log_{10}(p)$
Chr
Observed $-\log_{10}(p)$
Expected $-\log_{10}(p)$
L
LA %
LA %
$-\log_{10}(p)$
Chr
Observed $-\log_{10}(p)$
Expected $-\log_{10}(p)$

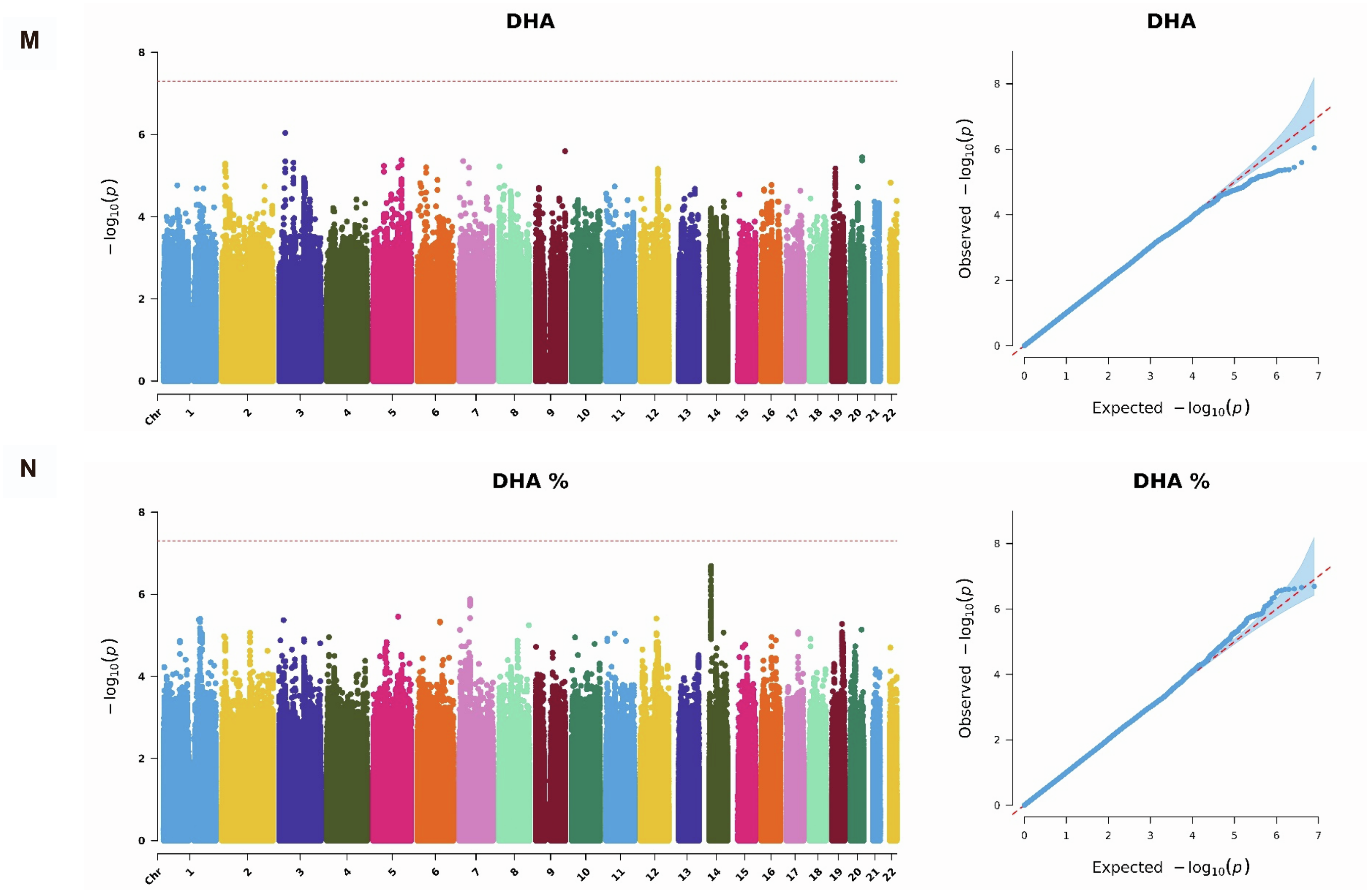

Figure S2. Manhattan and QQ plots of *p*-values for gene-FOS interactions in 14 PUFAs and MUFAs-related phenotypes for 85,708 participants in the Phase One dataset

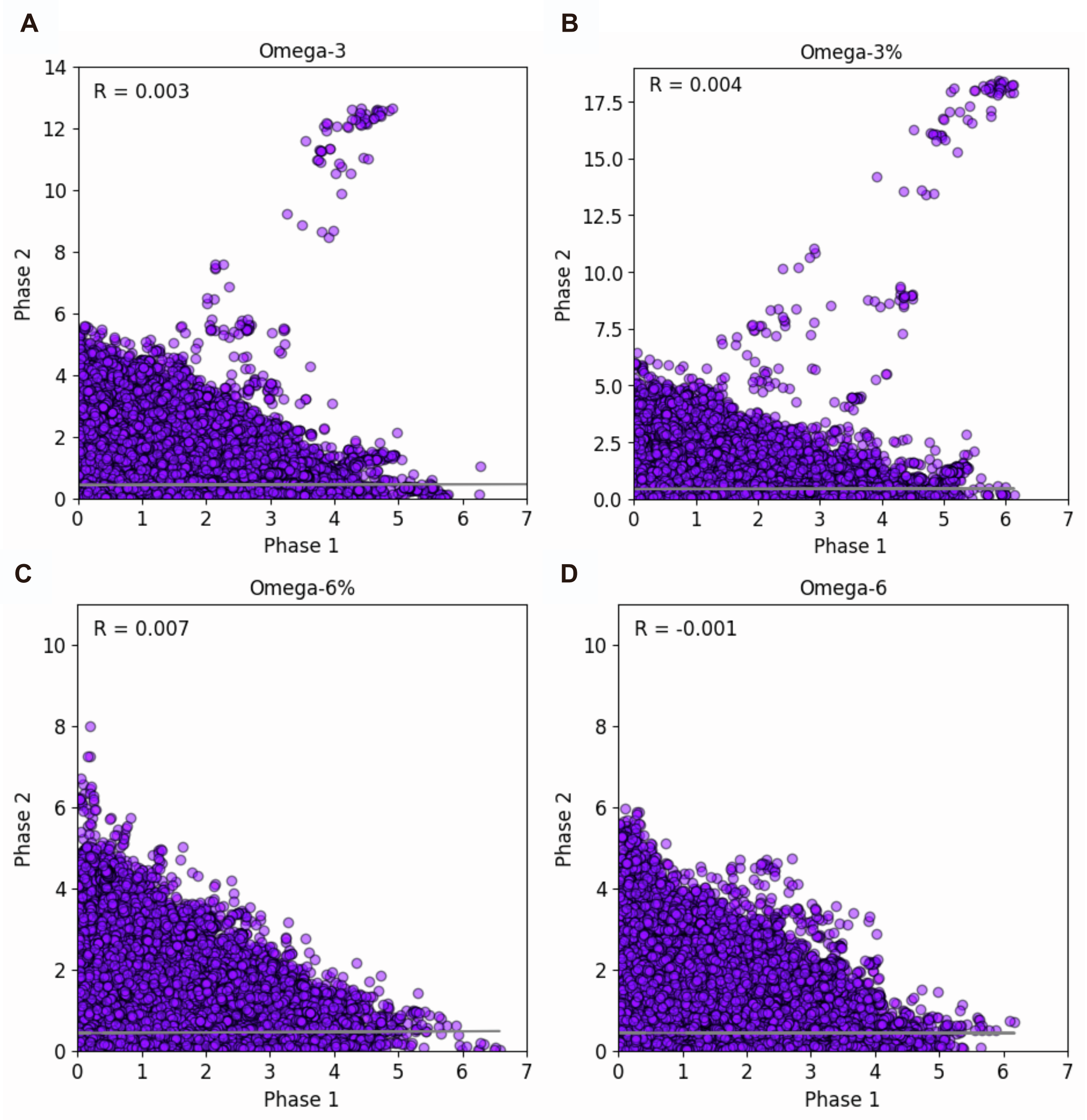

A
Omega-3
R = 0.003
Phase 2
Phase 1
B
Omega-3%
R = 0.004
Phase 2
Phase 1
C
Omega-6%
R = 0.007
Phase 2
Phase 1
D
Omega-6
R = -0.001
Phase 2
Phase 1

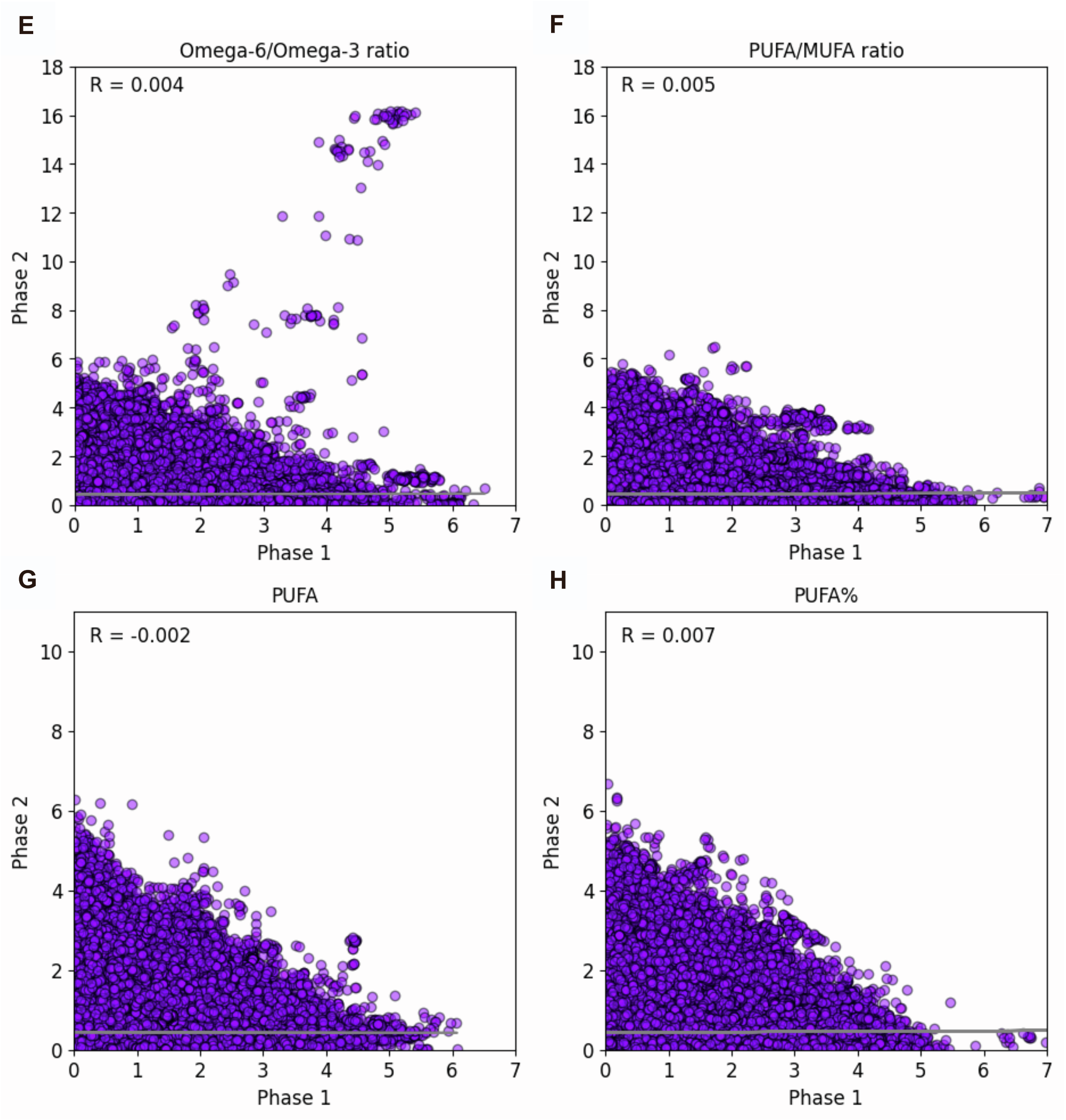

E
Omega-6/Omega-3 ratio
R = 0.004
Phase 2
Phase 1
F
PUFA/MUFA ratio
R = 0.005
Phase 2
Phase 1
G
PUFA
R = -0.002
Phase 2
Phase 1
H
PUFA%
R = 0.007
Phase 2
Phase 1

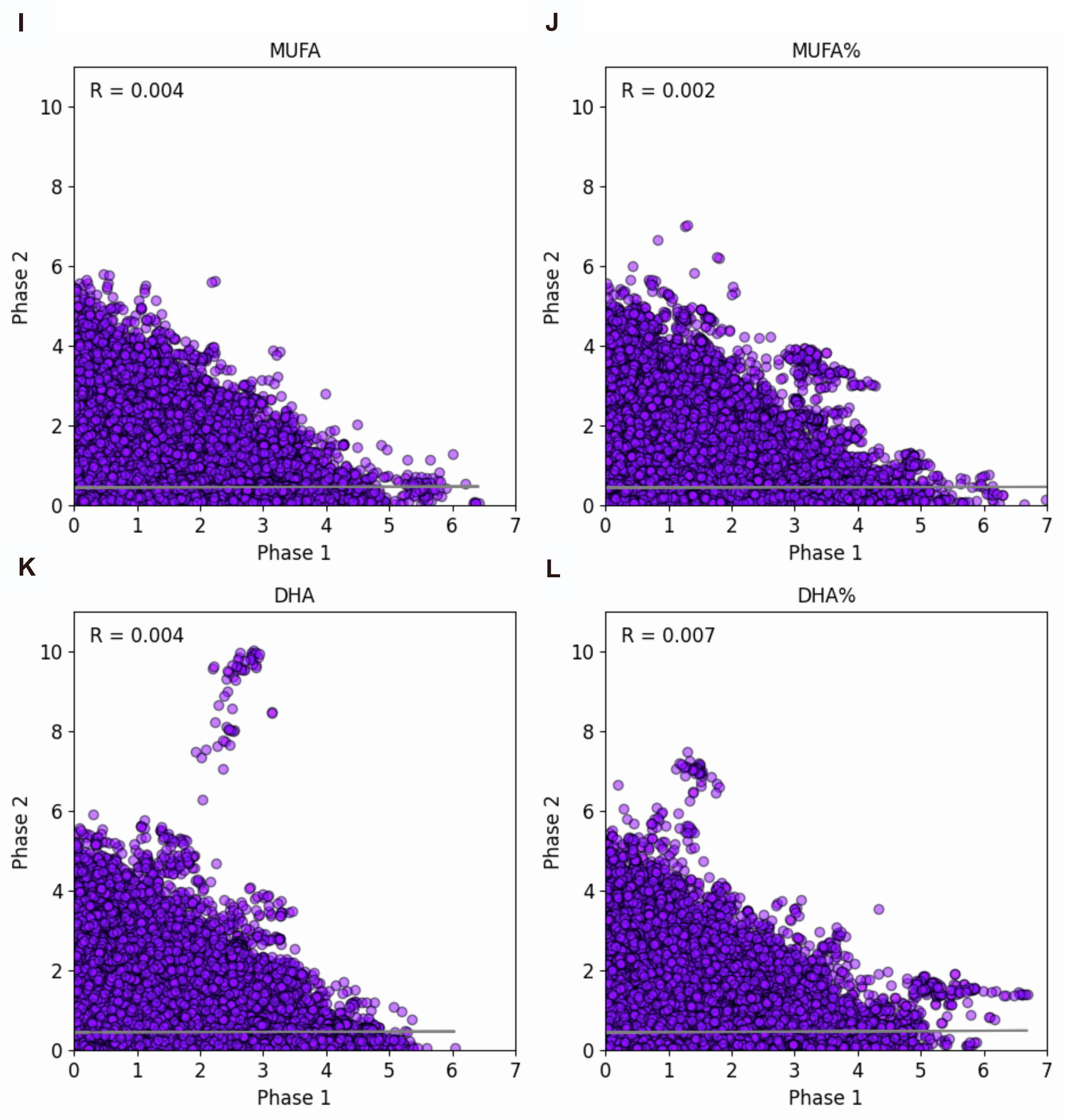

I
MUFA
R = 0.004
J
MUFA%
R = 0.002
K
DHA
R = 0.004
L
DHA%
R = 0.007
Phase 1
Phase 2

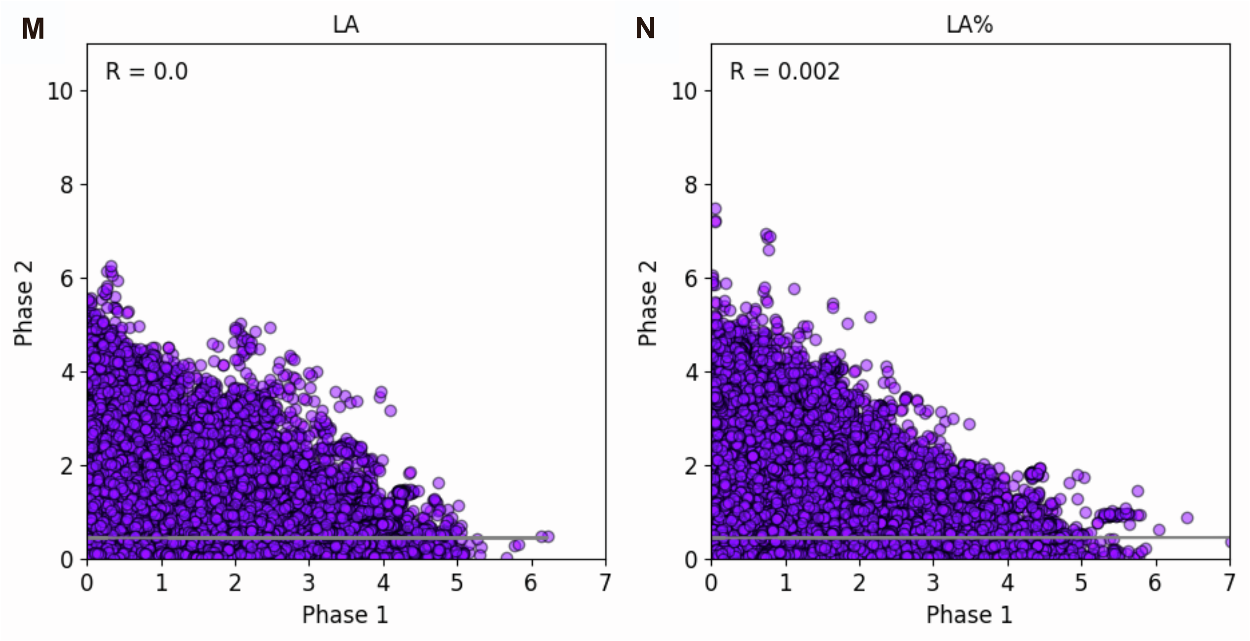

Figure S3. Scatter plots of *p*-values, across the genome, of gene-FOS interactions across 14 PUFAs and MUFAs-related phenotypes in the Phases One and Two datasets

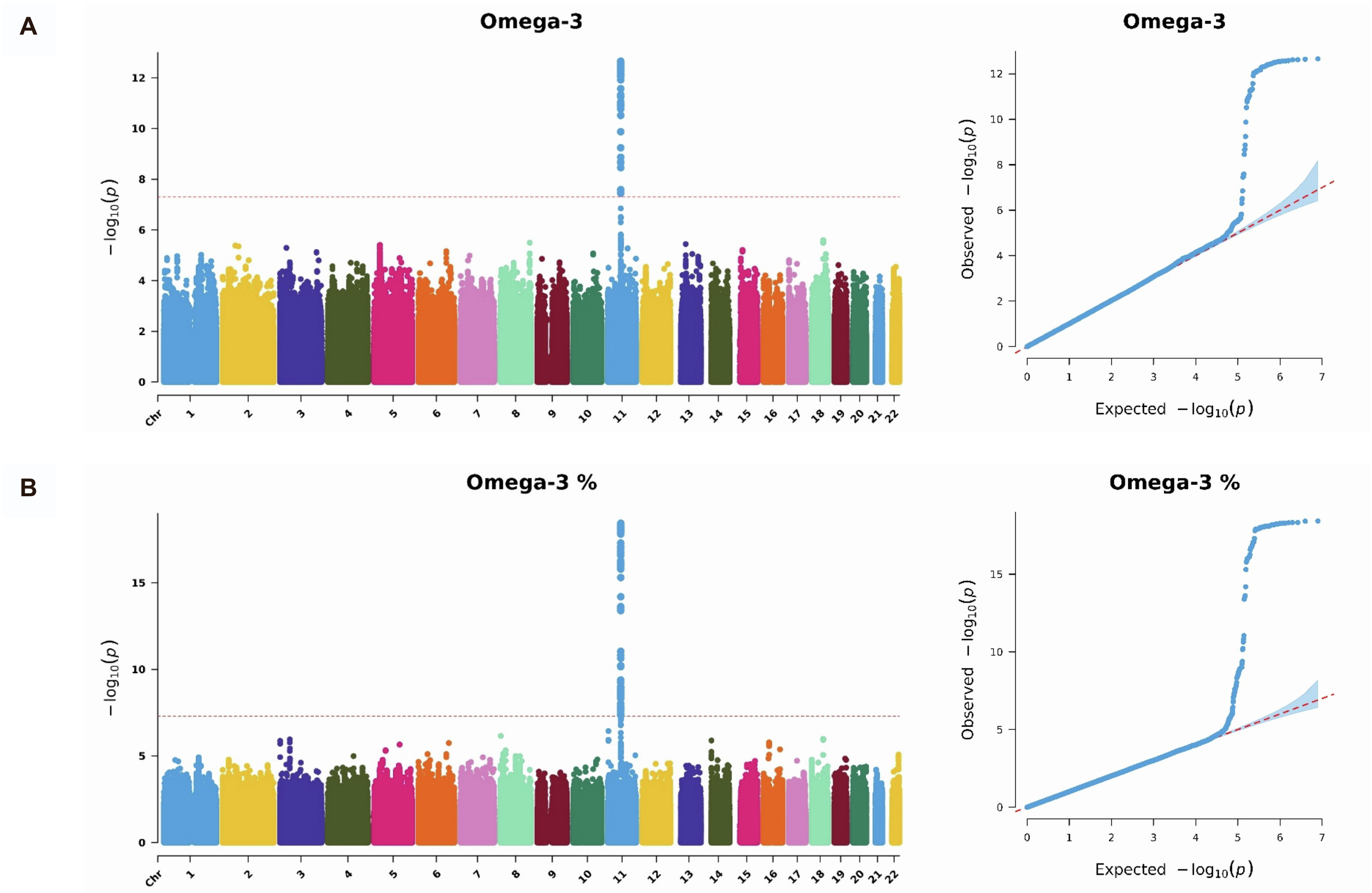
A
Omega-3
−log10(p)
Chr 1 2 3 4 5 6 7 8 9 10 11 12 13 14 15 16 17 18 19 20 21 22
Omega-3
Observed −log10(p)
Expected −log10(p)
B
Omega-3 %
−log10(p)
Chr 1 2 3 4 5 6 7 8 9 10 11 12 13 14 15 16 17 18 19 20 21 22
Omega-3 %
Observed −log10(p)
Expected −log10(p)

C

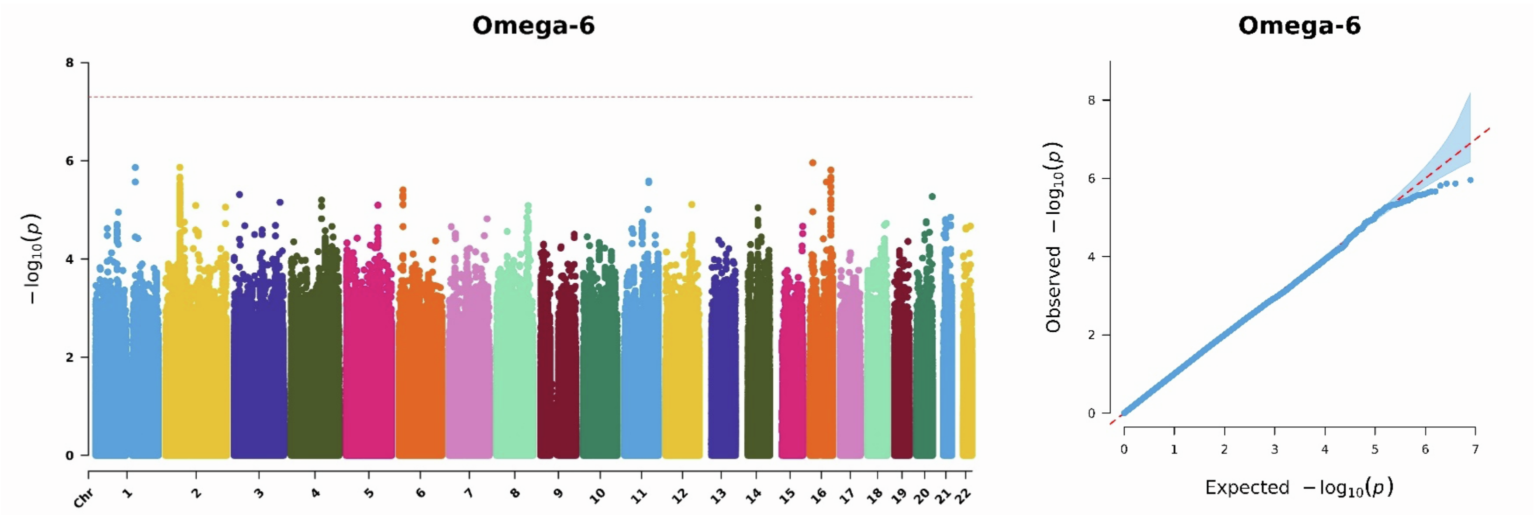
Omega-6
Omega-6
8
6
4
2
0
$-\log_{10}(p)$
Chr
1
2
3
4
5
6
7
8
9
10
11
12
13
14
15
16
17
18
19
20
21
22
Observed $-\log_{10}(p)$
Expected $-\log_{10}(p)$
0
1
2
3
4
5
6
7

D

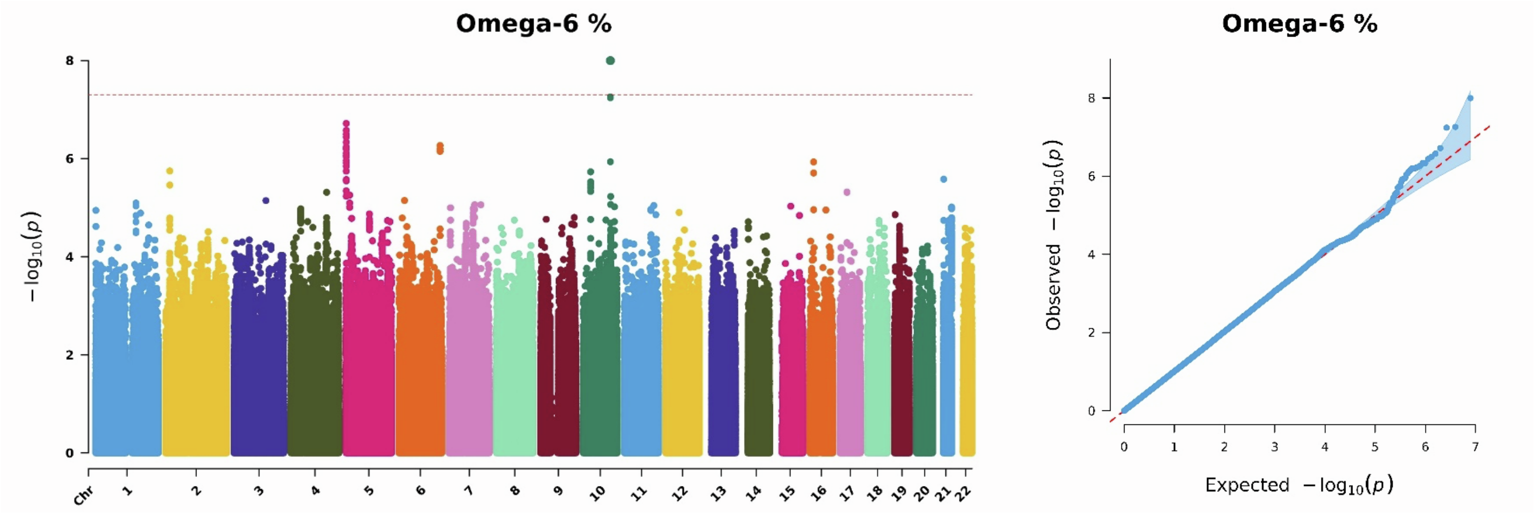
Omega-6 %
Omega-6 %
8
6
4
2
0
$-\log_{10}(p)$
Chr
1
2
3
4
5
6
7
8
9
10
11
12
13
14
15
16
17
18
19
20
21
22
Observed $-\log_{10}(p)$
Expected $-\log_{10}(p)$
0
1
2
3
4
5
6
7

E

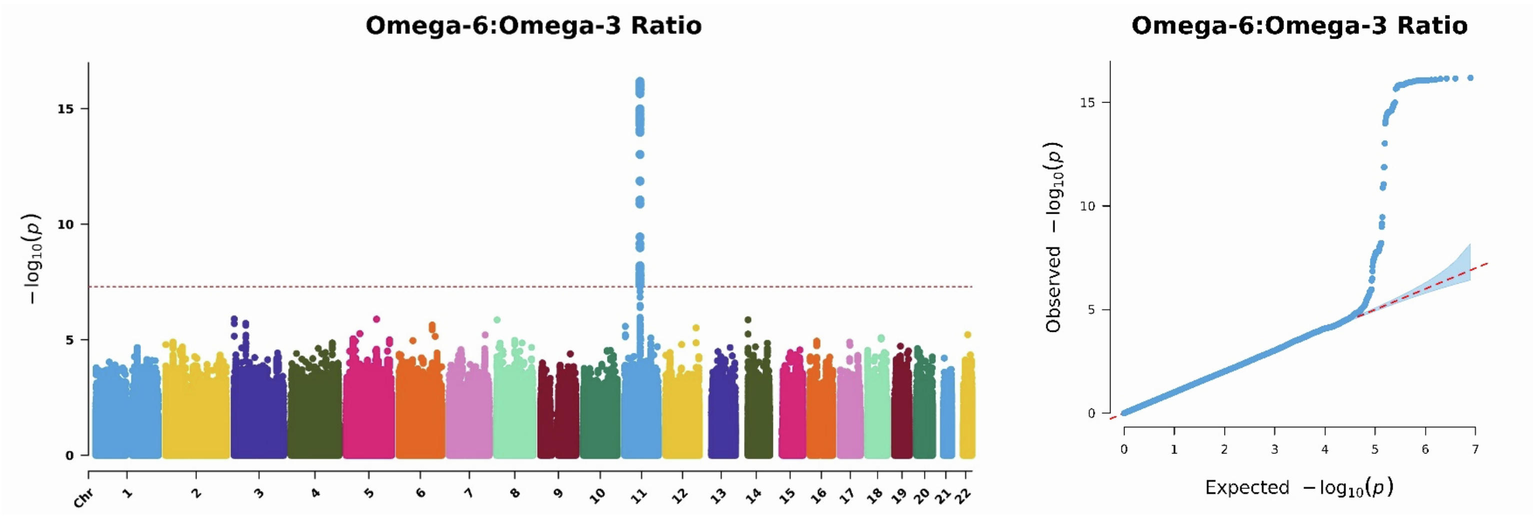

Omega-6:Omega-3 Ratio
$-\log_{10}(p)$
Chr
Omega-6:Omega-3 Ratio
Observed $-\log_{10}(p)$
Expected $-\log_{10}(p)$

F

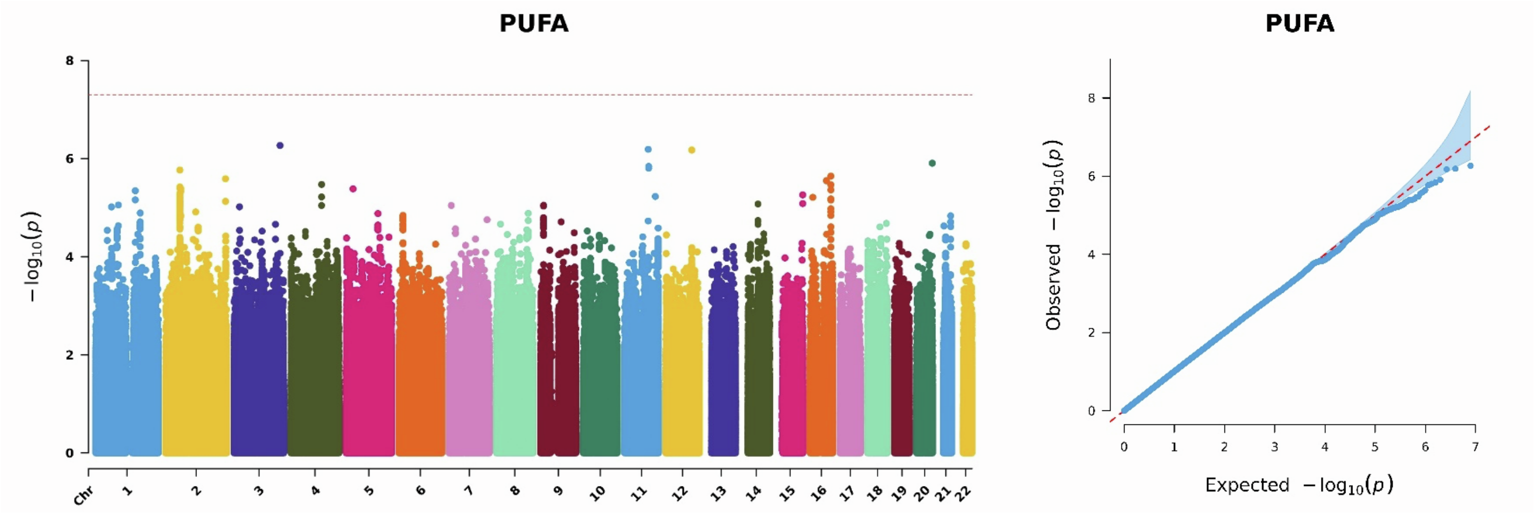

PUFA
$-\log_{10}(p)$
Chr
PUFA
Observed $-\log_{10}(p)$
Expected $-\log_{10}(p)$

G

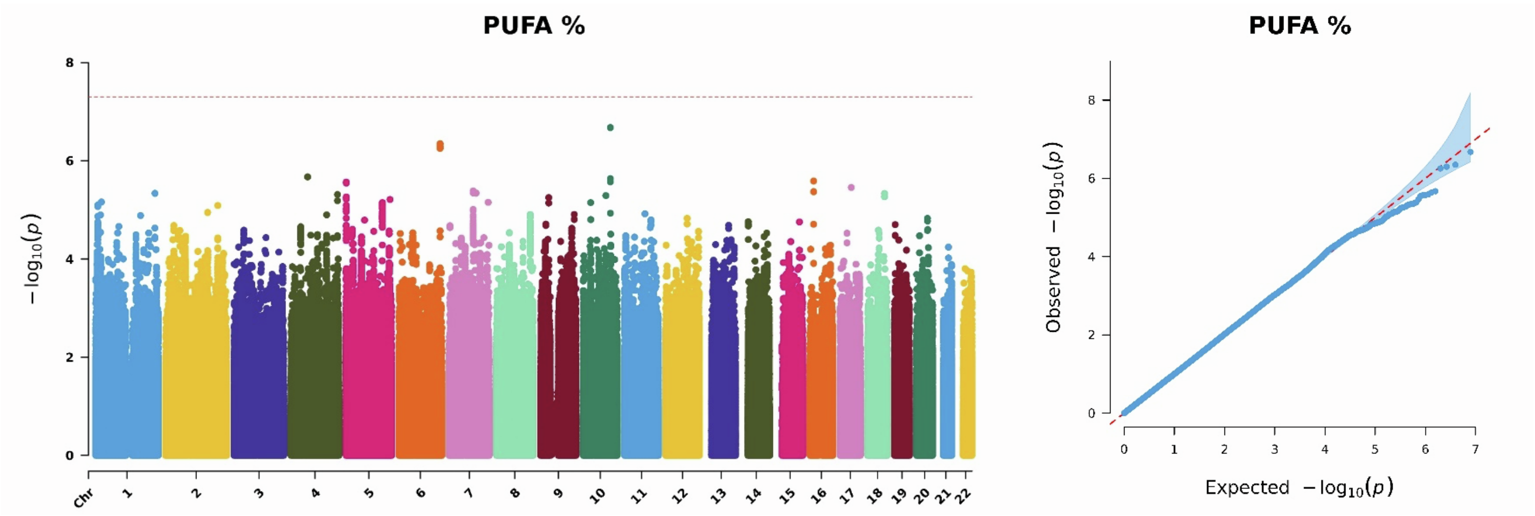

PUFA %
$-\log_{10}(p)$
Chr 1 2 3 4 5 6 7 8 9 10 11 12 13 14 15 16 17 18 19 20 21 22
PUFA %
Observed $-\log_{10}(p)$
Expected $-\log_{10}(p)$

H

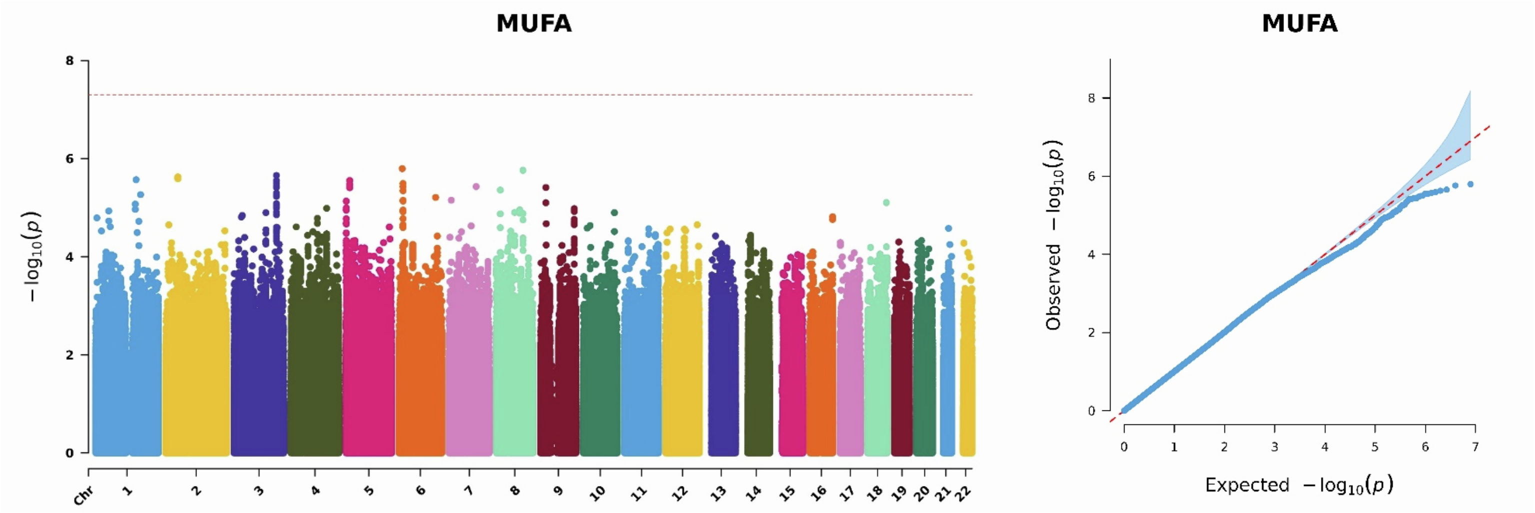

MUFA
$-\log_{10}(p)$
Chr 1 2 3 4 5 6 7 8 9 10 11 12 13 14 15 16 17 18 19 20 21 22
MUFA
Observed $-\log_{10}(p)$
Expected $-\log_{10}(p)$

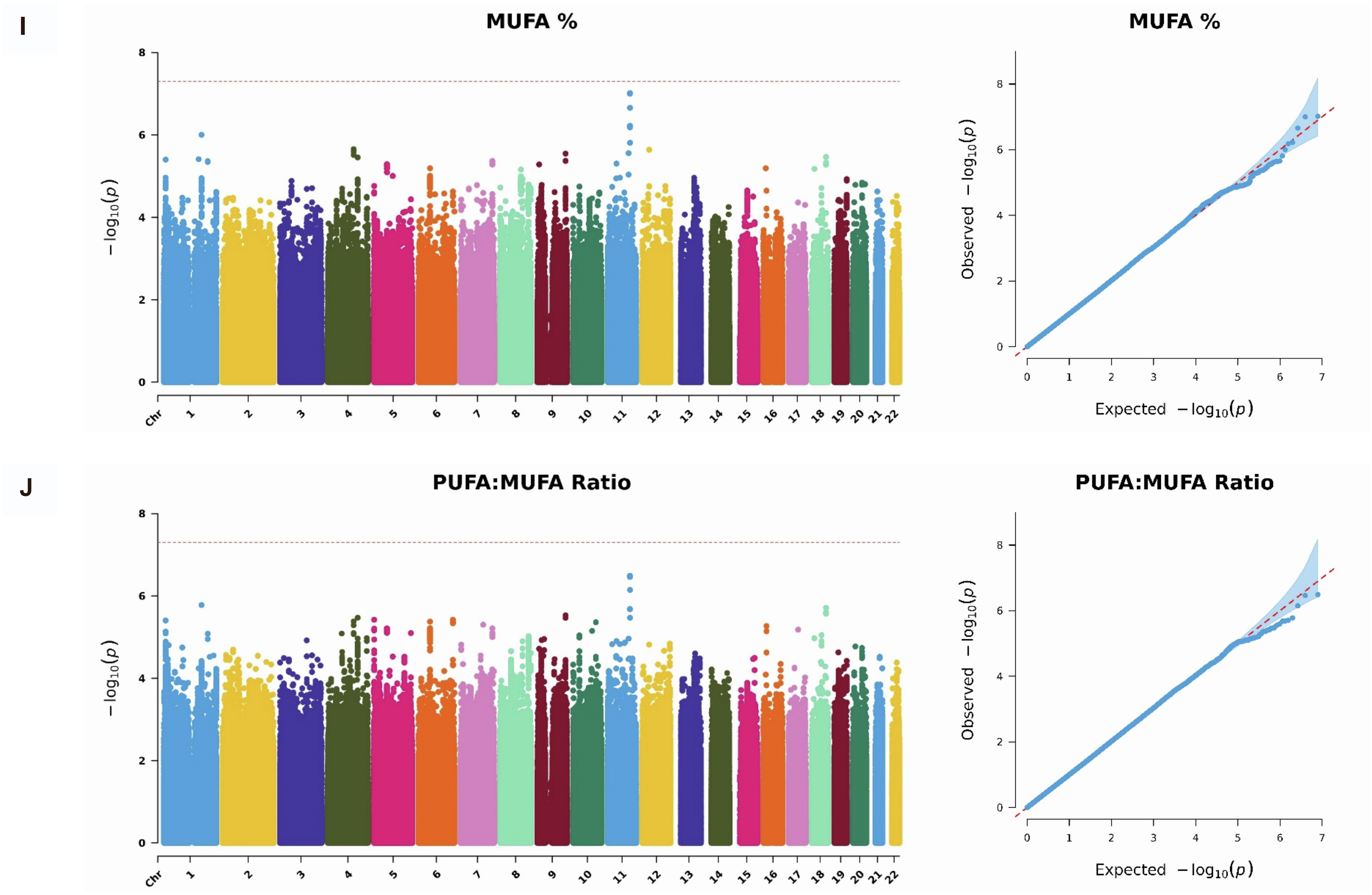
I
MUFA %
MUFA %
$-\log_{10}(p)$
Observed $-\log_{10}(p)$
Expected $-\log_{10}(p)$
Chr 1 2 3 4 5 6 7 8 9 10 11 12 13 14 15 16 17 18 19 20 21 22
J
PUFA:MUFA Ratio
PUFA:MUFA Ratio
$-\log_{10}(p)$
Observed $-\log_{10}(p)$
Expected $-\log_{10}(p)$
Chr 1 2 3 4 5 6 7 8 9 10 11 12 13 14 15 16 17 18 19 20 21 22

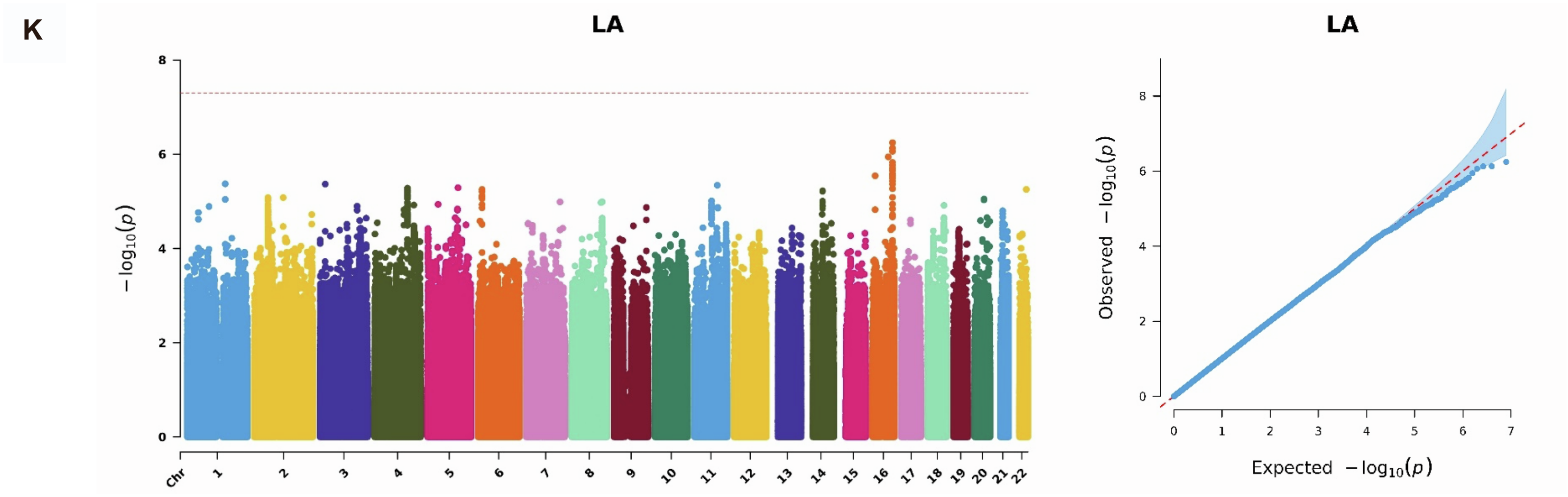
K
LA
$-\log_{10}(p)$
Chr
1
2
3
4
5
6
7
8
9
10
11
12
13
14
15
16
17
18
19
20
21
22
LA
Observed $-\log_{10}(p)$
Expected $-\log_{10}(p)$

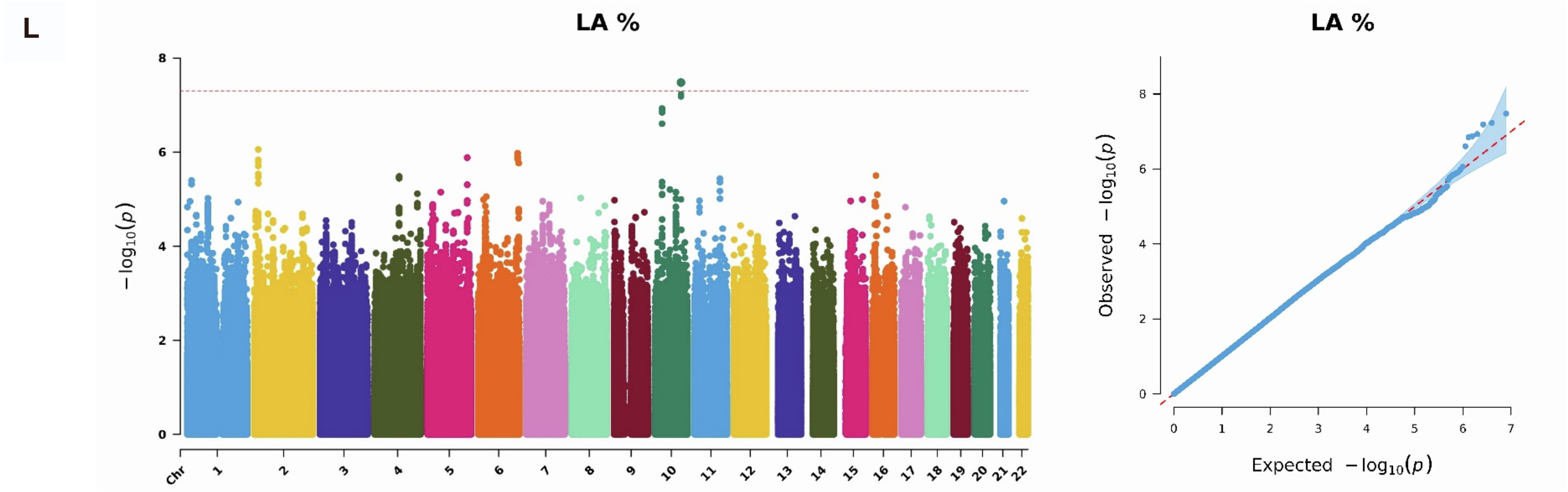
L
LA %
$-\log_{10}(p)$
Chr
1
2
3
4
5
6
7
8
9
10
11
12
13
14
15
16
17
18
19
20
21
22
LA %
Observed $-\log_{10}(p)$
Expected $-\log_{10}(p)$

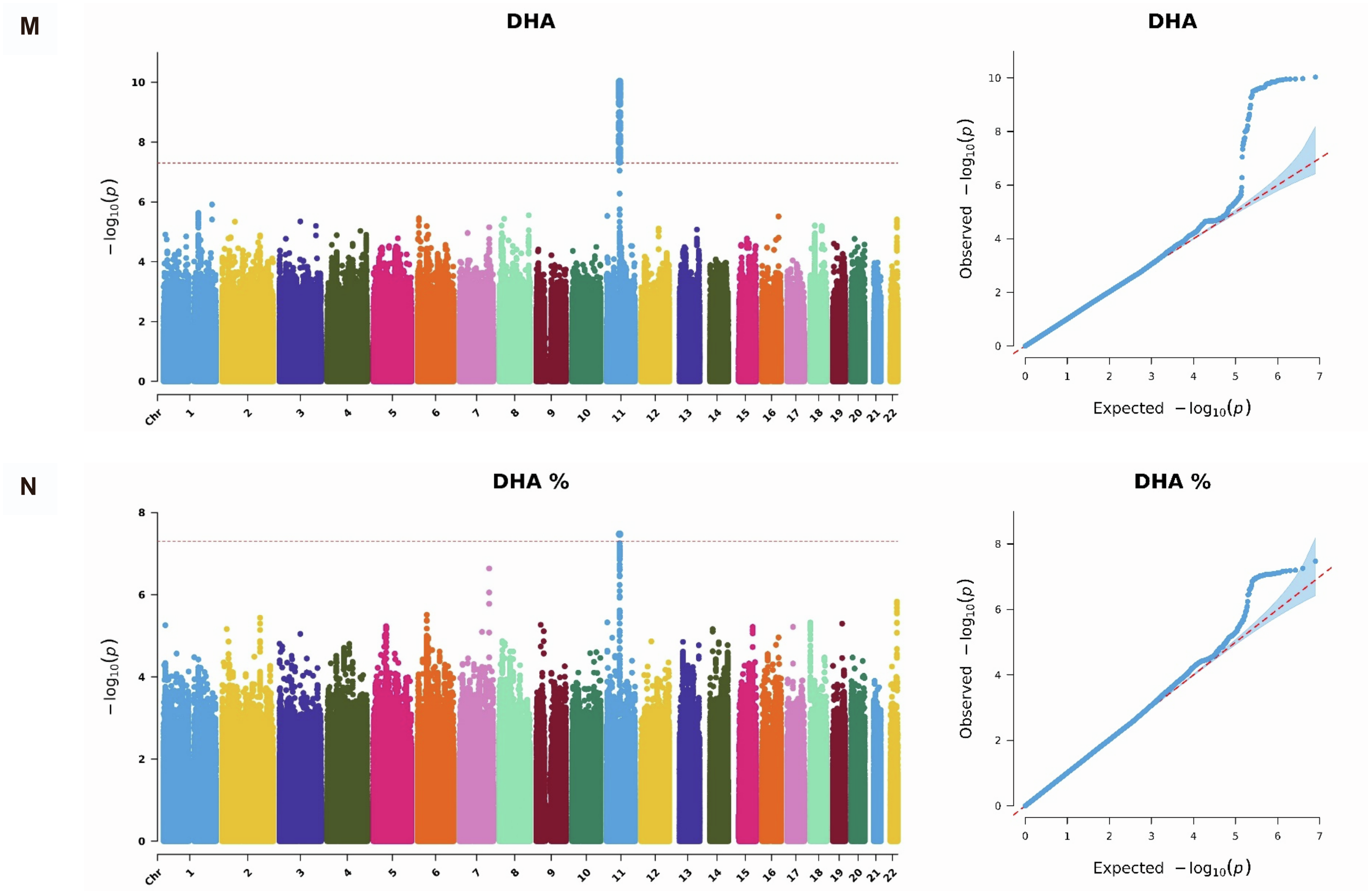

Figure S4. Manhattan and QQ plots of *p*-values for gene-fish oil interactions in 14 PUFAs and MUFAs-related phenotypes for 114,352 participants in the Phase Two dataset

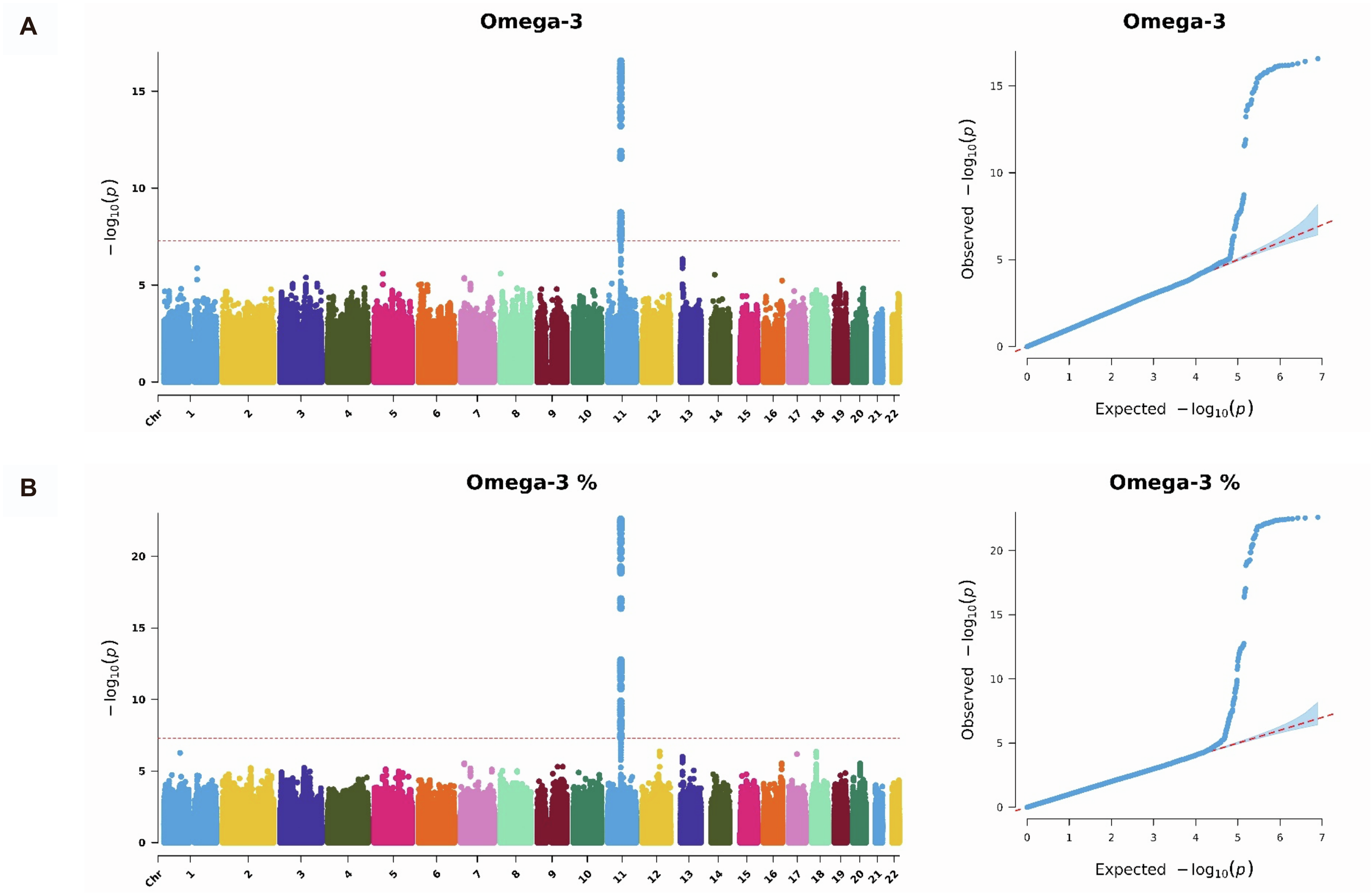
A
Omega-3
Omega-3
B
Omega-3 %
Omega-3 %
$-\log_{10}(p)$
Chr
Observed $-\log_{10}(p)$
Expected $-\log_{10}(p)$

C

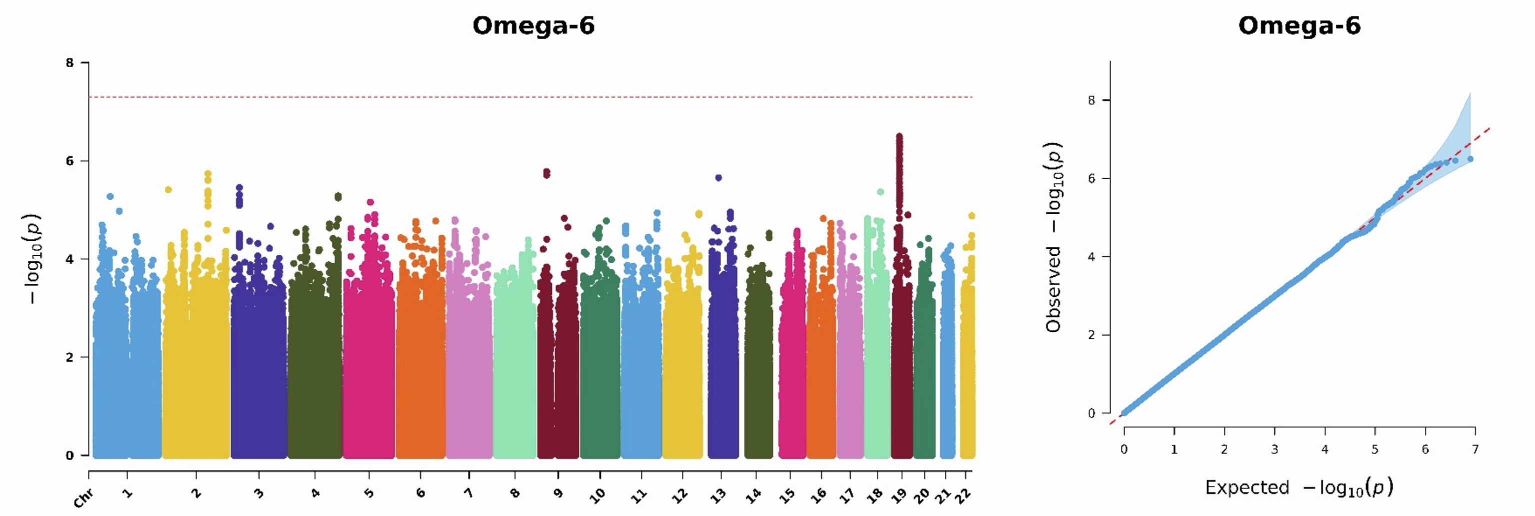
Omega-6
$-\log_{10}(p)$
Chr 1 2 3 4 5 6 7 8 9 10 11 12 13 14 15 16 17 18 19 20 21 22
Omega-6
Observed $-\log_{10}(p)$
Expected $-\log_{10}(p)$

D

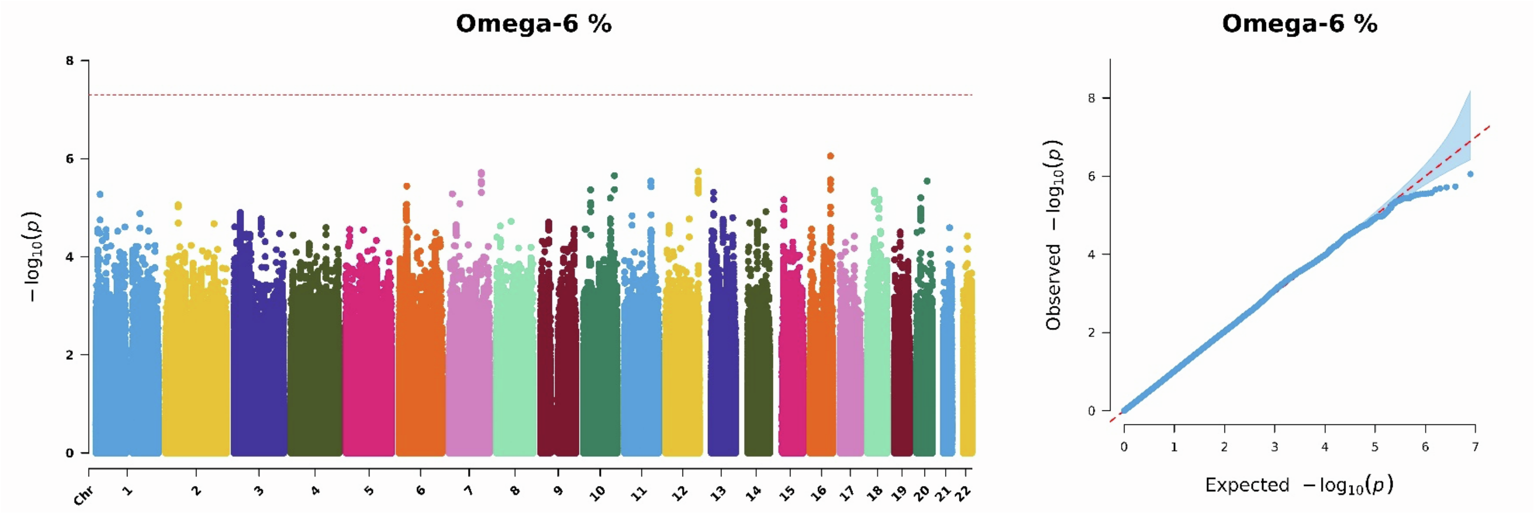
Omega-6 %
$-\log_{10}(p)$
Chr 1 2 3 4 5 6 7 8 9 10 11 12 13 14 15 16 17 18 19 20 21 22
Omega-6 %
Observed $-\log_{10}(p)$
Expected $-\log_{10}(p)$

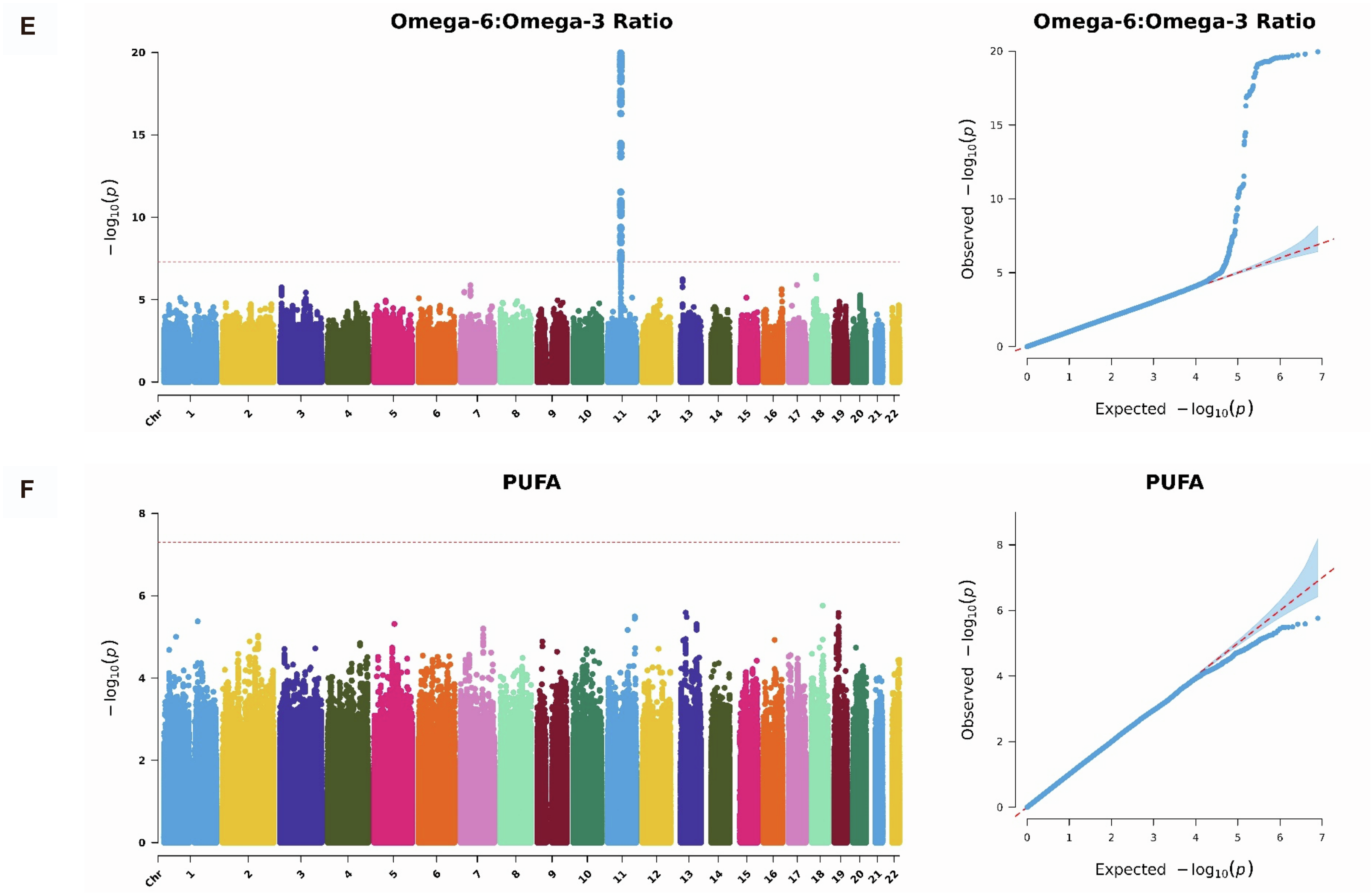
E
Omega-6:Omega-3 Ratio
Omega-6:Omega-3 Ratio
$-\log_{10}(p)$
Chr
Observed $-\log_{10}(p)$
Expected $-\log_{10}(p)$
F
PUFA
PUFA
$-\log_{10}(p)$
Chr
Observed $-\log_{10}(p)$
Expected $-\log_{10}(p)$

G

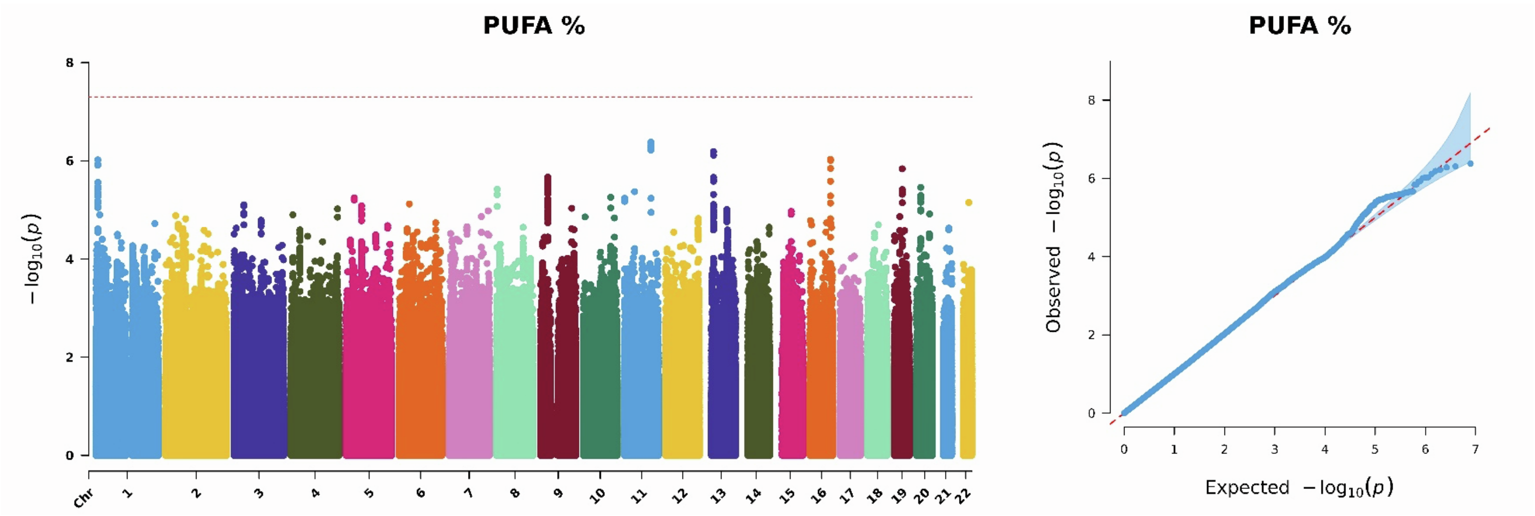
PUFA %
PUFA %
$-\log_{10}(p)$
Observed $-\log_{10}(p)$
Expected $-\log_{10}(p)$
Chr
1
2
3
4
5
6
7
8
9
10
11
12
13
14
15
16
17
18
19
20
21
22

H

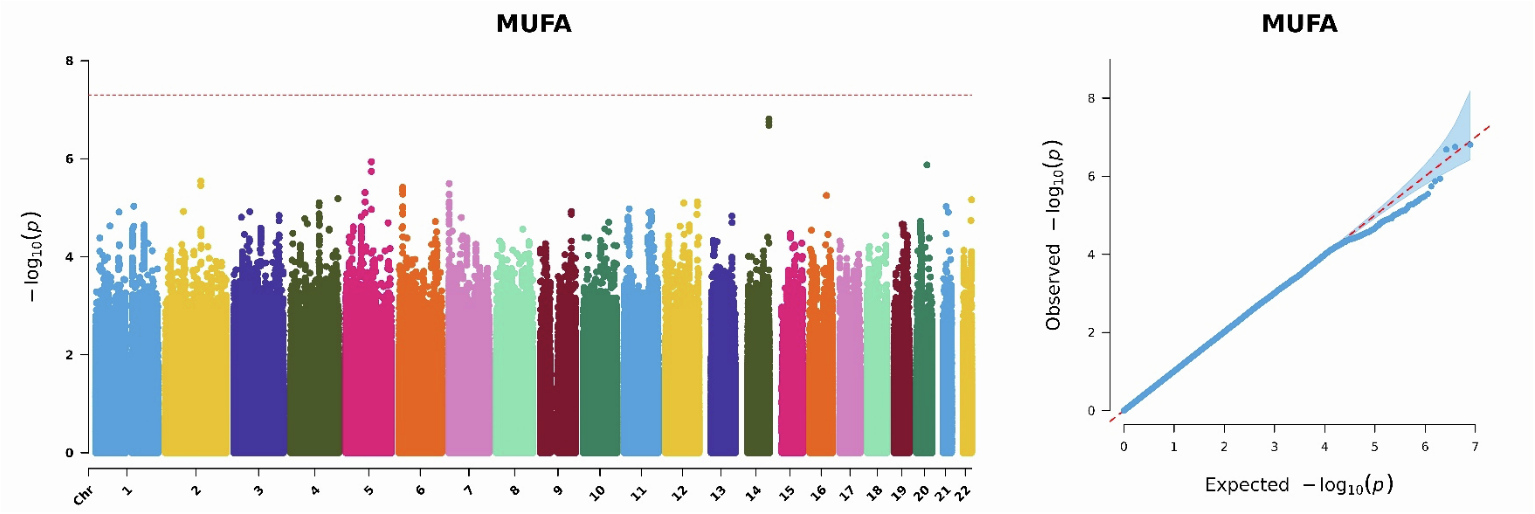
MUFA
MUFA
$-\log_{10}(p)$
Observed $-\log_{10}(p)$
Expected $-\log_{10}(p)$
Chr
1
2
3
4
5
6
7
8
9
10
11
12
13
14
15
16
17
18
19
20
21
22

I

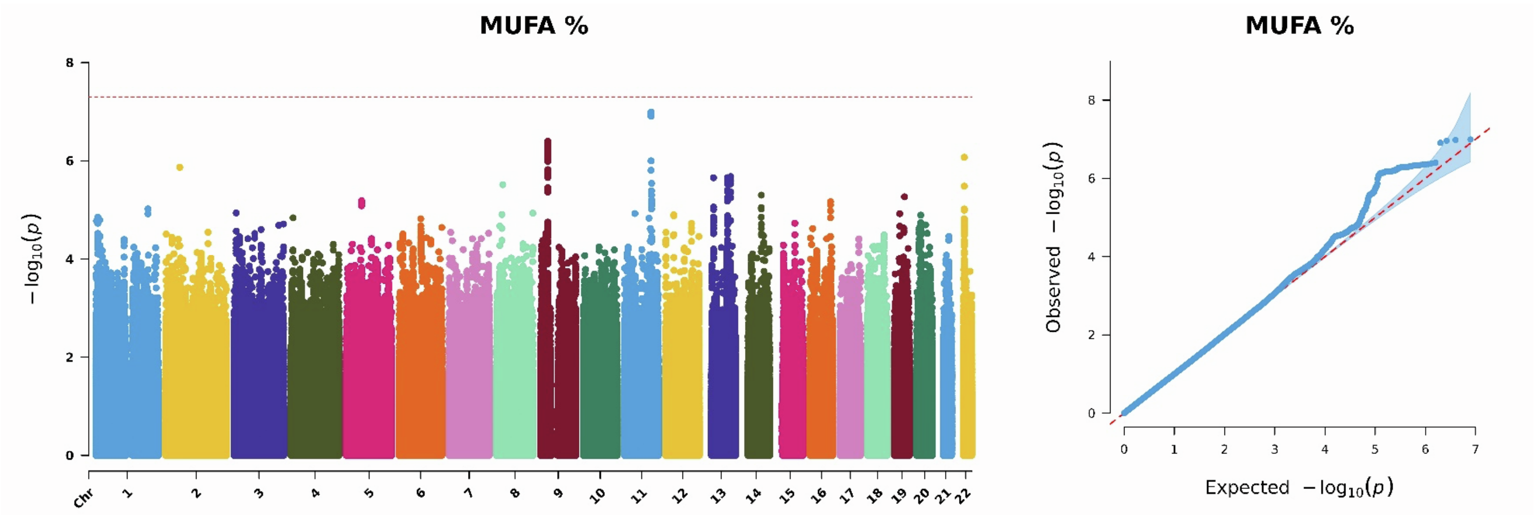
MUFA %
MUFA %
$-\log_{10}(p)$
Chr
Observed $-\log_{10}(p)$
Expected $-\log_{10}(p)$

J

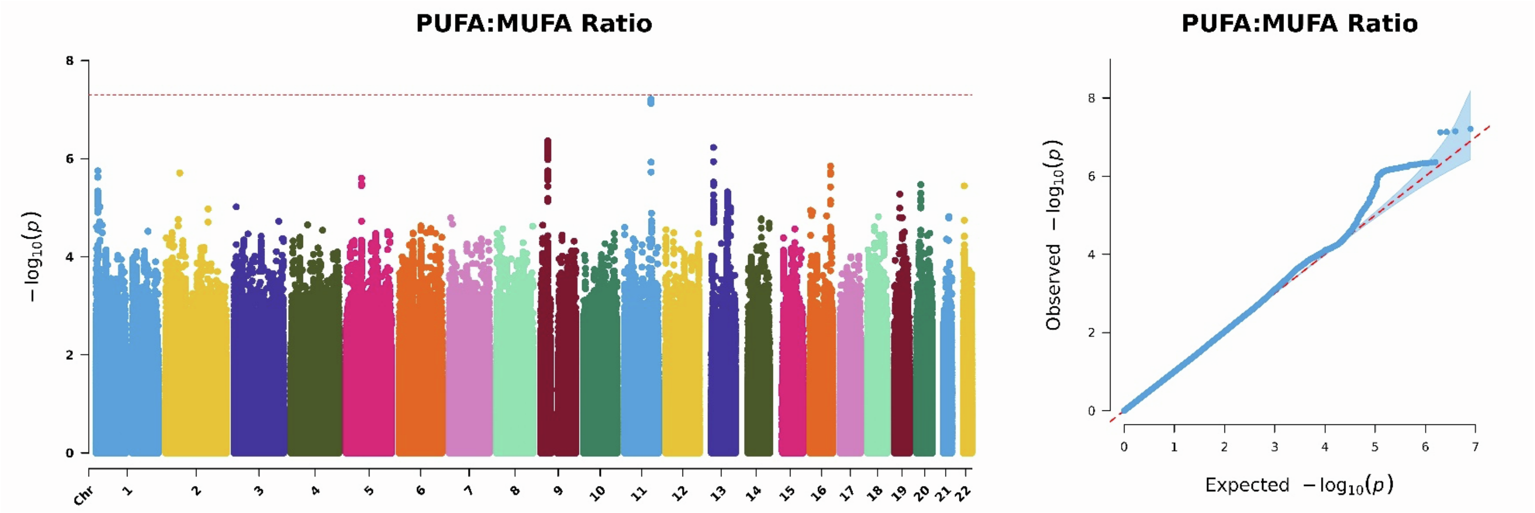
PUFA:MUFA Ratio
PUFA:MUFA Ratio
$-\log_{10}(p)$
Chr
Observed $-\log_{10}(p)$
Expected $-\log_{10}(p)$

K

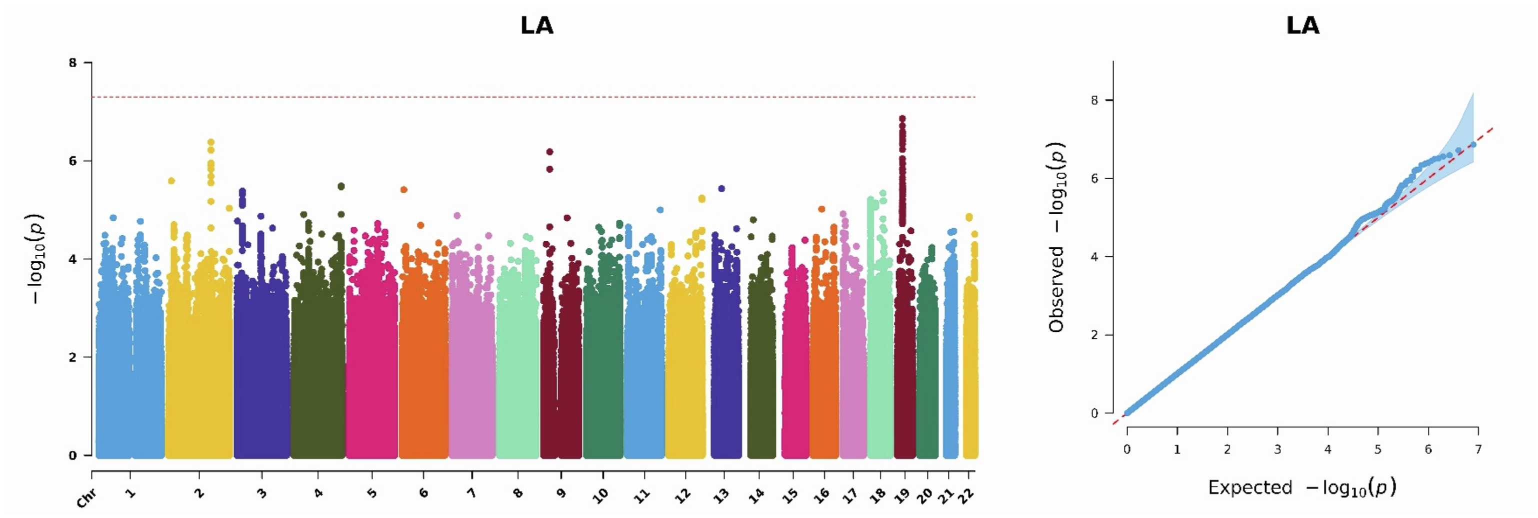

LA
$-\log_{10}(p)$
Chr 1 2 3 4 5 6 7 8 9 10 11 12 13 14 15 16 17 18 19 20 21 22
LA
Observed $-\log_{10}(p)$
Expected $-\log_{10}(p)$

L

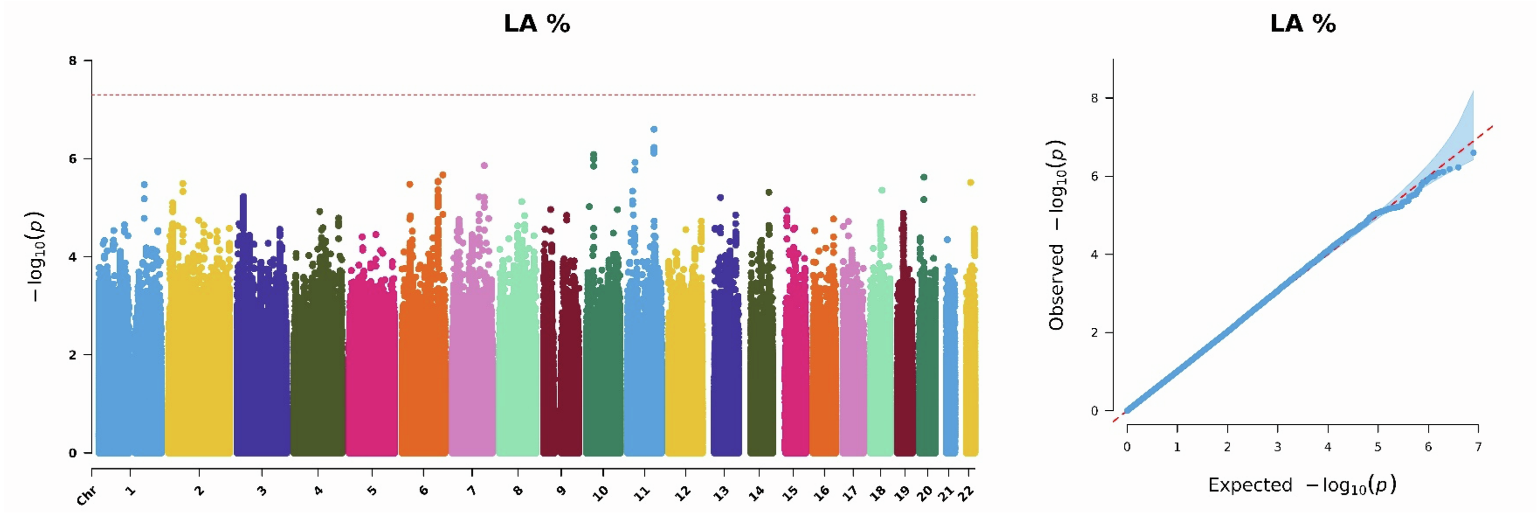

LA %
$-\log_{10}(p)$
Chr 1 2 3 4 5 6 7 8 9 10 11 12 13 14 15 16 17 18 19 20 21 22
LA %
Observed $-\log_{10}(p)$
Expected $-\log_{10}(p)$

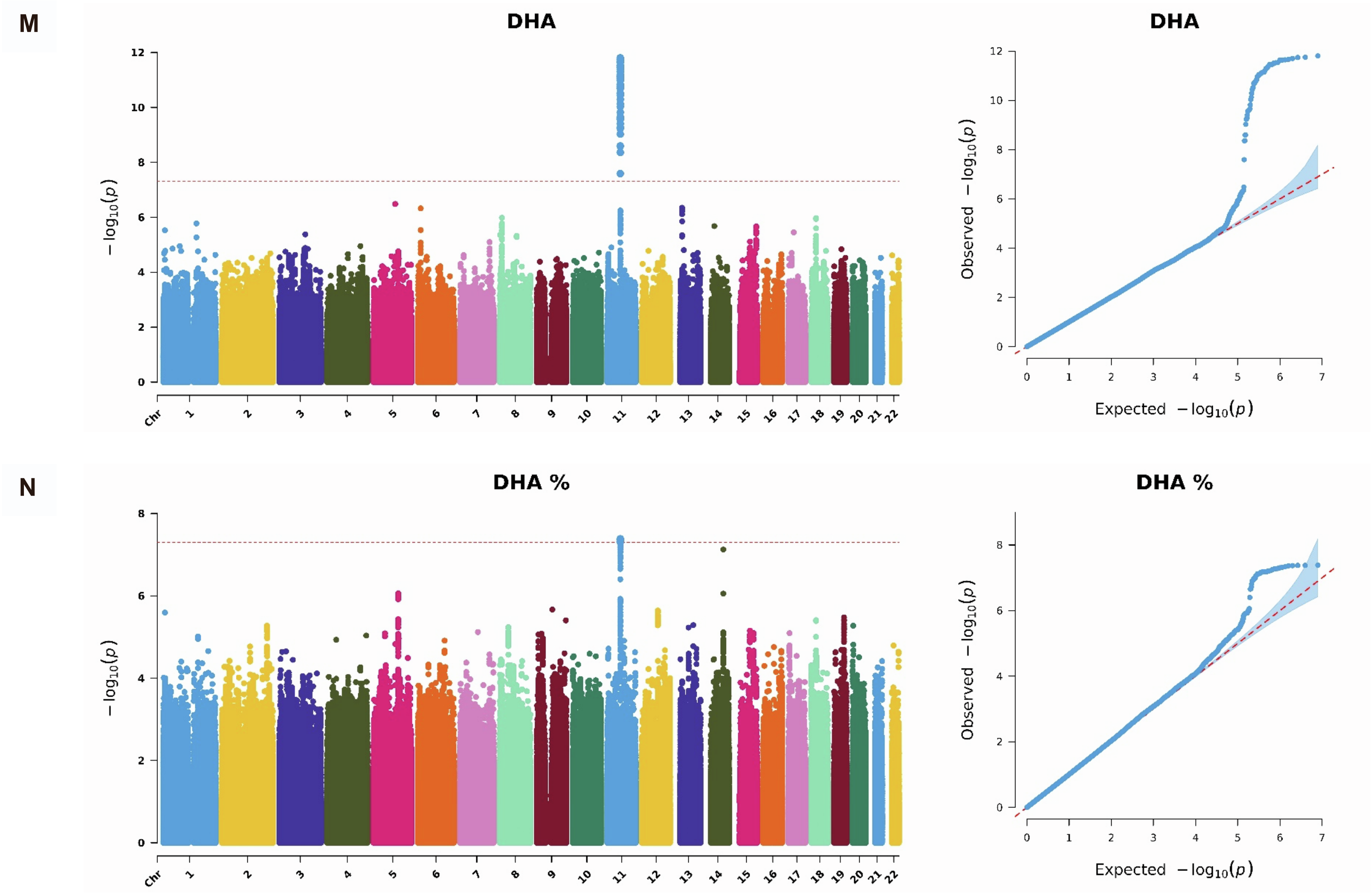

Figure S5. Manhattan and QQ plots of *p*-values for gene-fish oil interactions in 14 PUFAs and MUFAs-related phenotypes for 200,060 participants in the combined dataset

Table S1. Baseline characteristics of participants in Phase One, Phase Two, and combined releases of NMR metabolite data of individuals of European ancestry in UK Biobank

	Phase One (*n* = 85,708)	Phase Two (*n* = 114,352)	Combined phases (*n* = 200,060)
Age, years (SD)	57 (8.01)	57 (8.00)	57 (8.01)
Sex, female (%)	45,907 (54)	61,137 (54)	107,044 (54)
Fish oil intake, *n* (%)			
Yes	27,201 (32)	77,842 (32)	63,711 (32)
No	58,507 (68)	36,510 (68)	136,349 (68)
Absolute PUFA concentrations, mmoL/L (SD)	0.52 (0.22)	0.54 (0.22)	0.53 (0.22)
Omega-3	4.44 (0.67)	4.56 (0.69)	4.51 (0.69)
Omega-6	0.23 (0.082)	0.24 (0.085)	0.24 (0.083)
DHA	3.41 (0.67)	3.51 (0.69)	3.46 (0.69)
LA	2.83 (0.81)	2.98 (0.85)	2.92 (0.84)
MUFA	4.97 (0.79)	5.10 (0.82)	5.04 (0.81)
PUFA			
Proportion in total fatty acids, % (SD)			
Omega-3	4.39 (1.53)	4.35 (1.53)	4.37 (1.53)
Omega-6	37.97 (3.59)	37.57 (3.58)	37.74 (3.59)
DHA	2.00 (0.66)	1.98 (0.67)	1.99 (0.67)
LA	28.94 (3.40)	28.71 (3.38)	28.81 (3.39)
MUFA	23.55 (2.67)	23.97 (2.65)	23.79 (2.67)
PUFA	42.37 (3.73)	41.92 (3.72)	42.11 (3.73)
Ratios (SD)			
Omega-6 to Omega-3	9.82 (4.28)	9.81 (4.23)	9.81 (4.25)
PUFA to MUFA	1.84 (0.35)	1.78 (0.33)	1.81 (0.34)
