# Supplementary material for: A multi-level gene-diet interaction analysis of fish oil and 14 polyunsaturated fatty acid traits identifies the FADS and GPR12 loci
Source: HGG Adv. 2025 May 21;6(3):100459. doi: 10.1016/j.xhgg.2025.100459 (PMC12172259; doi:10.1016/j.xhgg.2025.100459)
Supplement: Document S2. Article plus supplemental information [file mmc3.pdf]

HGG Advances

ARTICLE

A multi-level gene-diet interaction analysis of fish oil and 14 polyunsaturated fatty acid traits identifies the *FADS* and *GPR12* loci

Susan Adanna Ihejirika,[1] Alexandra Huong Chiang,[2] Aryaman Singh,[2] Eunice Stephen,[2] Han Chen,[3] and Kaixiong Ye[1,2,4,*]

Summary

Fish oil supplements (FOS) are known to alter circulating levels of polyunsaturated fatty acids (PUFAs) but in a heterogeneous manner across individuals. These varied responses may result from unidentified gene-FOS interactions. To identify genetic factors that interact with FOS to alter the circulating levels of PUFAs, we performed a multi-level genome-wide interaction study (GWIS) of FOS on 14 plasma measurements in 200,060 unrelated European-ancestry individuals from the UK Biobank. From our single-variant tests, we identified genome-wide significant interacting SNPs ($p < 5 \times 10^{-8}$) in the *FADS1-FADS2* gene cluster for total omega-3, omega-3%, docosapentaenoic acid (DHA), DHA%, and the omega-6 to omega-3 ratio. Among the interaction signals for omega-3%, the lead SNP, rs35473591 (C>CT, CT allele frequency = 0.34), had a lower association effect size in the FOS-taking group ($\beta = 0.35$ for allele C) than that in the group without FOS ($\beta = 0.42$). Likewise, the effect sizes of associations between FOS and omega-3% varied across the three genotype groups ($\beta = 0.45$, 0.50, and 0.59, respectively, in C/C, C/CT, and CT/CT). Our gene-level aggregate and transcriptome-wide interaction analyses identified significant signals at two loci around *FADS1-FADS2* and *GPR12*. The contribution of genome-wide gene-FOS interactions to phenotypic variance was statistically significant in omega-3-related traits. This systematic gene-FOS GWIS contributes to our understanding of the genetic architecture of circulating PUFAs underlying FOS response and informs personalized dietary recommendations.

Introduction

Genetic[1–3] and environmental factors such as diet[4–6] influence the circulating levels of polyunsaturated fatty acids (PUFAs). PUFAs are FAs with 18–24 carbons and multiple double bonds. They fall into two main families, omega-3 and omega-6, depending on which carbon the first double bond falls on from the methyl end of the carbon chain. PUFAs play crucial roles in various physiological processes, including immune function, cardiovascular health, brain development, and cognitive performance.[7–9] α-Linolenic acid (ALA) and linoleic acid (LA) are essential omega-3 and omega-6 FAs, respectively, that can only be obtained from dietary sources and act as precursors for synthesizing long-chain PUFAs.[7] Long-chain PUFAs can be obtained from the diet or produced endogenously in the presence of the necessary precursors.[7] Fish oil supplements (FOS) are a rich, readily available source of long-chain omega-3 PUFAs, particularly eicosapentaenoic acid (EPA) and docosapentaenoic acid (DHA).[10] Additionally, FOS intake alters circulating PUFA concentrations similarly to fish consumption.[11]

The interactions between genetic variants and dietary PUFAs also influence circulating PUFA concentrations.[7,8,12–19] This means that omega-3 PUFAs obtained through FOS can alter the genetic effects on circulating PUFA levels, and that genetic variants can modify the effects of FOS on the same phenotypes. One of the relevant genetic loci, the *FADS* locus, contains the *FADS1* and *FADS2* genes, which encode the delta-5 (D5) and delta-6 (D6) desaturase enzymes, respectively. These membrane-bound enzymes introduce *cis* double bonds at the D5 and D6 positions in PUFAs, the rate-limiting steps in long-chain PUFA biosynthesis.[20,21] Juan et al. found that higher dietary EPA and DHA consumption increases circulating EPA proportions.[12] However, genotypes at the *FADS1* rs174546 SNP (C>T) impacted the extent of that increase. For every 1-standard deviation (SD) increase in EPA intake, individuals with the C/C genotype had an average increase of 3.7% in the circulating EPA level, while those with the T/T genotype had an average increase of 7.8%. These gene-diet interactions may explain some of the heterogeneity in circulating PUFA levels in response to omega-3 PUFA supplementation.[22] Furthermore, these genotype-dependent responses may provide some insights into the inconsistent results in observational studies and randomized controlled trials investigating the effect of omega-3 PUFA supplementation on various outcomes,[8] such as the risk of cardiometabolic diseases, Alzheimer's disease, cancer, and inflammation.[23–27]

[1]Institute of Bioinformatics, University of Georgia, Athens, GA 30602, USA; [2]Department of Genetics, University of Georgia, Athens, GA 30602, USA; [3]Human Genetics Center, Department of Epidemiology, School of Public Health, The University of Texas Health Science Center at Houston, Houston, TX 77030, USA
[4]Lead contact
*Correspondence: kaixiong.ye@uga.edu
https://doi.org/10.1016/j.xhgg.2025.100459.
© 2025 The Author(s). Published by Elsevier Inc. on behalf of American Society of Human Genetics.
This is an open access article under the CC BY license (http://creativecommons.org/licenses/by/4.0/).

Despite advances in characterizing genetic loci interacting with dietary omega-3 PUFAs, most existing gene-dietary PUFAs interaction studies have various weaknesses and limitations. Most of them examined only candidate genes or variants.[12–18] Some were performed in specific contexts, such as disease risk and related outcomes,[19,23–27] while others only examined circulating metabolites other than PUFAs as outcomes.[19,28] They were also limited by small sample sizes. This leaves a gap in understanding the effects of interactions between genome-wide loci and dietary omega-3 PUFAs on circulating PUFAs in a generally healthy cohort.

To this end, we conducted a large-scale gene-diet interaction analysis ($N = 200{,}060$) of FOS on 14 circulating PUFAs-related measurements in the UK Biobank cohort[29,30] to systematically identify genetic factors that interact with FOS to alter levels of plasma PUFAs. We then performed a transcriptome-wide interaction study (TWIS) to identify genes whose expression interacts with FOS. Lastly, we estimated the phenotypic variance explained by genome-wide interactions with FOS. Our study is a large-scale, systematic genome-wide interaction study (GWIS) of FOS on the circulating levels of PUFAs.

Subjects and methods

Cohort

The UK Biobank is a large, population-based prospective study of over 500,000 volunteer participants recruited between 2006 and 2010 from across England, Wales, and Scotland. The participants were between the ages of 40 and 69 years. At recruitment, participants provided sociodemographic, lifestyle, environmental, clinical, and biochemical information through touchscreen questionnaires, face-to-face interviews, and physiological measurements. Plasma samples were provided for genotyping and nuclear magnetic resonance (NMR) spectroscopy-based metabolomic measurements.[29,30] The UK Biobank received ethical approval from the National Research Ethics Service Committee North West–Haydock (reference ID 11/NW/0382). The use of participants' data in this study was approved under Project 48818.

Participant inclusion and exclusion

In this study, we restricted our participants to those who were of genetically determined European ancestry,[31] did not have a high degree of kinship with other volunteers, were not outliers for heterozygosity or missing genotype rate, had matched self-reported and genetic sex, and had no sex chromosome aneuploidy (Figure S1). All UK Biobank participants provided written informed consent upon recruitment, authorizing the storage and use of their provided biological, medical, and genetic data for health-related research purposes. Additionally, participants who had withdrawn their consent as of February 22, 2022 were removed ($N = 114$).

Genotype

The genotype data used in this study was version 3 of the UK Biobank genotyping release, previously described in detail.[29] These data included ~820,000 variants genotyped with either the UK BiLEVE Axiom Array or the UK Biobank Axiom Array. It also included initial quality control and imputation of variants using a merged reference panel of the Haplotype Reference Consortium and UK10K.[29] For further genotype quality control, we selected only autosomal variants with imputation quality score >0.5, minor allele frequency (MAF) >1%, genotype missingness per individual <5%, genotype missingness per variant <2%, and Hardy-Weinberg equilibrium $p < 1.0 \times 10^{-6}$ using PLINK2 alpha version 2.3.[32]

Phenotype

Finland-based Nightingale Health performed NMR metabolomic measurement of 251 metabolic biomarkers for approximately 280,000 participants. The UK Biobank released these data in two phases. Phase one release consisted of plasma measurements for 120,000 randomly selected samples. Phase two consisted of measurements for individuals in phase one and an additional 170,000 samples. Absolute circulating levels of FAs, including PUFAs and monounsaturated FAs (MUFAs), were measured and reported in mmol/L. The proportion of each PUFA in total FAs was expressed as a percentage. We investigated 14 circulating PUFAs and MUFA-related traits as our outcomes. The 14 phenotypes were the absolute circulating levels of total PUFAs, total MUFAs, omega-3 PUFAs, omega-6 PUFAs, LA, and DHA; their relative percentages in total FAs (i.e., PUFAs%, MUFAs%, omega-3%, omega-6%, LA%, and DHA%); and the ratios of PUFAs to MUFAs and omega-6 to omega-3. All phenotypes were rank-based inverse normal transformed.

Dietary exposure

The primary exposure of interest in this study was FOS. We used the UK Biobank dietary questionnaire administered during recruitment to determine the exposure status. This questionnaire was a touchscreen food frequency questionnaire (FFQ) about the intake frequency of common food and drink items. A total of 497,666 participants filled these out during the initial assessment center visit between 2006 and 2010. Participants were asked about minerals and other dietary supplements: "Do you regularly take any of the following? (You can select more than one answer)," and "fish oil (including cod liver oil)" was one of the options (data field 6179).

GWIS analysis

We performed single-variant interaction tests with the gene-environment interaction analysis in millions of samples (GEM) tool.[33] GEM implements an additive, generalized linear model for unrelated individuals. GEM conducts tests for marginal genetic effects, interaction effects (1-degree of freedom [1df] tests), and joint tests for genetic main and interaction effects (2-degrees of freedom tests). In this analysis, only results from the 1df test were interpreted due to our interest in strong interaction effects.[34] We used age, sex, age-by-sex, and the first 10 principal components as the covariates in our model. The age-by-sex covariate was included to account for potential interaction effects between age and sex that may influence metabolite levels in a non-additive manner, as recommended in the literature.[35] Genome-wide significance at p value of 5.0×10^{-8} was used to define significant interactions.

To interpret the interaction signals identified by GEM, we used PLINK2 alpha version 2.3 to conduct exposure-stratified analysis to quantify SNP effects in each exposure group. Lastly, to quantify

the effects of FOS across the genotype groups, we fitted linear models to each group and adjusted for the same covariates as in our single-variant interaction tests.

Sensitivity analysis

We conducted two sensitivity analyses to evaluate the robustness of our genome-wide significant findings to other sources of dietary omega-3 intakes. In our first analysis, we included oily fish intake as a covariate in addition to the previously described covariates. In the same touchscreen FFQ administered to UK Biobank participants at recruitment, participants were asked, “How often do you eat oily fish? (e.g., sardines, salmon, mackerel, herring),” to which participants had the answer options of “never,” “less than once a week,” “once a week,” “2–4 times a week,” “5–6 times a week,” “once or more daily,” “do not know,” and “prefer not to answer” (data field 1329). We then combined the “2–4 times a week,” “5–6 times a week,” and “once or more daily” options into a new category of “twice or more times a week” and the “do not know” and “prefer not to answer” options into “unknown.”[34] We referred to this model as sensitivity model 1. For our second sensitivity analysis, we included the estimated omega-3 intake as an additional covariate (data field 26015). We included participants with at least one omega-3 estimate and used the mean for those with estimates from multiple responses. We referred to this model as sensitivity model 2. We then ran pairwise Z tests to determine whether the differences across models were statistically significant.

Gene-level interaction analysis

FUMA[36] was used to implement the MAGMA model,[37] which maps variants to genes based on physical locations, aggregates p values from the individual variant-FOS interaction tests across genic regions, and tests gene-level associations with each of the phenotypes of interest. Variants were mapped to 18,767 genes based on defined transcription start and stop sites, and a p value of 2.66×10^{-6} (0.05/18,767) was used to define statistical significance. Linkage disequilibrium (LD) was estimated using the UK Biobank release 2b 10k White British reference panel.

TWIS analysis

We used the MetaXcan framework for our TWIS to integrate expression quantitative trait locus (eQTL) information with single-variant GWIS summary statistics. MetaXcan tools include S-PrediXcan[38] and S-MultiXcan.[39] S-PrediXcan predicts and imputes genetically determined gene expression levels and tests their associations with the phenotype of interest. We used the MASHR-based models to predict gene expression. These models are based on fine-mapped variants for each tissue. They take in gene expression weights, the variances and covariances of SNPs, and the β coefficient of each SNP from summary statistics. S-MultiXcan aggregates information across all tissues. After multiple testing correction for the number of genes that had a prediction model in at least one tissue, significance was defined as $p < 2.9 \times 10^{-6}$ (0.05/17,500).

Estimation of phenotypic variance explained by gene-FOS interactions

We performed genome-wide complex trait analysis-genomic-relatedness-based restricted maximum-likelihood (GCTA-GREML)[40] analysis to estimate the proportion of phenotypic variance explained by genome-wide gene-FOS interactions for each of the 14 phenotypes separately. We estimated the genetic relationship matrix between pairs of individuals using LD-pruned SNPs. SNPs were pruned using a window size of 1,000 variants, a step size of 100 variants, and an r^2 of 0.9. An estimate of phenotypic variance explained was considered significant if the 95% confidence interval did not include zero; that is, its error bar did not overlap with zero on the bar plot.

To estimate the variance explained by the top variant, we assumed gene-environment independence and applied the formula

$$2\beta^2_{G\times E} f(1-f)p(1-p)$$

where β is the gene-FOS interaction effect size, f is the MAF, and p is the exposure prevalence in the population.

Results

Sample characteristics

NMR metabolite measurements for UK Biobank participants were released in two phases: phase one (N = 120,000) and phase two (N = 170,000 additional samples). The phase two release also included updated and corrected phase one measurements. We refer to the additional 170,000 samples as phase two in this study. We performed exposure quality control on the combined dataset of 290,000 participants for our gene-FOS interaction analysis (Figure S1). Approximately 31.8% (N = 63,711) of 200,060 included participants indicated regular FOS use at recruitment (Table S1). The percentage of females and mean age in our dataset were 53.5% and 56.8 ± 8.0, respectively.

Variant-level analysis identified interacting SNPs in the *FADS* locus

We performed single-variant 1df interaction tests to examine the interactions between FOS and genetic variants in the phase one and phase two datasets separately. We identified one and two unique loci at $p < 5.0 \times 10^{-8}$ in the phase one and phase two datasets, respectively (Figures S2–S4; Tables S2 and S3). The correlations between the p values from both analyses are shown in Figure S3, with reproducible association signals observed for total omega-3, omega-3%, the omega-6:omega-3 ratio, DHA, and DHA%. These reproducible signals, genome-wide significant in phase two and nominally significant in phase one, are located in the *FADS1-FADS2* gene cluster, hereafter referred to as the *FADS* locus.

Next, we performed interaction tests in the combined dataset of phases one and two (Figure S5, Table S4). We identified genome-wide significant interaction effects for variants in the *FADS* locus, for five traits, including total omega-3, omega-3%, DHA, DHA%, and the omega-6:omega-3 ratio (Figures 1A and S5; Table 1). To report our significant results, we focused on omega-3%, as we observed similar trends in the other four traits. Omega-3% encompasses all omega-3 PUFAs and reflects omega-3 concentration in relation to total FAs. The most significant interaction was at the lead SNP rs35473591 (chr11:61586328; C>CT, MAF = 0.34)

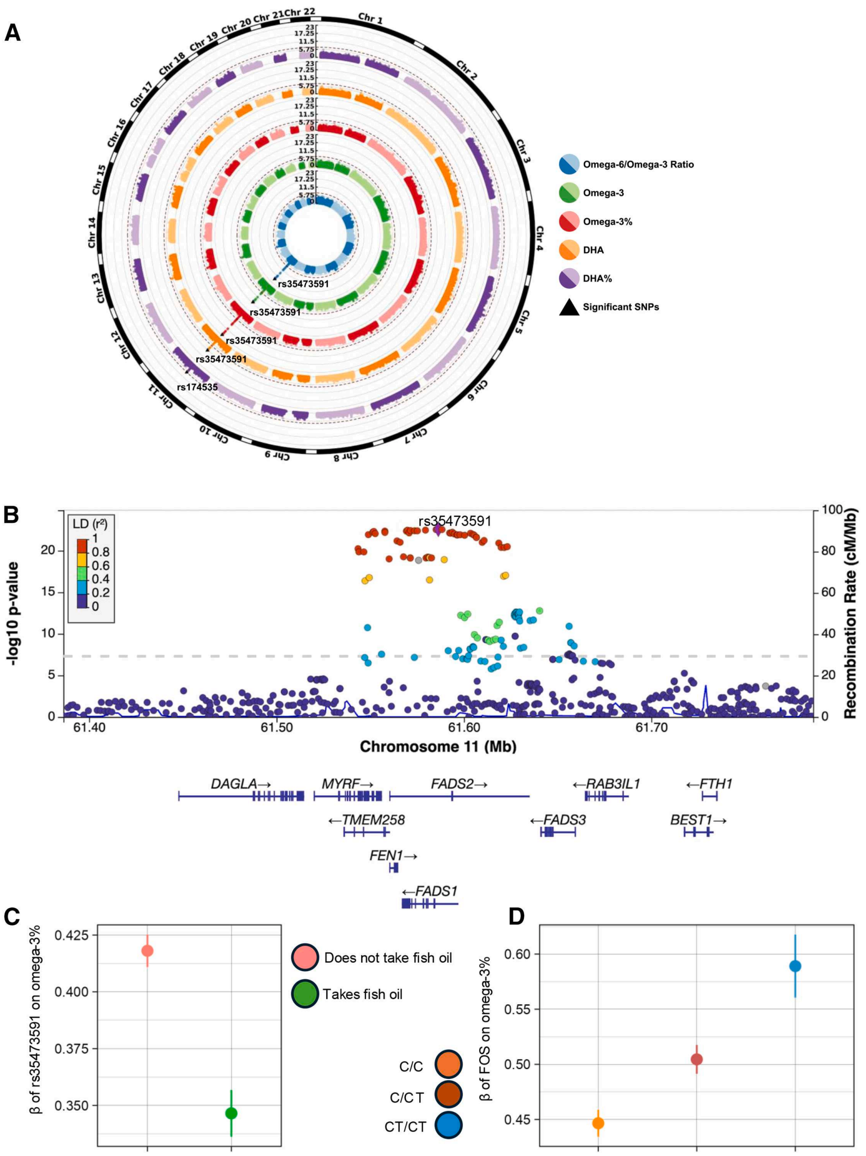

Figure 1. *FADS* variants with significant interaction signals ($p < 5.0 \times 10^{-8}$) in five PUFA traits

(A) Circular Manhattan plots of p values for gene-FOS interactions on omega-3, omega-3%, DHA, DHA%, and the ratio of omega-6:omega-3.

(B) LocusZoom plot of the *FADS* locus for omega-3%.

(C) Marginal genetic effect of rs35473591 on omega-3% in stratified exposure subgroups.

(D) Marginal exposure effects of FOS on omega-3% in stratified genotype subgroups. All error bars represent 95% confidence intervals.

(Figure 1B). We performed a stratified analysis to quantify the genetic effect of rs35473591 in each exposure subgroup (Figure 1C). The C allele had an overall positive effect on omega-3%, but its effect size was lower in the FOS-taking group ($\beta_G = 0.35$, SE $= 0.01$) than in the group that did not use FOS ($\beta_G = 0.42$, SE $= 0.003$). Furthermore, we fitted linear models to quantify the effect of FOS in each genotype subgroup. FOS significantly altered omega-3% levels. The degree of alteration varied across the three genotype groups (Figure 1D; $\beta_E = 0.45$, 0.50, and 0.59 SDs, SE $=$ 0.006, 0.007, and 0.01, respectively, in C/C, C/CT, and CT/CT). Together, these results highlight a significant effect of interaction between rs35473591 and FOS on omega-3%, demonstrating that the effect of FOS on circulating omega-3% is modulated by the rs35473591 genotype. Similar interaction effects were observed at the *FADS* locus for total omega-3, DHA, DHA%, and the omega-6:omega-3 ratio.

We conducted two sensitivity analyses to evaluate the robustness of our genome-wide significant findings at rs35473591 to other sources of dietary omega-3 intakes. We adjusted for oily fish intake in our first analysis (sensitivity model 1) and for estimated omega-3 intake in our second analysis (sensitivity model 2). We found that the gene-FOS interaction estimates at rs35473591 were consistent with results from our primary analysis, and the effect estimates were not statistically different across the primary and sensitivity analysis models (Tables S7–S9).

Gene-level interactions with FOS

To extend our single-variant tests, we first used MAGMA to aggregate interaction p values across genic regions and performed gene-based interaction tests. The *FADS* cluster interaction was significant in the same five traits as in the single-variant tests (Figure 2). Around this *FADS* locus, in addition to the *FADS1* and *FADS2* genes, the *FADS3*, *TMEM258*, *MYRF*, *FEN1*, and *RAB3IL1* genes were also significant across the same five traits (Figure 2A), but these signals may be driven by the same variants due to the high level of LD in this genomic region. Additionally, a locus around the *GPR12* gene was significant in total PUFAs% and the ratio of PUFAs:MUFAs (Figure 2B). The top SNP in PUFAs%, rs1752653 (chr13:27322777; T>C, MAF $= 0.21$), reached suggestive genome-wide significance at $p < 5.0 \times 10^{-6}$ (Figure 3A). *GPR12*-rs1752653 had opposite directions of genetic effects on PUFAs% in the FOS and non-FOS-taking groups, with $\beta_G = 0.03$ and -0.01, respectively (Figure 3B). Across the three genotype groups, the effect of FOS on PUFAs% was $\beta_E = 0.24$, 0.21, and 0.16 SDs and SE $=$ 0.02, 0.01, and 0.012278, respectively, in T/T, T/C, and C/C (Figure 3C).

Next, we integrated eQTL information with our GWIS summary statistics using S-PrediXcan and S-MultiXcan.

Table 1. **Lead SNPs in traits with genome-wide significant interaction signals**

rsID	Chr	Pos	Nearest gene	EA (frequency)	NEA	PUFA trait	Interaction p value	Interaction β	Interaction SE
rs35473591	11	61586328	*FADS1-FADS2*	C (0.66)	CT	omega-3%	2.50×10^{-23}	−0.0722	0.00726
						omega-6:omega-3 ratio	1.09×10^{-20}	0.0672	0.00720
						omega-3	2.74×10^{-17}	−0.0595	0.00704
						DHA	1.56×10^{-12}	−0.0492	0.00696
rs174535	11	61551356	*MYRF*	T (0.65)	C	DHA%	4.11×10^{-8}	−0.0390	0.00718

All significant variants are in the *FADS1-FADS2* gene cluster. Chr, chromosome; EA, effect allele; NEA, non-effect allele; Pos, position; SE, standard error.

We first performed S-PrediXcan analyses to identify genes whose genetically predicted expression levels interact significantly with FOS intake status in each phenotype. S-PrediXcan conducted single-tissue analysis in 49 tissues available in the GTEx Consortium. We used S-MultiXcan to aggregate results across multiple tissues and to increase power relative to the individual tissue analyses. After multiple testing corrections for the number of genes that had a prediction model in at least one tissue (0.05/17,500; $p < 2.9 \times 10^{-6}$), we identified 24 significant interactions involving 7 genes across 5 unique traits (Table S5). The *FADS1*, *FADS2*, *FADS3*, *TMEM258*, *FEN1*, *BEST1*, and *FTH1* genes were significant across the same five traits as MAGMA gene-based analysis and our single-variant tests. The most significant interaction was for the *FADS1* gene ($p = 8.8 \times 10^{-22}$) in omega-3%. The *FADS1* gene was also the only gene with interaction effects in all five phenotypes with significant interactions. In summary, two types of gene-based analyses consistently identified genes around the *FADS* locus to exhibit significant gene-FOS interaction signals for five PUFA traits. Gene-level analysis with MAGMA further identified the *GPR12* locus for total PUFAs% and the ratio of PUFAs to MUFAs.

Contribution of gene-FOS interactions to the phenotypic variance of circulating PUFA levels

We conducted GCTA-GREML analysis to estimate the amount of phenotypic variance explained by genome-wide gene-FOS interactions. The total additive genetic influence (i.e., SNP heritability) across all 14 PUFA and MUFA traits ranged from 14.2% to 23.9% (SE = 0.36% and 0.37%) in LA and MUFAs%, respectively (Figure 4; Table S6). The phenotypic variance explained by genome-wide gene-FOS interactions ranged from 0.53% to 1.51% (SE = 0.38% and 0.39%) in omega-6% to DHA%, respectively. These estimates were statistically significant in 10 out of 14 PUFA traits. The four traits without significant variance explained are total PUFAs, total MUFAs, MUFAs %, and omega-6%. We then estimated the contribution of the top SNP, rs35473591, to the variance explained by gene-FOS interactions in omega-3%. SNP rs35473591 explained a proportion of 5.12×10^{-4}, estimated to be 4.11% of the genome-wide gene-FOS interaction contributions to omega-3% variance. Gene-FOS interactions make small but non-negligible contributions to the phenotypic variance of plasma PUFA traits.

Discussion

In this study, we reported an extensive and systematic GWIS of gene-FOS interactions in 14 PUFAs and MUFAs-related traits. Our goal for this project was to leverage a large cohort of over 200,000 European individuals in the UK Biobank to identify genetic loci that modify the associations between FOS and circulating PUFA traits. We also estimated the contribution of genome-wide interactions with FOS to the phenotypic variance. Across 14 traits, we identified significant gene-FOS interactions for 7 traits—5 variant- and gene-level interactions in omega-3 related traits and the omega-6:omega-3 ratio, and 2 additional gene-level interactions for PUFAs% and PUFAs/MUFAs ratio.

In both our single-variant and gene-level analyses, we replicated the *FADS1-FADS2* gene cluster identified in previous gene-dietary PUFAs interaction studies.[12,41–43] These studies investigated other *FADS* SNPs in high LD with our top SNP, rs35473591. These variants do not all have the same effect on PUFAs, so it is essential to investigate the effect of each SNP independently in the different PUFAs.[7] From our results, the lead *FADS1*-rs35473591 SNP had different effect sizes on omega-3% in the exposure groups. The genetic influence of this SNP was much more substantial in the individuals who do not take fish oil. This shows that FOS lessened the *FADS1*-rs35473591 C allele effect on omega-3%, which is evidence of interaction. We also saw an overall increase in omega-3-related traits and a reduction in the omega-6:omega-3 ratio after supplementation, indicating that FOS increases circulating omega-3 levels, as previously established.[4–6,44] The ratio of dietary omega-6:omega-3 consumed has drastically shifted from ~5:1 to 10:1 because of our modern Western diets, and this is reflected in the ratio of plasma omega-6:omega-3.[45] It is crucial to regulate this ratio because this shift has been associated with various chronic diseases.[8]

Our results showed that FOS intake had the lowest effect on omega-3% levels in the C/C homozygote genotype group at *FADS1*-rs35473591 and had the highest effect in the CT/CT group. The degree of omega-3% alteration increased

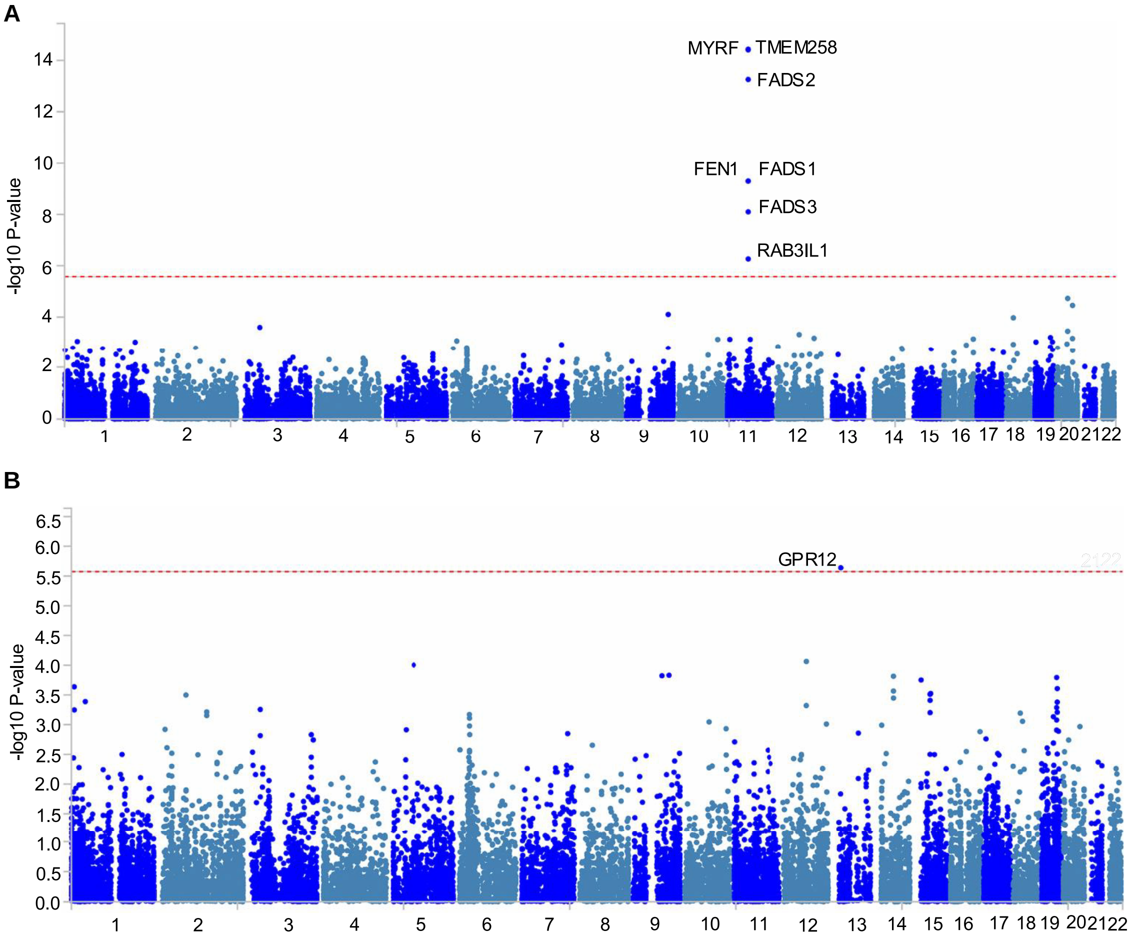

Figure 2. Significant gene-level interactions for omega-3% and PUFAs
Gene-level Manhattan plots from MAGMA analysis for genes that reached the significance threshold of $p < 2.66 \times 10^{-6}$ in (A) omega-3% and (B) PUFAs%.

with each copy of the CT allele. This implies that individuals with the CT/CT and C/CT genotypes will benefit more from taking FOS in terms of increasing omega-3 PUFA levels and reducing their omega-6:omega-3 ratio. Individuals who are homozygous for the C major allele at *FADS1*-rs35473591 benefit less, suggesting that they need higher doses of FOS to attain the same circulating omega-3 levels as CT carriers. In summary, the *FADS1*-rs35473591 SNP, in the presence of FOS, has an increasing effect on omega-3-related traits, but the degree of this effect is genotype dependent. We did not find any significant interaction for *FADS* variants in omega-6-related traits. Previous studies have implicated the *FADS* locus in interactions with PUFA supplementation.[13] A reasonable explanation is that our study focused on omega-3 PUFA supplementation (i.e., FOS), while Sergeant et al.[13] focused on supplementation of γ-linolenic acid supplementation, which is an omega-6, 18-carbon PUFA.

Taking only genetic main or marginal effects into account does not give a complete picture of genetic impacts on circulating PUFAs. Accounting for relevant gene-environment interactions revealed patterns that would not be observed in a typical genome-wide association study. Furthermore, revealing gene-environment interactions is needed to estimate how much phenotypic variance is explained by such interactions. This estimation gives a better understanding of complex traits and diseases because they are influenced by a combination of genetic makeup and environmental exposures, and the effects of some genes are environment dependent. It also helps to explain some of the so-called missing heritability in traits. From our results, additive genetic factors alone explain a maximum of 22.5% of the phenotypic variance of omega-3-related plasma levels, while gene-FOS interactions explain a maximum of 1.51%. The contributions of gene-FOS interactions to omega-3 trait variance are small but non-negligible.

Our gene-based analysis revealed the G protein-coupled receptor 12 (*GPR12*) gene as an interacting locus for PUFAs% and the PUFAs:MUFAs ratio. This gene is highly expressed in the central nervous system and encodes the constitutively active G protein-coupled receptor 12 that promotes cyclic AMP production.[46] GPR12 is an orphan receptor, meaning it is unconfirmed what endogenous ligand it binds to.[46] Regarding PUFAs, it is unclear how the GPR12 receptor is associated with them. However, the GPR12 receptor is phylogenetically related to cannabinoid receptors,

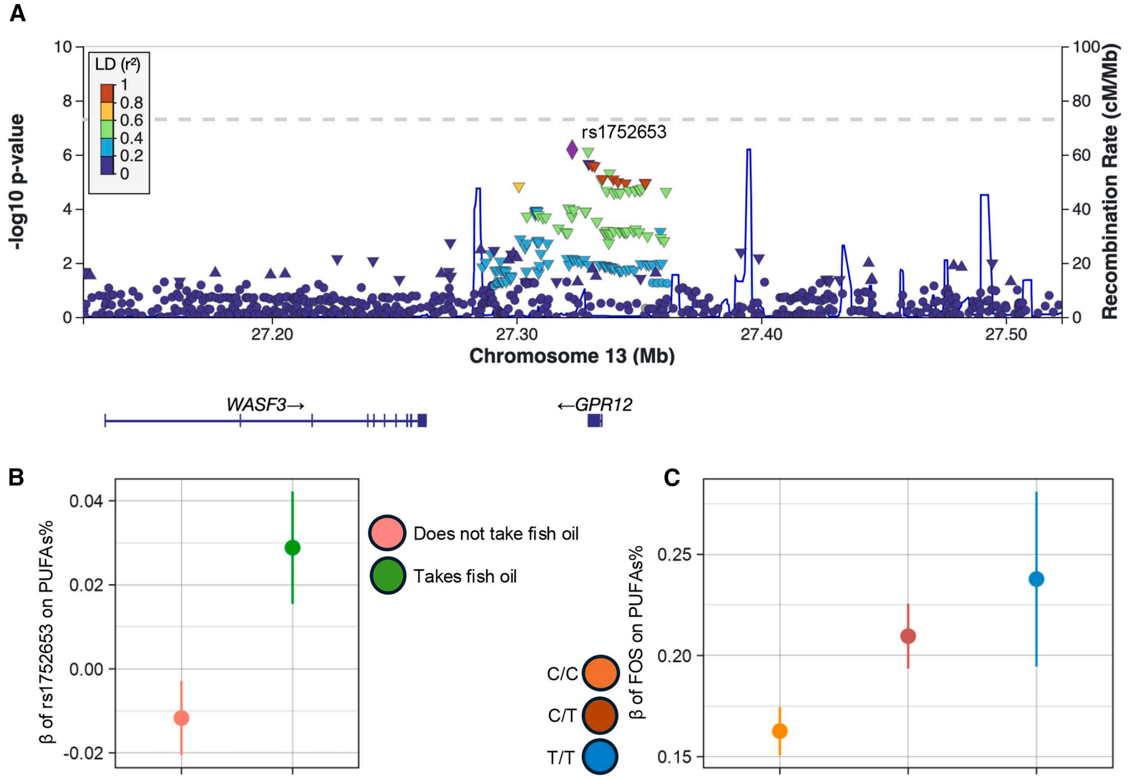

Figure 3. ***GPR12*** **variant with suggestive genome-wide significance ($p < 5.0 \times 10^{-6}$) in PUFAs%**
(A) LocusZoom plot of the *GPR12* locus for PUFAs%.
(B) Marginal genetic effect of rs1752653 on PUFAs% in stratified exposure subgroups.
(C) Marginal exposure effects of FOS on PUFAs% in stratified genotype subgroups.
All error bars represent 95% confidence intervals.

which bind to certain endocannabinoids, including omega-6-derived endocannabinoids.[47,48] Recent evidence shows that omega-3-derived endocannabinoids also exist.[47,48] Future research to experimentally establish a connection between PUFAs-derived endocannabinoids and the GPR12 receptor will support our findings.

A significant strength of our study is the large sample with complete dietary, genomic, and NMR PUFA information. We also performed our analyses at both the variant and gene levels and estimated the contributions of genome-wide gene-FOS interactions to the phenotypic variance of circulating PUFA traits, providing the most extensive analysis of gene-FOS interactions. However, our study has several limitations. First, we restricted this study to European-ancestry participants and did not have large-enough datasets to replicate these results in other populations. Large-enough datasets with the necessary exposure, genomic, and phenotypic information to detect interactions are still uncommon. Second, for FOS information, we relied on only one dietary questionnaire administered to participants at one time point. This questionnaire did not provide estimates of the frequency of FOS intake, such as daily or weekly. It also did not provide information on the types and compositions of FOS. Hence, we could not account for dosage heterogeneity in our model. Third, other dietary sources of omega-6 and omega-3 PUFAs, such as vegetable oils and fatty fish, can alter circulating PUFA levels.[5,6,44] Notably, our sensitivity analyses accounting for oily fish intake or estimated omega-3 intake indicated the robustness of our genome-wide significant findings. Furthermore, FOS status is correlated with other lifestyle and socioeconomic factors.[49] There is a possibility that the significant interactions we observed in our results were instead driven by other exposures that we did not explore in this study. However, our study had enough statistical power and was rigorous enough to replicate the *FADS* locus, giving our findings credibility.

Conclusion

Our study revealed a locus that significantly alters the effect of FOS on PUFAs- and MUFAs-related traits. Taking genetic information into account when making nutritional recommendations is the hallmark of precision nutrition, which aims to move away from the one-size-fits-all dietary recommendation model. Variants that influence the effects of dietary PUFAs on plasma PUFAs will help inform diet-based disease prevention and treatment strategies. Additionally, accounting for gene-FOS interactions will aid in developing more accurate polygenic scores for PUFAs-related traits or PUFAs-associated diseases. Lastly, our study also brings to light a potentially unexplored association between the *GPR12* gene and PUFAs. A future research direction to

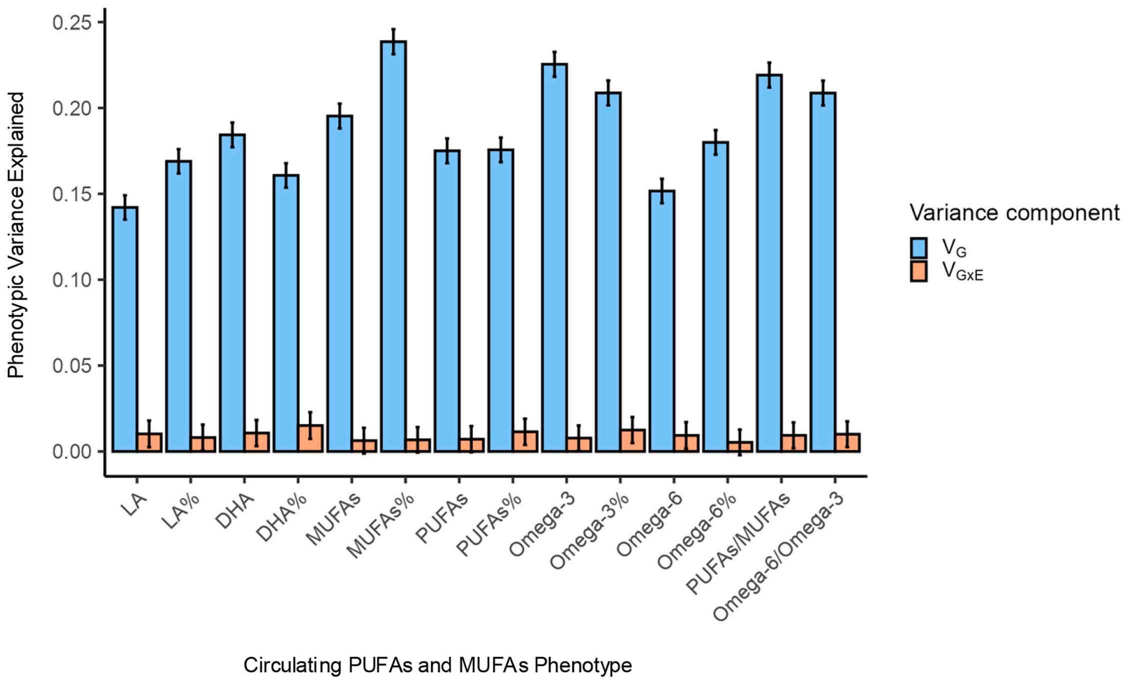

Figure 4. The proportion of phenotypic variance explained by genetic factors (V_G) and genome-wide gene-FOS interactions ($V_{G\times E}$) in the combined dataset
Error bars show 95% confidence intervals.

experimentally validate this association may highlight PUFA supplementation as a potential treatment for *GPR12*-related diseases, such as schizophrenia.[50]

Data and code availability

The accession numbers for the summary statistics generated in this study are GWAS Catalog: GCST90565570, GCST90565571, GCST90565572, GCST90565573, GCST90565574, GCST90565575, GCST90565576, GCST90565577, GCST90565578, GCST90565579, GCST90565580, GCST90565581, GCST90565582, GCST90565583 (https://www.ebi.ac.uk/gwas/). Scripts used to process the data, perform analysis, and interpret results are available on GitHub (https://github.com/adannasusan/PUFA-GxE).

Acknowledgments

We would like to acknowledge and express our gratitude to the UK Biobank participants and administrative staff. Research reported in this publication was supported by the National Institute of General Medical Sciences of the National Institutes of Health under award no. R35GM143060 (to K.Y.). The content is solely the authors' responsibility and does not necessarily represent the official views of the National Institutes of Health.

Declaration of interests

The authors declare no competing interests.

Supplemental information

Supplemental information can be found online at https://doi.org/10.1016/j.xhgg.2025.100459.

Received: December 17, 2024
Accepted: May 16, 2025

References

1. Lemaitre, R.N., Tanaka, T., Tang, W., Manichaikul, A., Foy, M., Kabagambe, E.K., Nettleton, J.A., King, I.B., Weng, L.C., Bhattacharya, S., et al. (2011). Genetic loci associated with plasma phospholipid n-3 fatty acids: a meta-analysis of genome-wide association studies from the CHARGE Consortium. PLoS Genet. *7*, e1002193. https://doi.org/10.1371/journal.pgen.1002193.
2. Guan, W., Steffen, B.T., Lemaitre, R.N., Wu, J.H.Y., Tanaka, T., Manichaikul, A., Foy, M., Rich, S.S., Wang, L., Nettleton, J.A., et al. (2014). Genome-wide association study of plasma N6 polyunsaturated fatty acids within the cohorts for heart and aging research in genomic epidemiology consortium. Circ. Cardiovasc. Genet. *7*, 321–331. https://doi.org/10.1161/CIRCGENETICS.113.000208.
3. Tintle, N.L., Pottala, J.V., Lacey, S., Ramachandran, V., Westra, J., Rogers, A., Clark, J., Olthoff, B., Larson, M., Harris, W., and Shearer, G.C. (2015). A genome-wide association study of saturated, mono- and polyunsaturated red blood cell fatty acids in the Framingham Heart Offspring Study. Prostaglandins Leukot. Essent. Fatty Acids *94*, 65–72. https://doi.org/10.1016/j.plefa.2014.11.007.
4. Schuchardt, J.P., Ostermann, A.I., Stork, L., Kutzner, L., Kohrs, H., Greupner, T., Hahn, A., and Schebb, N.H. (2016). Effects of docosahexaenoic acid supplementation on PUFA levels in red blood cells and plasma. Prostaglandins Leukot. Essent. Fatty Acids *115*, 12–23. https://doi.org/10.1016/j.plefa.2016.10.005.
5. Hodge, A.M., Simpson, J.A., Gibson, R.A., Sinclair, A.J., Makrides, M., O'Dea, K., English, D.R., and Giles, G.G. (2007).

Plasma phospholipid fatty acid composition as a biomarker of habitual dietary fat intake in an ethnically diverse cohort. Nutr. Metab. Cardiovasc. Dis. *17*, 415–426. https://doi.org/10.1016/j.numecd.2006.04.005.

6. Huybrechts, I., Jacobs, I., Aglago, E.K., Yammine, S., Matta, M., Schmidt, J.A., Casagrande, C., Nicolas, G., Biessy, C., Van Puyvelde, H., et al. (2023). Associations between Fatty Acid Intakes and Plasma Phospholipid Fatty Acid Concentrations in the European Prospective Investigation into Cancer and Nutrition. Nutrients *15*, 3695. https://doi.org/10.3390/nu15173695.
7. Chilton, F.H., Murphy, R.C., Wilson, B.A., Sergeant, S., Ainsworth, H., Seeds, M.C., and Mathias, R.A. (2014). Diet-gene interactions and PUFA metabolism: a potential contributor to health disparities and human diseases. Nutrients *6*, 1993–2022. https://doi.org/10.3390/nu6051993.
8. Chilton, F.H., Dutta, R., Reynolds, L.M., Sergeant, S., Mathias, R.A., and Seeds, M.C. (2017). Precision Nutrition and Omega-3 Polyunsaturated Fatty Acids: A Case for Personalized Supplementation Approaches for the Prevention and Management of Human Diseases. Nutrients *9*, 1165. https://doi.org/10.3390/nu9111165.
9. Tapiero, H., Ba, G.N., Couvreur, P., and Tew, K.D. (2002). Polyunsaturated fatty acids (PUFA) and eicosanoids in human health and pathologies. Biomed. Pharmacother. *56*, 215–222. https://doi.org/10.1016/s0753-3322(02)00193-2.
10. Saravanan, P., Davidson, N.C., Schmidt, E.B., and Calder, P. C. (2010). Cardiovascular effects of marine omega-3 fatty acids. Lancet *376*, 540–550. https://doi.org/10.1016/S0140-6736(10)60445-X.
11. Harris, W.S., Pottala, J.V., Sands, S.A., and Jones, P.G. (2007). Comparison of the effects of fish and fish-oil capsules on the n 3 fatty acid content of blood cells and plasma phospholipids. Am. J. Clin. Nutr. *86*, 1621–1625. https://doi.org/10.1093/ajcn/86.5.1621.
12. Juan, J., Huang, H., Jiang, X., Ardisson Korat, A.V., Song, M., Sun, Q., Willett, W.C., Jensen, M.K., and Kraft, P. (2018). Joint effects of fatty acid desaturase 1 polymorphisms and dietary polyunsaturated fatty acid intake on circulating fatty acid proportions. Am. J. Clin. Nutr. *107*, 826–833. https://doi.org/10.1093/ajcn/nqy025.
13. Sergeant, S., Hallmark, B., Mathias, R.A., Mustin, T.L., Ivester, P., Bohannon, M.L., Ruczinski, I., Johnstone, L., Seeds, M.C., and Chilton, F.H. (2020). Prospective clinical trial examining the impact of genetic variation in FADS1 on the metabolism of linoleic acid- and γ-linolenic acid-containing botanical oils. Am. J. Clin. Nutr. *111*, 1068–1078. https://doi.org/10.1093/ajcn/nqaa023.
14. Saleh, R.N.M., West, A.L., Ostermann, A.I., Schebb, N.H., Calder, P.C., and Minihane, A.M. (2021). APOE Genotype Modifies the Plasma Oxylipin Response to Omega-3 Polyunsaturated Fatty Acid Supplementation in Healthy Individuals. Front. Nutr. *8*, 723813. https://doi.org/10.3389/fnut.2021.723813.
15. Tomaszewski, N., He, X., Solomon, V., Lee, M., Mack, W.J., Quinn, J.F., Braskie, M.N., and Yassine, H.N. (2020). Effect of APOE Genotype on Plasma Docosahexaenoic Acid (DHA), Eicosapentaenoic Acid, Arachidonic Acid, and Hippocampal Volume in the Alzheimer's Disease Cooperative Study-Sponsored DHA Clinical Trial. J. Alzheimers Dis. *74*, 975–990. https://doi.org/10.3233/JAD-191017.
16. Yassine, H.N., Rawat, V., Mack, W.J., Quinn, J.F., Yurko-Mauro, K., Bailey-Hall, E., Aisen, P.S., Chui, H.C., and Schneider, L.S. (2016). The effect of APOE genotype on the delivery of DHA to cerebrospinal fluid in Alzheimer's disease. Alzheimers Res. Ther. *8*, 25. https://doi.org/10.1186/s13195-016-0194-x.
17. Stephensen, C.B., Armstrong, P., Newman, J.W., Pedersen, T. L., Legault, J., Schuster, G.U., Kelley, D., Vikman, S., Hartiala, J., Nassir, R., et al. (2011). ALOX5 gene variants affect eicosanoid production and response to fish oil supplementation. J. Lipid Res. *52*, 991–1003. https://doi.org/10.1194/jlr.P012864.
18. Armstrong, P., Kelley, D.S., Newman, J.W., Staggers, F.E., Sr., Hartiala, J., Allayee, H., and Stephensen, C.B. (2012). Arachidonate 5-lipoxygenase gene variants affect response to fish oil supplementation by healthy African Americans. J. Nutr. *142*, 1417–1428. https://doi.org/10.3945/jn.112.159814.
19. Veenstra, J., Kalsbeek, A., Westra, J., Disselkoen, C., Smith, C., and Tintle, N. (2017). Genome-Wide Interaction Study of Omega-3 PUFAs and Other Fatty Acids on Inflammatory Biomarkers of Cardiovascular Health in the Framingham Heart Study. Nutrients *9*, 900. https://doi.org/10.3390/nu9080900.
20. Ralston, J.C., Matravadia, S., Gaudio, N., Holloway, G.P., and Mutch, D.M. (2015). Polyunsaturated fatty acid regulation of adipocyte FADS1 and FADS2 expression and function. Obesity *23*, 725–728. https://doi.org/10.1002/oby.21035.
21. Merino, D.M., Ma, D.W.L., and Mutch, D.M. (2010). Genetic variation in lipid desaturases and its impact on the development of human disease. Lipids Health Dis. *9*, 63. https://doi.org/10.1186/1476-511X-9-63.
22. Madden, J., Williams, C.M., Calder, P.C., Lietz, G., Miles, E. A., Cordell, H., Mathers, J.C., and Minihane, A.M. (2011). The impact of common gene variants on the response of biomarkers of cardiovascular disease (CVD) risk to increased fish oil fatty acids intakes. Annu. Rev. Nutr. *31*, 203–234. https://doi.org/10.1146/annurev-nutr-010411-095239.
23. Schulze, M.B., Minihane, A.M., Saleh, R.N.M., and Risérus, U. (2020). Intake and metabolism of omega-3 and omega-6 polyunsaturated fatty acids: nutritional implications for cardiometabolic diseases. Lancet Diabetes Endocrinol. *8*, 915–930. https://doi.org/10.1016/S2213-8587(20)30148-0.
24. Huang, L., Zhang, F., Xu, P., Zhou, Y., Liu, Y., Zhang, H., Tan, X., Ge, X., Xu, Y., Guo, M., and Long, Y. (2023). Effect of Omega-3 Polyunsaturated Fatty Acids on Cardiovascular Outcomes in Patients with Diabetes: A Meta-analysis of Randomized Controlled Trials. Adv. Nutr. *14*, 629–636. https://doi.org/10.1016/j.advnut.2023.04.009.
25. Wu, S., Ding, Y., Wu, F., Li, R., Hou, J., and Mao, P. (2015). Omega-3 fatty acids intake and risks of dementia and Alzheimer's disease: a meta-analysis. Neurosci. Biobehav. Rev. *48*, 1–9. https://doi.org/10.1016/j.neubiorev.2014.11.008.
26. Kavyani, Z., Musazadeh, V., Fathi, S., Hossein Faghfouri, A., Dehghan, P., and Sarmadi, B. (2022). Efficacy of the omega-3 fatty acids supplementation on inflammatory biomarkers: An umbrella meta-analysis. Int. Immunopharmacol. *111*, 109104. https://doi.org/10.1016/j.intimp.2022.109104.
27. Wei, L., Wu, Z., and Chen, Y.Q. (2022). Multi-targeted therapy of cancer by omega-3 fatty acids-an update. Cancer Lett. *526*, 193–204. https://doi.org/10.1016/j.canlet.2021.11.023.
28. Francis, M., Li, C., Sun, Y., Zhou, J., Li, X., Brenna, J.T., and Ye, K. (2021). Genome-wide association study of fish oil supplementation on lipid traits in 81,246 individuals reveals

new gene-diet interaction loci. PLoS Genet. *17*, e1009431. https://doi.org/10.1371/journal.pgen.1009431.
29. Bycroft, C., Freeman, C., Petkova, D., Band, G., Elliott, L.T., Sharp, K., Motyer, A., Vukcevic, D., Delaneau, O., O'Connell, J., et al. (2018). The UK Biobank resource with deep phenotyping and genomic data. Nature *562*, 203–209. https://doi.org/10.1038/s41586-018-0579-z.
30. Sudlow, C., Gallacher, J., Allen, N., Beral, V., Burton, P., Danesh, J., Downey, P., Elliott, P., Green, J., Landray, M., et al. (2015). UK biobank: an open access resource for identifying the causes of a wide range of complex diseases of middle and old age. PLoS Med. *12*, e1001779. https://doi.org/10.1371/journal.pmed.1001779.
31. Karczewski, K.J., Gupta, R., Kanai, M., Lu, W., Tsuo, K., Wang, Y., Walters, R.K., Turley, P., Callier, S., Shah, N.N., et al. (2024). Pan-UK Biobank GWAS improves discovery, analysis of genetic architecture, and resolution into ancestry-enriched effects. Preprint at medRxiv. 2024.2003.2013.24303864. https://doi.org/10.1101/2024.03.13.24303864.
32. Chang, C.C., Chow, C.C., Tellier, L.C., Vattikuti, S., Purcell, S.M., and Lee, J.J. (2015). Second-generation PLINK: rising to the challenge of larger and richer datasets. GigaScience *4*, 7. https://doi.org/10.1186/s13742-015-0047-8.
33. Westerman, K.E., Pham, D.T., Hong, L., Chen, Y., Sevilla-González, M., Sung, Y.J., Sun, Y.V., Morrison, A.C., Chen, H., and Manning, A.K. (2021). GEM: scalable and flexible gene-environment interaction analysis in millions of samples. Bioinformatics *37*, 3514–3520. https://doi.org/10.1093/bioinformatics/btab223.
34. Wang, Y., Li, D., and Wei, P. (2015). Powerful Tukey's One Degree-of-Freedom Test for Detecting Gene-Gene and Gene-Environment Interactions. Cancer Inform. *14*, 209–218. https://doi.org/10.4137/CIN.S17305.
35. Rist, M.J., Roth, A., Frommherz, L., Weinert, C.H., Krüger, R., Merz, B., Bunzel, D., Mack, C., Egert, B., Bub, A., et al. (2017). Metabolite patterns predicting sex and age in participants of the Karlsruhe Metabolomics and Nutrition (KarMeN) study. PLoS One *12*, e0183228. https://doi.org/10.1371/journal.pone.0183228.
36. Watanabe, K., Taskesen, E., van Bochoven, A., and Posthuma, D. (2017). Functional mapping and annotation of genetic associations with FUMA. Nat. Commun. *8*, 1826. https://doi.org/10.1038/s41467-017-01261-5.
37. de Leeuw, C.A., Mooij, J.M., Heskes, T., and Posthuma, D. (2015). MAGMA: generalized gene-set analysis of GWAS data. PLoS Comput. Biol. *11*, e1004219. https://doi.org/10.1371/journal.pcbi.1004219.
38. Barbeira, A.N., Dickinson, S.P., Bonazzola, R., Zheng, J., Wheeler, H.E., Torres, J.M., Torstenson, E.S., Shah, K.P., Garcia, T., Edwards, T.L., et al. (2018). Exploring the phenotypic consequences of tissue specific gene expression variation inferred from GWAS summary statistics. Nat. Commun. *9*, 1825. https://doi.org/10.1038/s41467-018-03621-1.
39. Barbeira, A.N., Pividori, M., Zheng, J., Wheeler, H.E., Nicolae, D.L., and Im, H.K. (2019). Integrating predicted transcriptome from multiple tissues improves association detection. PLoS Genet. *15*, e1007889. https://doi.org/10.1371/journal.pgen.1007889.
40. Yang, J., Benyamin, B., McEvoy, B.P., Gordon, S., Henders, A.K., Nyholt, D.R., Madden, P.A., Heath, A.C., Martin, N.G., Montgomery, G.W., et al. (2010). Common SNPs explain a large proportion of the heritability for human height. Nat. Genet. *42*, 565–569. https://doi.org/10.1038/ng.608.
41. Takkunen, M.J., de Mello, V.D., Schwab, U.S., Kuusisto, J., Vaittinen, M., Ågren, J.J., Laakso, M., Pihlajamäki, J., and Uusitupa, M.I.J. (2016). Gene-diet interaction of a common FADS1 variant with marine polyunsaturated fatty acids for fatty acid composition in plasma and erythrocytes among men. Mol. Nutr. Food Res. *60*, 381–389. https://doi.org/10.1002/mnfr.201500594.
42. Roke, K., and Mutch, D.M. (2014). The role of FADS1/2 polymorphisms on cardiometabolic markers and fatty acid profiles in young adults consuming fish oil supplements. Nutrients *6*, 2290–2304. https://doi.org/10.3390/nu6062290.
43. Al-Hilal, M., Alsaleh, A., Maniou, Z., Lewis, F.J., Hall, W.L., Sanders, T.A.B., O'Dell, S.D.; and MARINA study team (2013). Genetic variation at the FADS1-FADS2 gene locus influences delta-5 desaturase activity and LC-PUFA proportions after fish oil supplement. J. Lipid Res. *54*, 542–551. https://doi.org/10.1194/jlr.P032276.
44. Ma, J., Folsom, A.R., Shahar, E., and Eckfeldt, J.H. (1995). Plasma fatty acid composition as an indicator of habitual dietary fat intake in middle-aged adults. The Atherosclerosis Risk in Communities (ARIC) Study Investigators. Am. J. Clin. Nutr. *62*, 564–571. https://doi.org/10.1093/ajcn/62.3.564.
45. Blasbalg, T.L., Hibbeln, J.R., Ramsden, C.E., Majchrzak, S.F., and Rawlings, R.R. (2011). Changes in consumption of omega-3 and omega-6 fatty acids in the United States during the 20th century. Am. J. Clin. Nutr. *93*, 950–962. https://doi.org/10.3945/ajcn.110.006643.
46. Brown, K.J., Laun, A.S., and Song, Z.H. (2017). Cannabidiol, a novel inverse agonist for GPR12. Biochem. Biophys. Res. Commun. *493*, 451–454. https://doi.org/10.1016/j.bbrc.2017.09.001.
47. Laun, A.S., Shrader, S.H., Brown, K.J., and Song, Z.H. (2019). GPR3, GPR6, and GPR12 as novel molecular targets: their biological functions and interaction with cannabidiol. Acta Pharmacol. Sin. *40*, 300–308. https://doi.org/10.1038/s41401-018-0031-9.
48. Cascio, M.G. (2013). PUFA-derived endocannabinoids: an overview. Proc. Nutr. Soc. *72*, 451–459. https://doi.org/10.1017/S0029665113003418.
49. Cundiff, D.K., Lanou, A.J., and Nigg, C.R. (2007). Relation of omega-3 Fatty Acid intake to other dietary factors known to reduce coronary heart disease risk. Am. J. Cardiol. *99*, 1230–1233. https://doi.org/10.1016/j.amjcard.2006.12.032.
50. Lu, Y., Hatzipantelis, C.J., Langmead, C.J., and Stewart, G.D. (2024). Molecular insights into orphan G protein-coupled receptors relevant to schizophrenia. Br. J. Pharmacol. *181*, 2095–2113. https://doi.org/10.1111/bph.16221.

HGGA, Volume *6*

Supplemental information

A multi-level gene-diet interaction analysis of fish oil and 14 polyunsaturated fatty acid traits identifies the *FADS* and *GPR12* loci

Susan Adanna Ihejirika, Alexandra Huong Chiang, Aryaman Singh, Eunice Stephen, Han Chen, and Kaixiong Ye

Table of Contents

Figure S1. Participant flowchart.. 2

Figure S2. Manhattan and QQ plots of p-values for gene-FOS interactions in 14 PUFAs and MUFAs-related phenotypes for 85,708 participants in the Phase One dataset 9

Figure S3. Scatter plots of p-values, across the genome, of gene-FOS interactions across 14 PUFAs and MUFAs-related phenotypes in the Phases One and Two datasets 13

Figure S4. Manhattan and QQ plots of p-values for gene-fish oil interactions in 14 PUFAs and MUFAs-related phenotypes for 114,352 participants in the Phase Two dataset...20

Figure S5. Manhattan and QQ plots of p-values for gene-fish oil interactions in 14 PUFAs and MUFAs-related phenotypes for 200,060 participants in the combined dataset...27

Table S1. Baseline characteristics of participants in Phase One, Phase Two, and combined releases of NMR metabolite data of individuals of European ancestry in UK Biobank...28

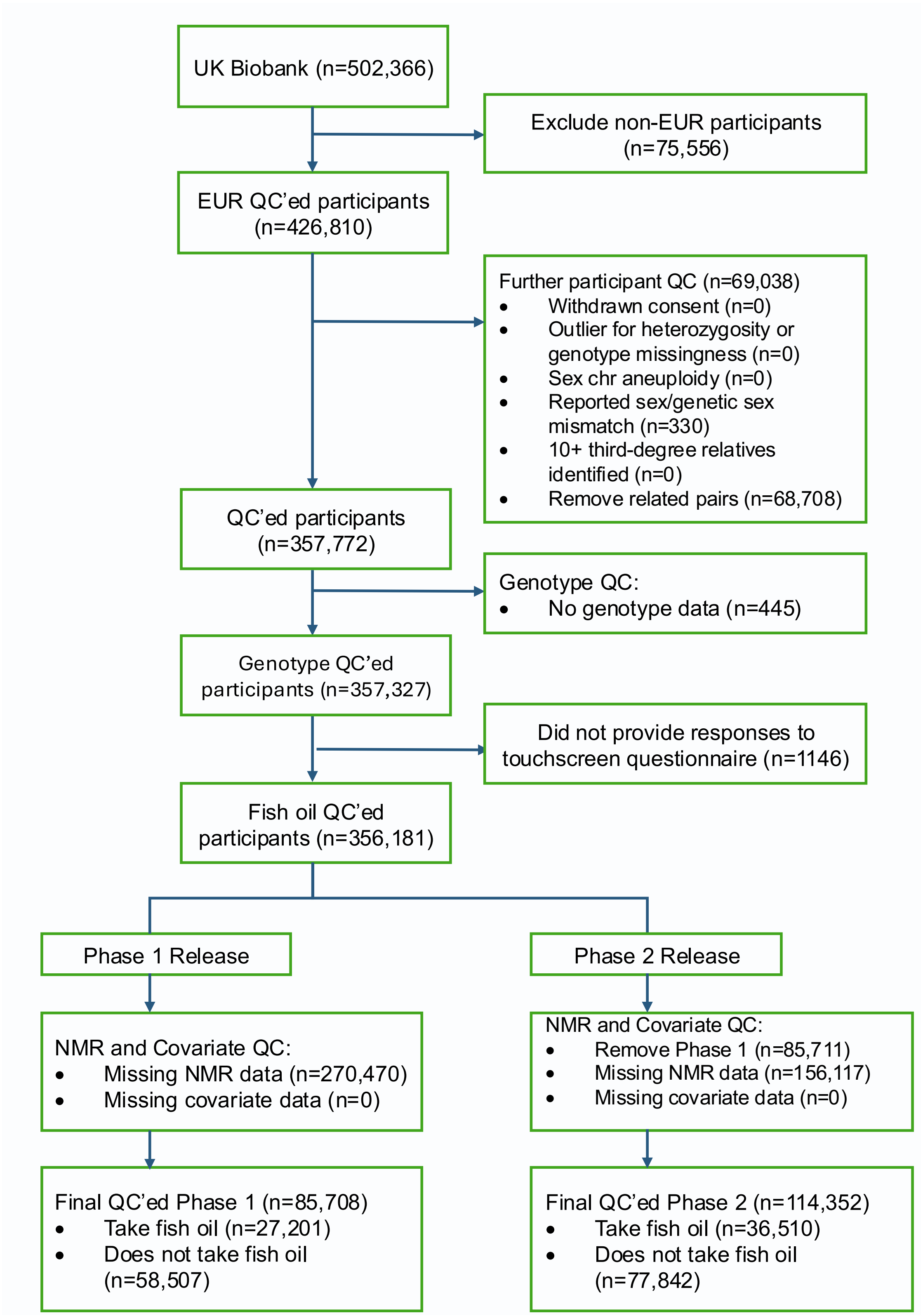

Figure S1. Participant flowchart

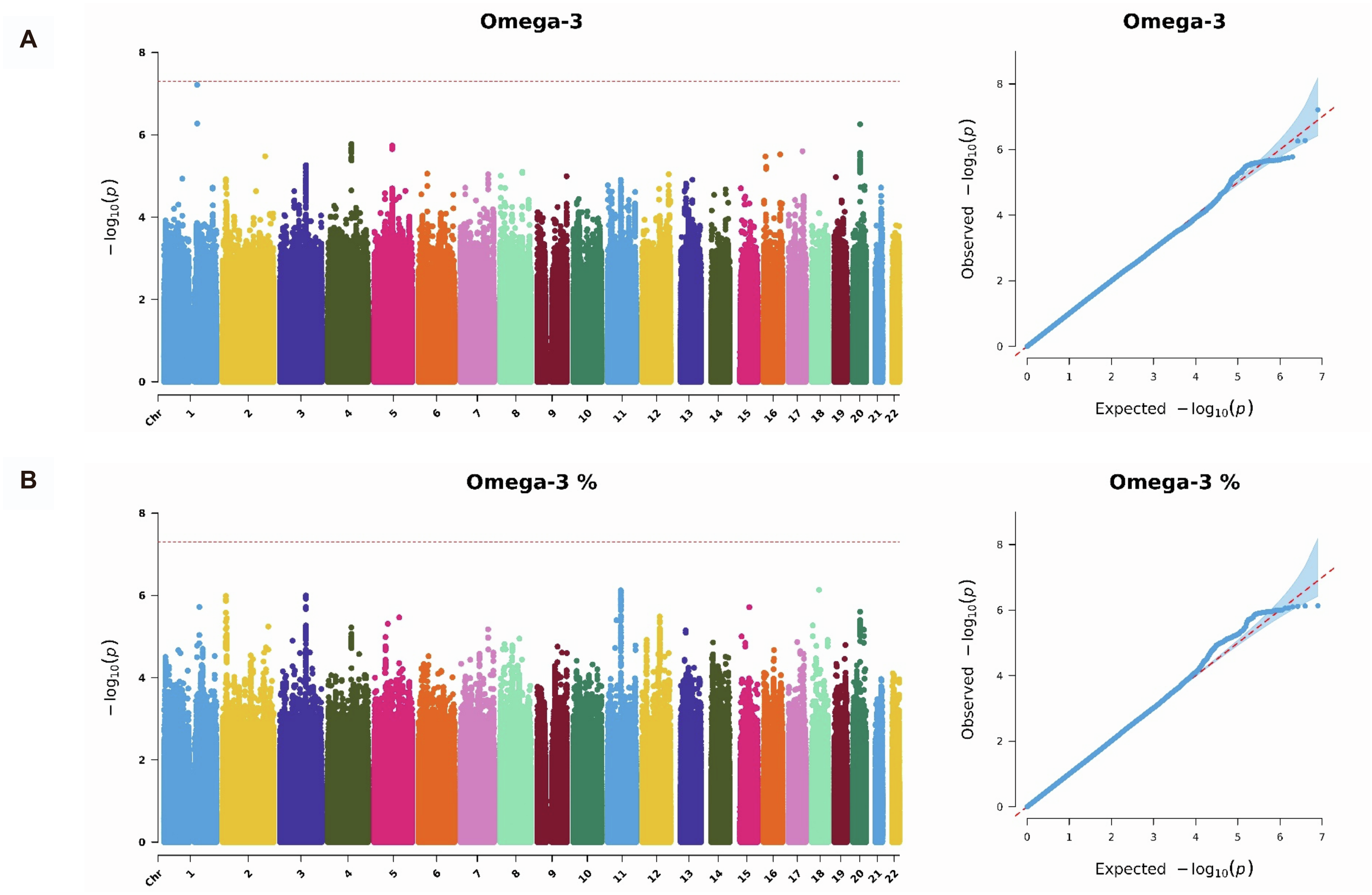
A
Omega-3
$-\log_{10}(p)$
Chr
Omega-3
Observed $-\log_{10}(p)$
Expected $-\log_{10}(p)$
B
Omega-3 %
$-\log_{10}(p)$
Chr
Omega-3 %
Observed $-\log_{10}(p)$
Expected $-\log_{10}(p)$

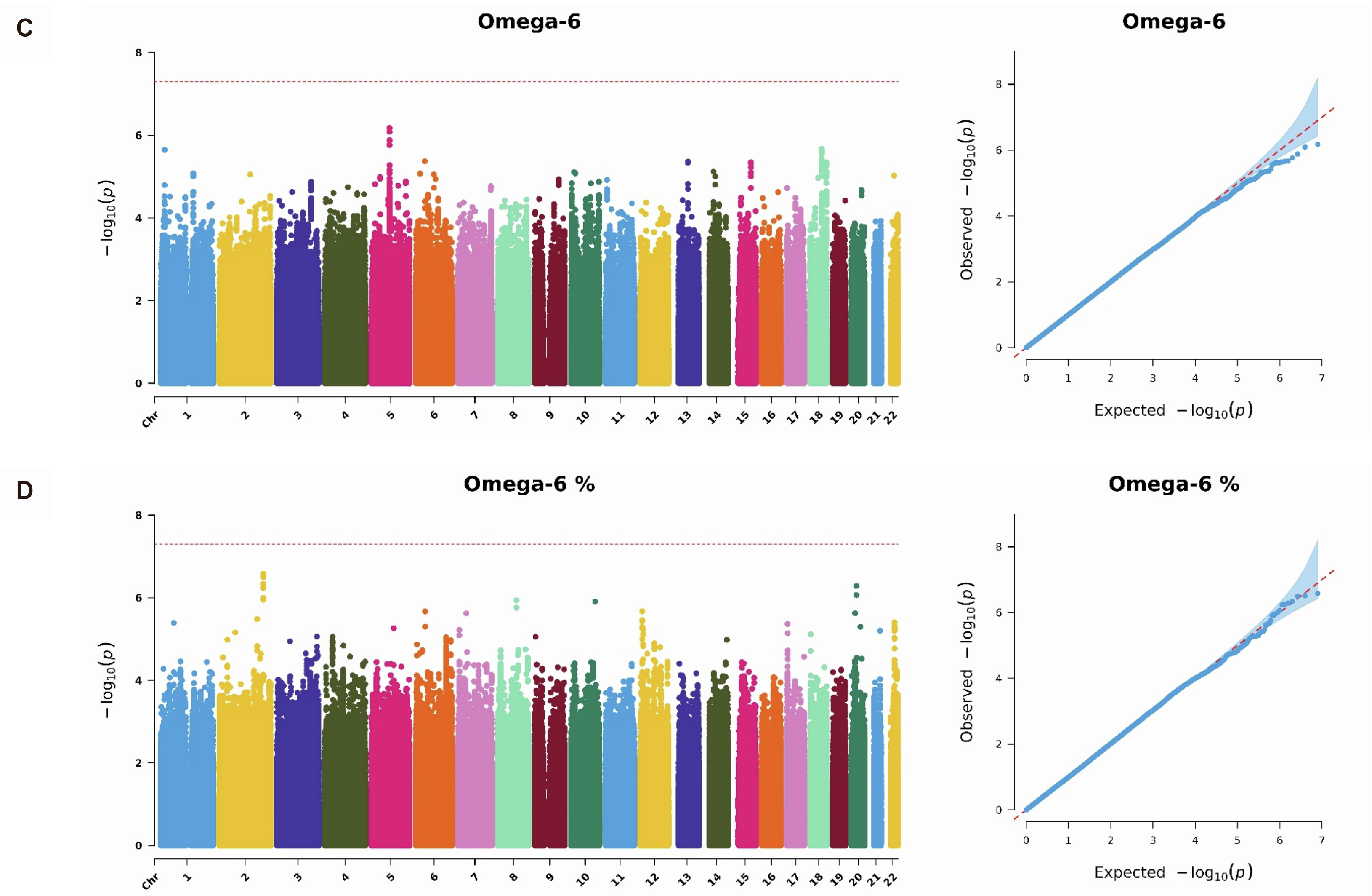
C
Omega-6
Omega-6
$-\log_{10}(p)$
Chr
Observed $-\log_{10}(p)$
Expected $-\log_{10}(p)$
D
Omega-6 %
Omega-6 %
$-\log_{10}(p)$
Chr
Observed $-\log_{10}(p)$
Expected $-\log_{10}(p)$

E

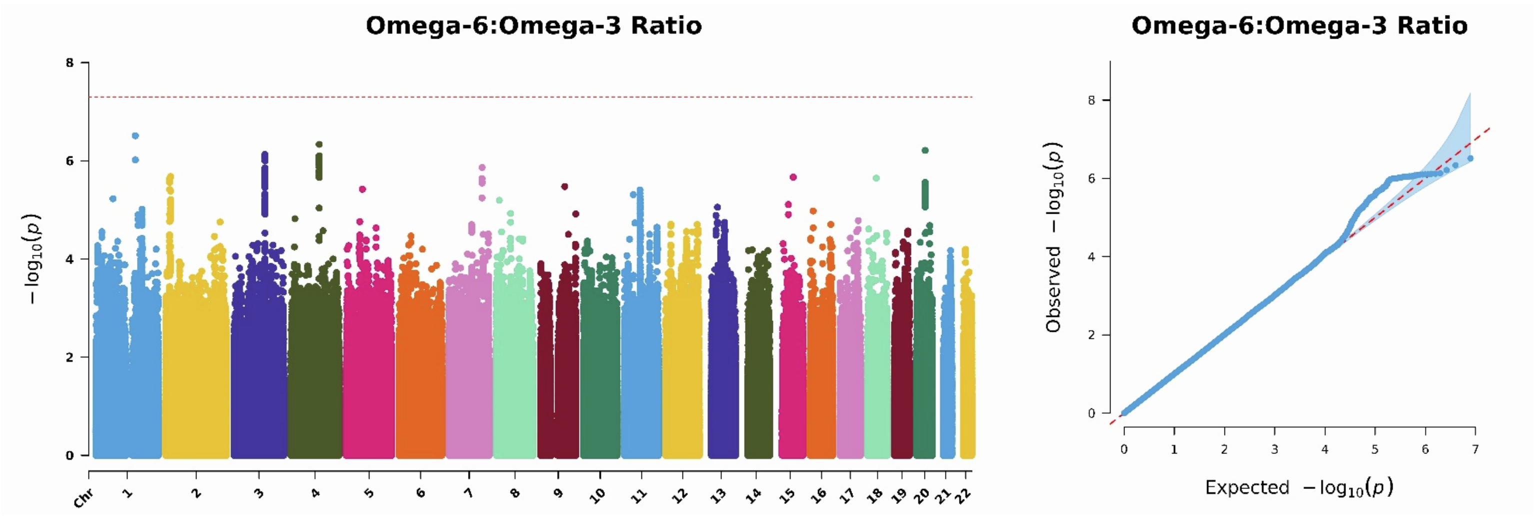

Omega-6:Omega-3 Ratio
Omega-6:Omega-3 Ratio
$-\log_{10}(p)$
Chr
Observed $-\log_{10}(p)$
Expected $-\log_{10}(p)$

F

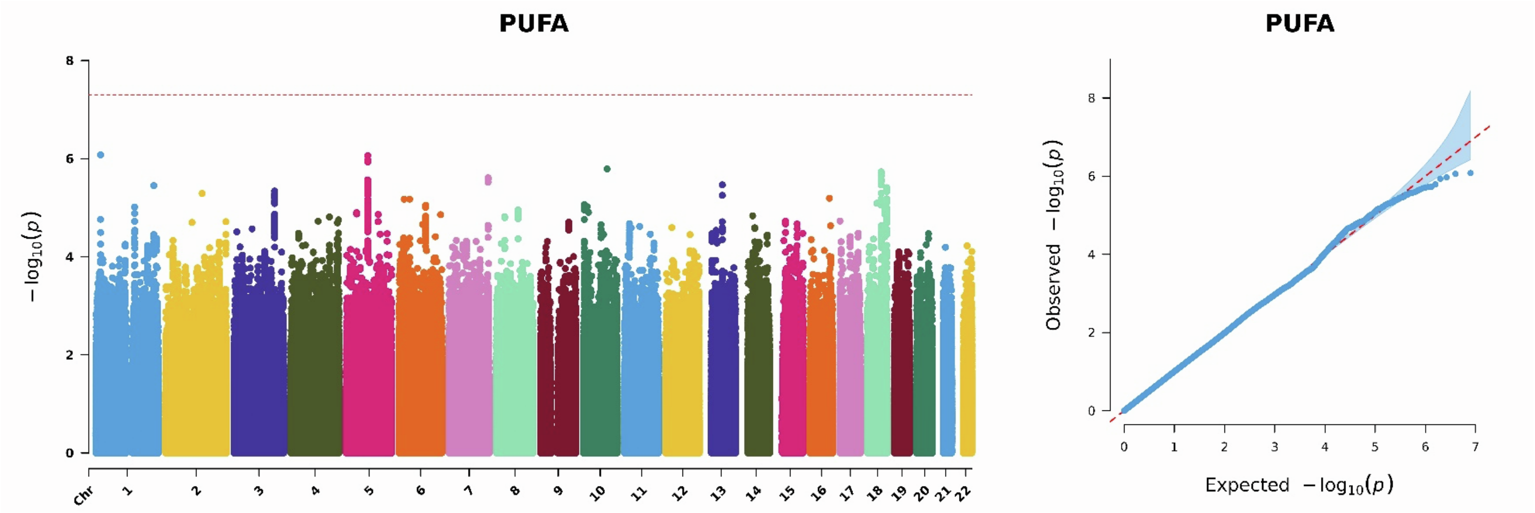

PUFA
PUFA
$-\log_{10}(p)$
Chr
Observed $-\log_{10}(p)$
Expected $-\log_{10}(p)$

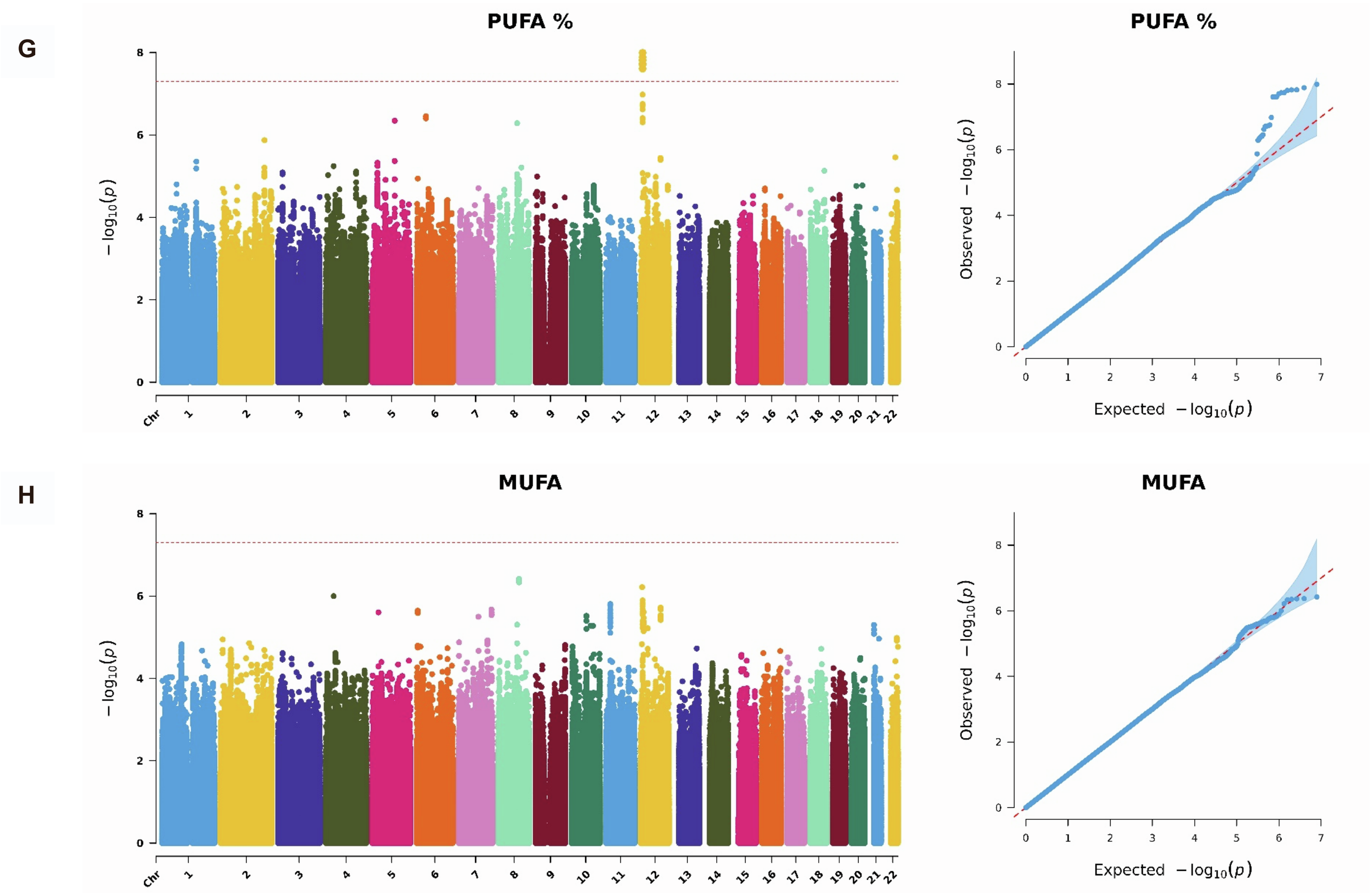

G
PUFA %
−log10(p)
Chr
Observed −log10(p)
Expected −log10(p)
H
MUFA
−log10(p)
Chr
Observed −log10(p)
Expected −log10(p)

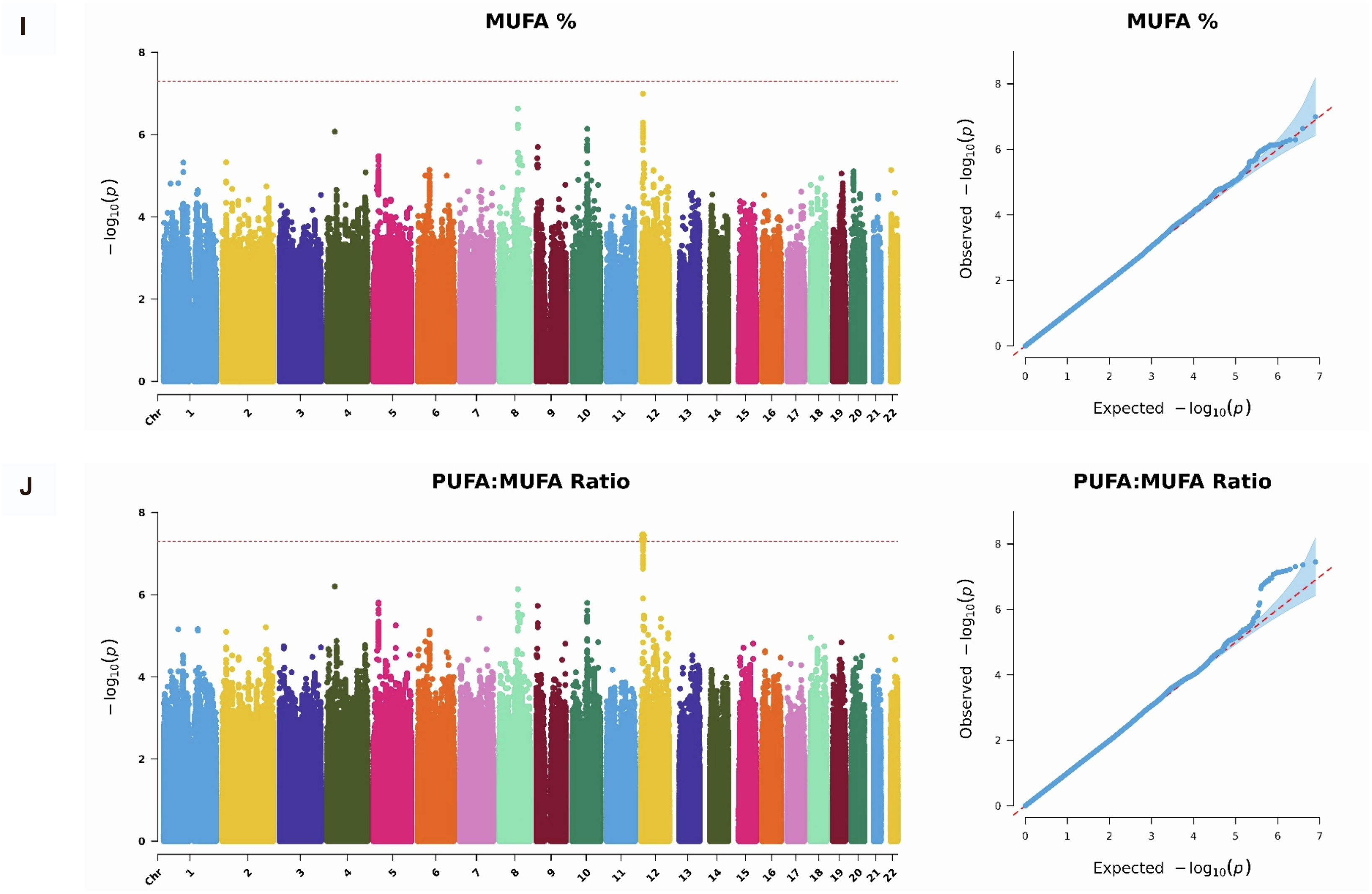
I
MUFA %
− log10(p)
Chr
1
2
3
4
5
6
7
8
9
10
11
12
13
14
15
16
17
18
19
20
21
22
MUFA %
Observed − log10(p)
Expected − log10(p)
J
PUFA:MUFA Ratio
− log10(p)
Chr
PUFA:MUFA Ratio
Observed − log10(p)
Expected − log10(p)

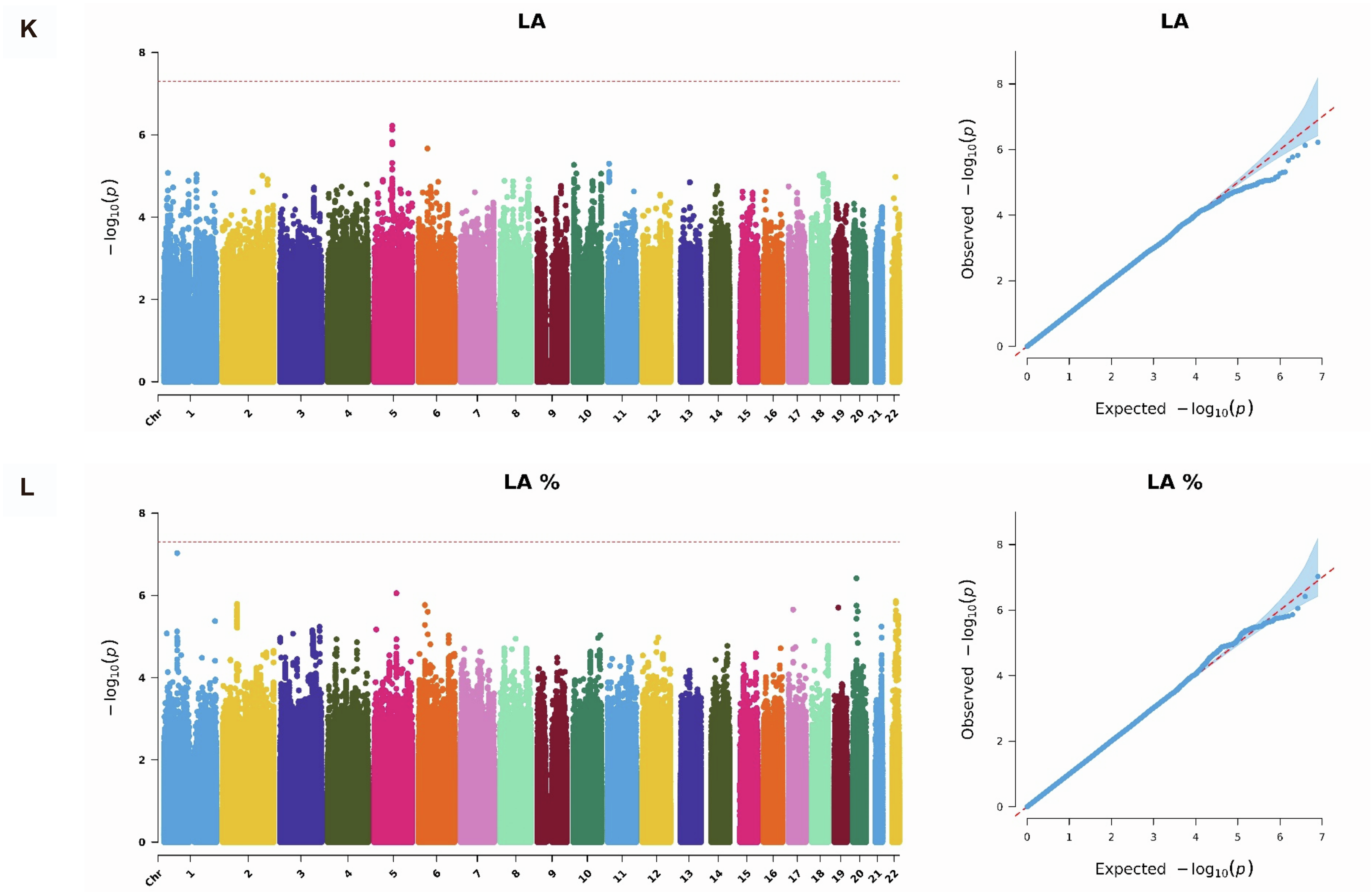
K
LA
LA
L
LA %
LA %
Chr
Expected −log10(p)
Observed −log10(p)
−log10(p)

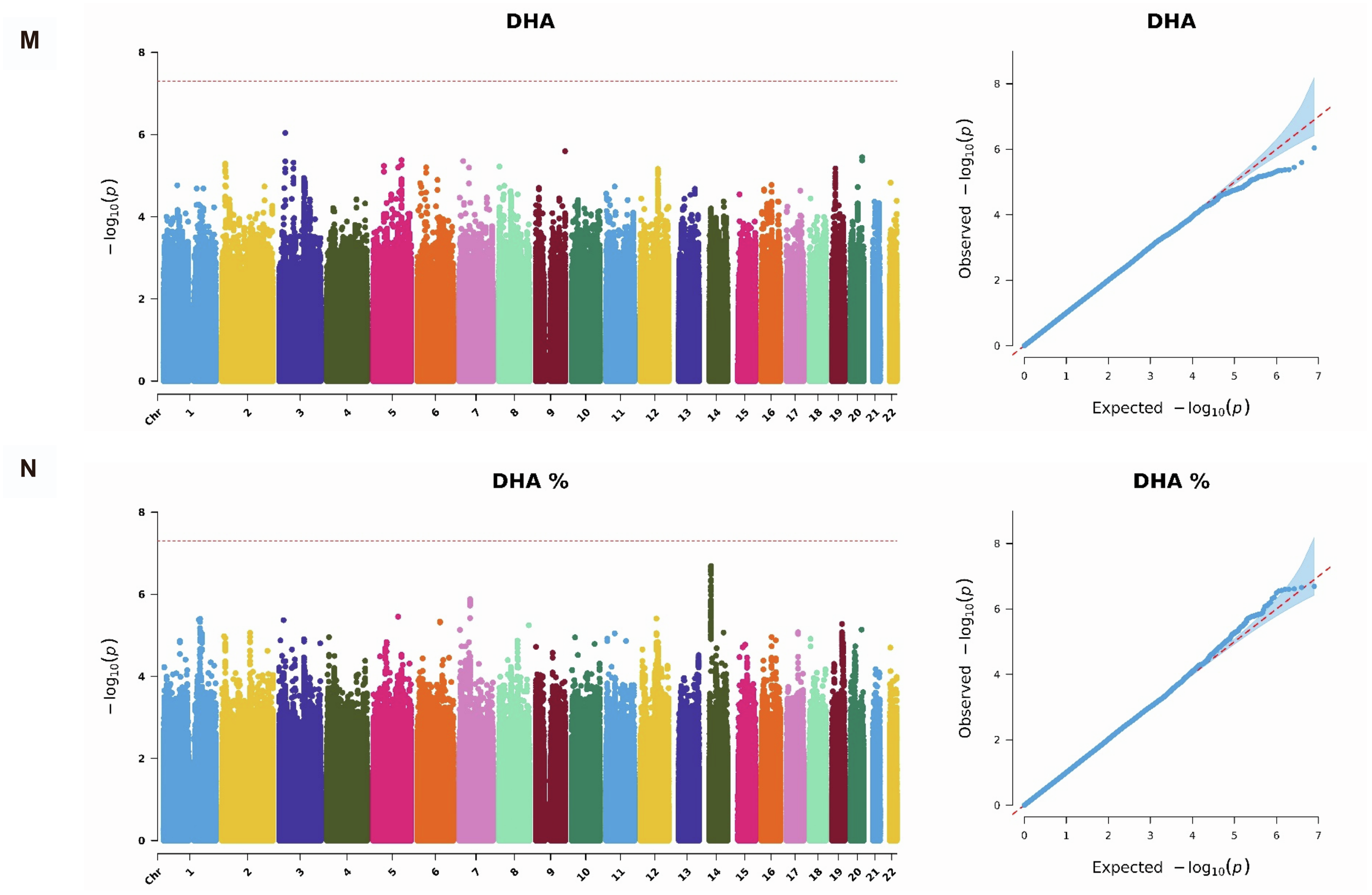

Figure S2. Manhattan and QQ plots of *p*-values for gene-FOS interactions in 14 PUFAs and MUFAs-related phenotypes for 85,708 participants in the Phase One dataset

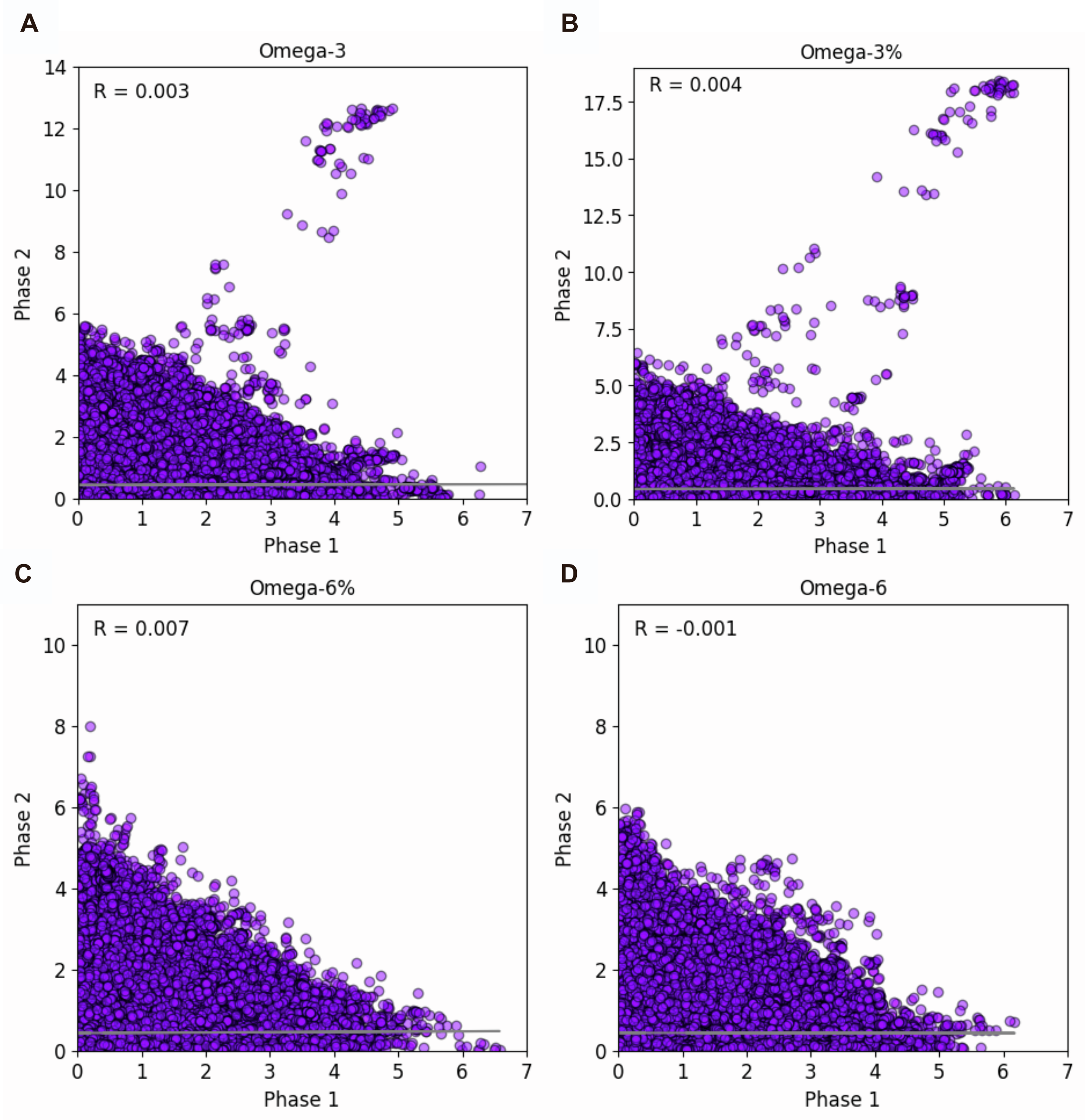

A
Omega-3
R = 0.003
Phase 2
Phase 1
B
Omega-3%
R = 0.004
Phase 2
Phase 1
C
Omega-6%
R = 0.007
Phase 2
Phase 1
D
Omega-6
R = -0.001
Phase 2
Phase 1

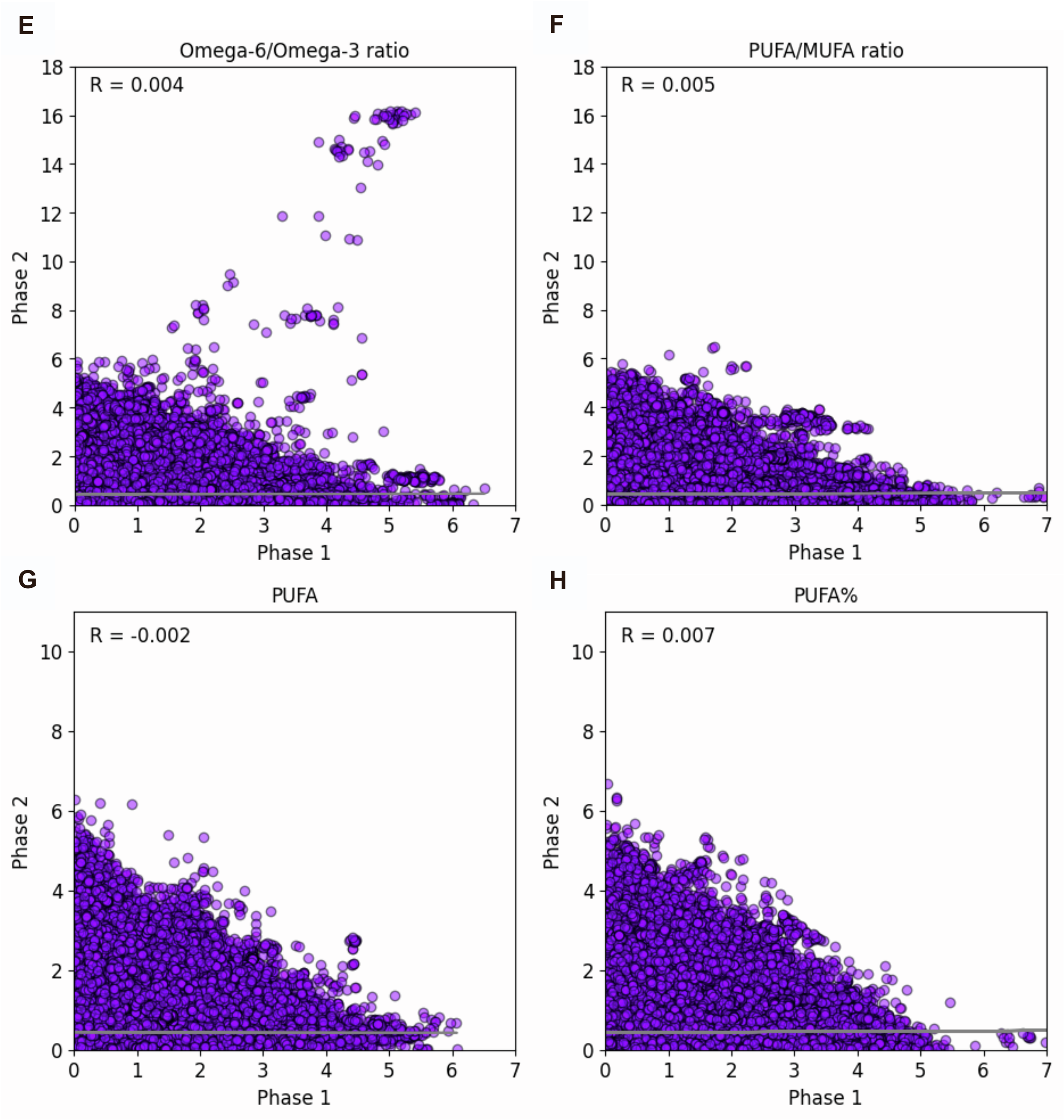
E
Omega-6/Omega-3 ratio
R = 0.004
Phase 2
Phase 1
F
PUFA/MUFA ratio
R = 0.005
Phase 2
Phase 1
G
PUFA
R = -0.002
Phase 2
Phase 1
H
PUFA%
R = 0.007
Phase 2
Phase 1

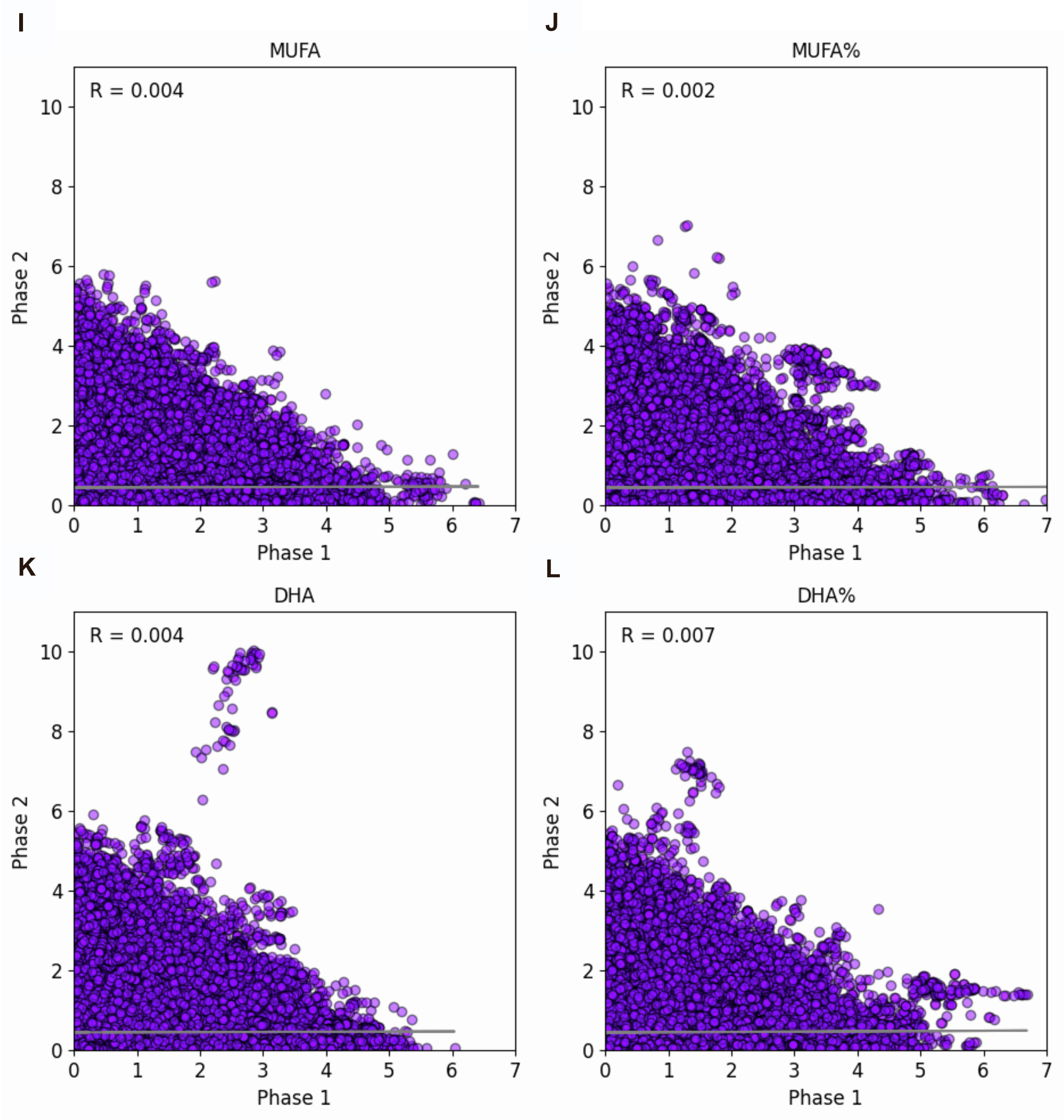

I
MUFA
R = 0.004
Phase 2
Phase 1
J
MUFA%
R = 0.002
Phase 2
Phase 1
K
DHA
R = 0.004
Phase 2
Phase 1
L
DHA%
R = 0.007
Phase 2
Phase 1

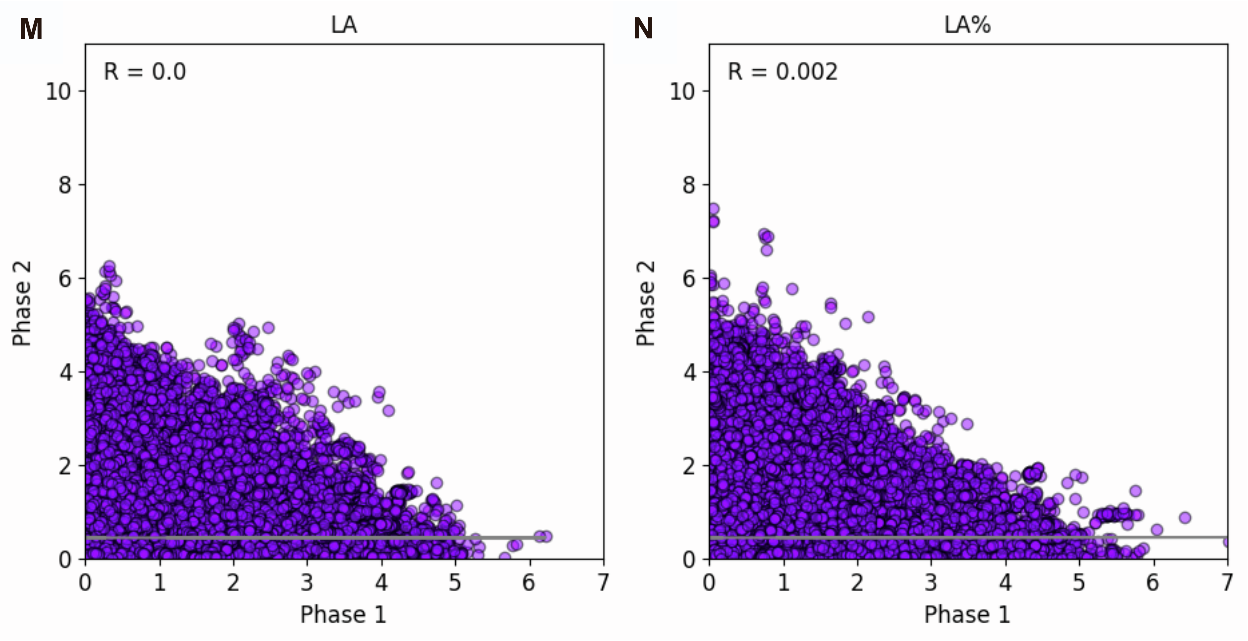

Figure S3. Scatter plots of *p*-values, across the genome, of gene-FOS interactions across 14 PUFAs and MUFAs-related phenotypes in the Phases One and Two datasets

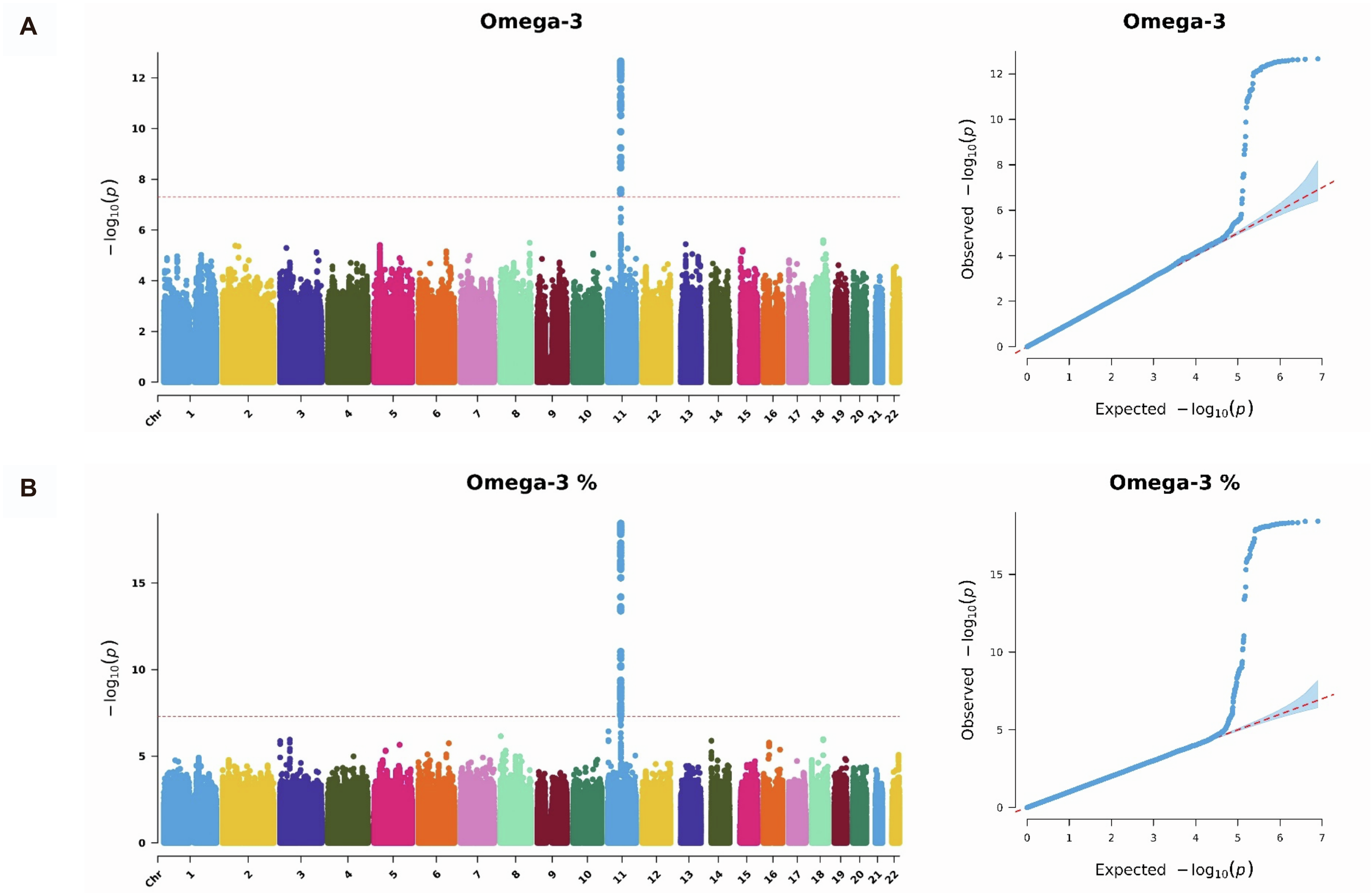
A
Omega-3
Omega-3
−log10(p)
Chr 1 2 3 4 5 6 7 8 9 10 11 12 13 14 15 16 17 18 19 20 21 22
Observed −log10(p)
Expected −log10(p)
B
Omega-3 %
Omega-3 %
−log10(p)
Chr 1 2 3 4 5 6 7 8 9 10 11 12 13 14 15 16 17 18 19 20 21 22
Observed −log10(p)
Expected −log10(p)

C

D

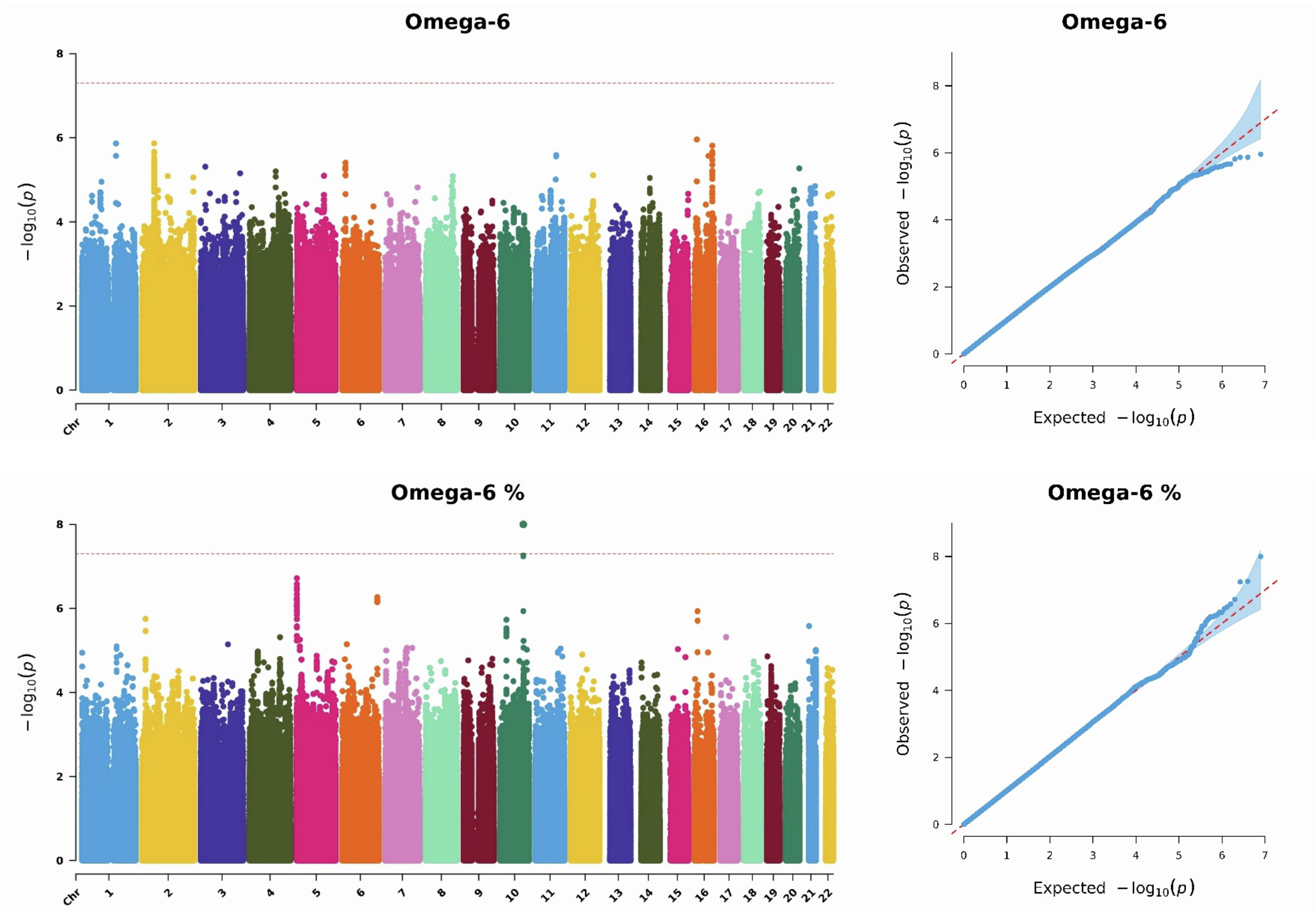

Omega-6
Omega-6
Omega-6 %
Omega-6 %
$-\log_{10}(p)$
Observed $-\log_{10}(p)$
Expected $-\log_{10}(p)$
Chr

E

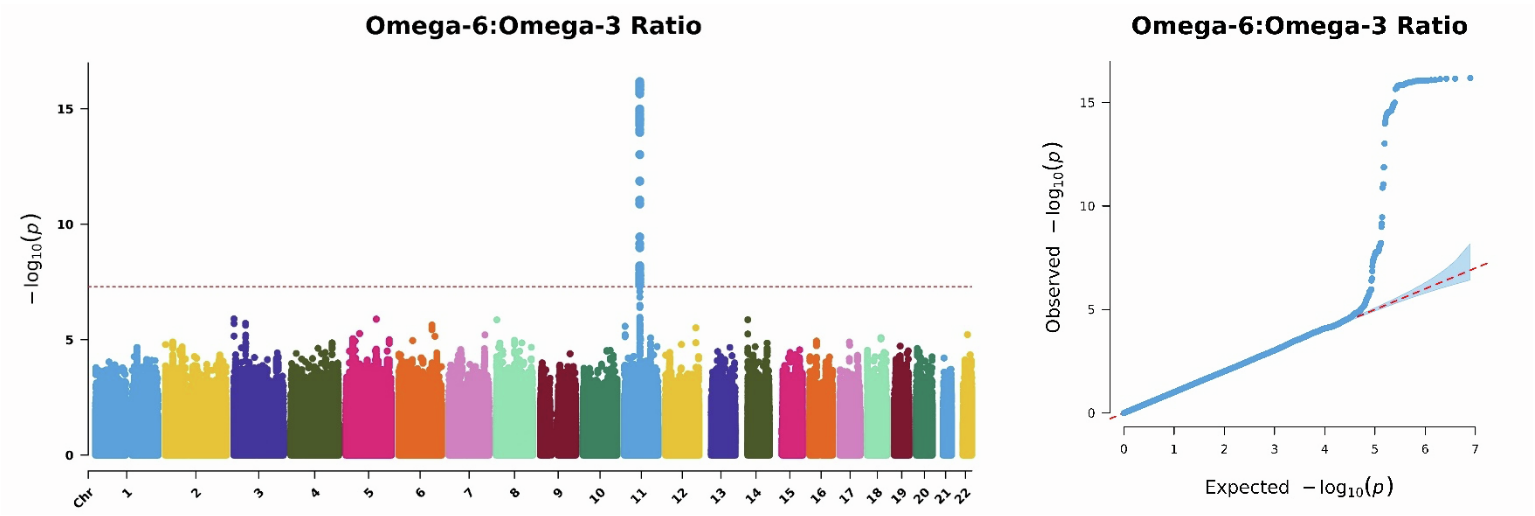
Omega-6:Omega-3 Ratio
$-\log_{10}(p)$
Chr
Omega-6:Omega-3 Ratio
Observed $-\log_{10}(p)$
Expected $-\log_{10}(p)$

F

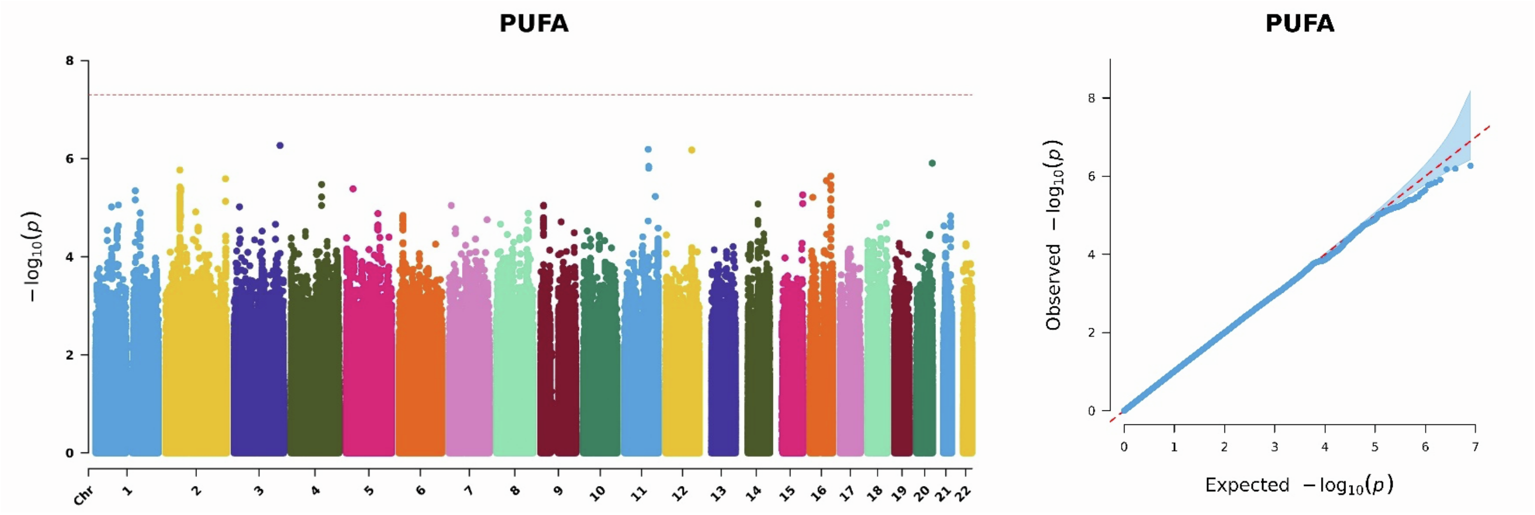
PUFA
$-\log_{10}(p)$
Chr
PUFA
Observed $-\log_{10}(p)$
Expected $-\log_{10}(p)$

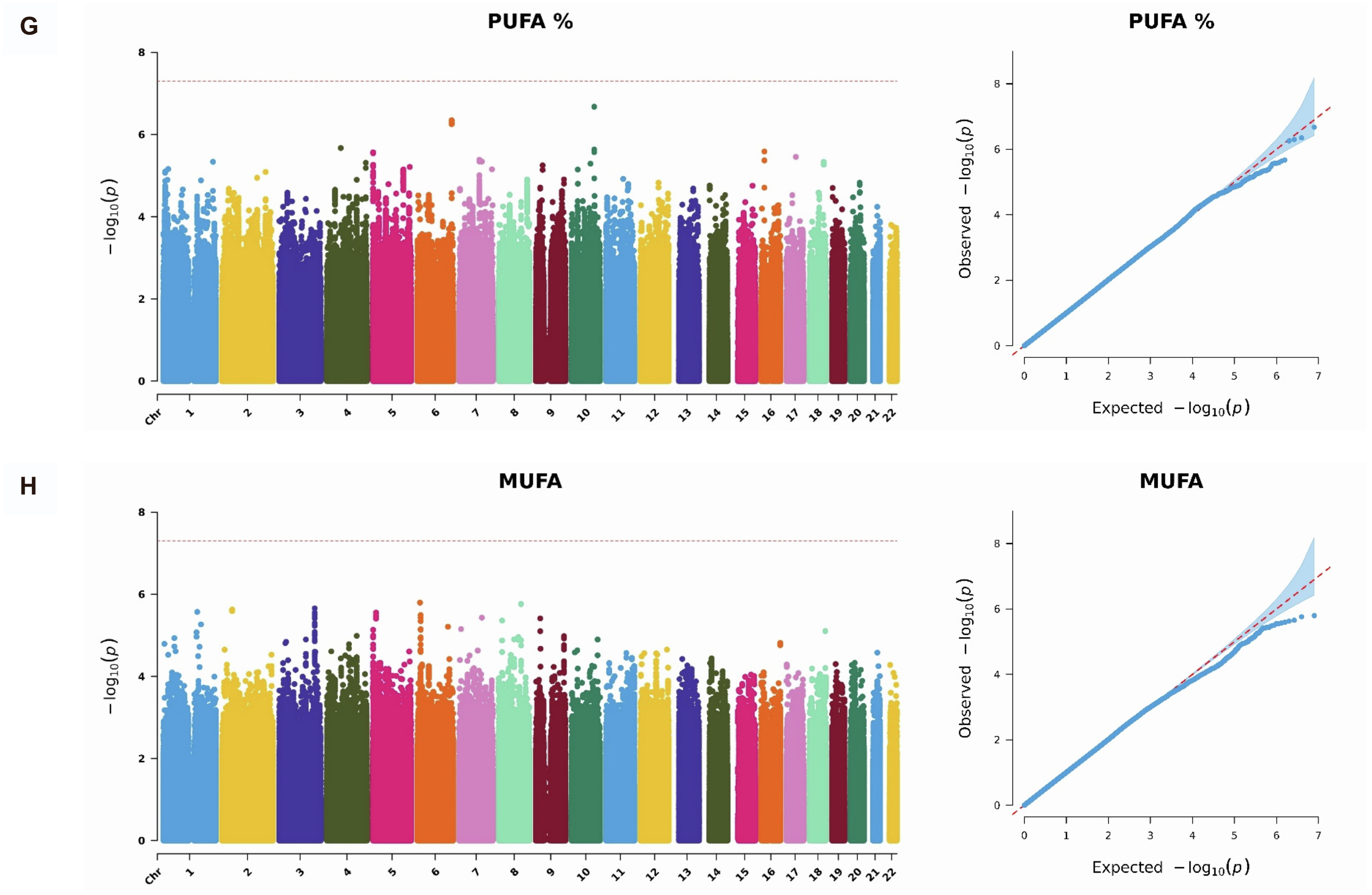
G
PUFA %
$-\log_{10}(p)$
Chr
1
2
3
4
5
6
7
8
9
10
11
12
13
14
15
16
17
18
19
20
21
22
PUFA %
Observed $-\log_{10}(p)$
Expected $-\log_{10}(p)$
H
MUFA
$-\log_{10}(p)$
MUFA
Observed $-\log_{10}(p)$
Expected $-\log_{10}(p)$

I

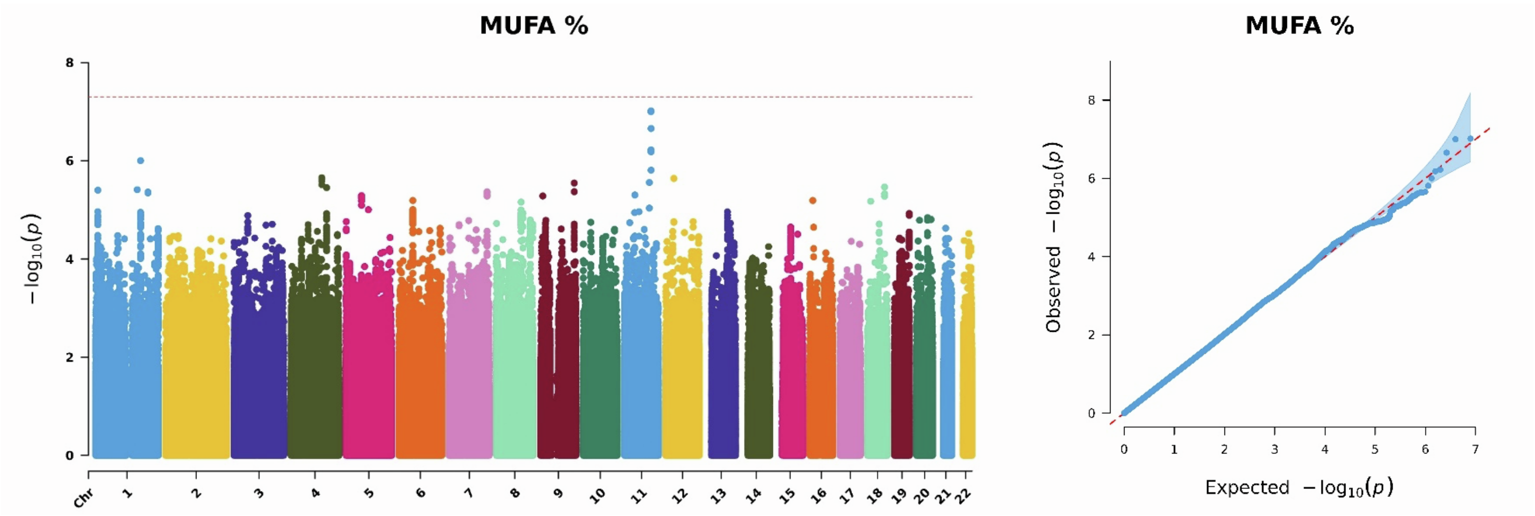
MUFA %
$-\log_{10}(p)$
Chr 1 2 3 4 5 6 7 8 9 10 11 12 13 14 15 16 17 18 19 20 21 22
MUFA %
Observed $-\log_{10}(p)$
Expected $-\log_{10}(p)$

J

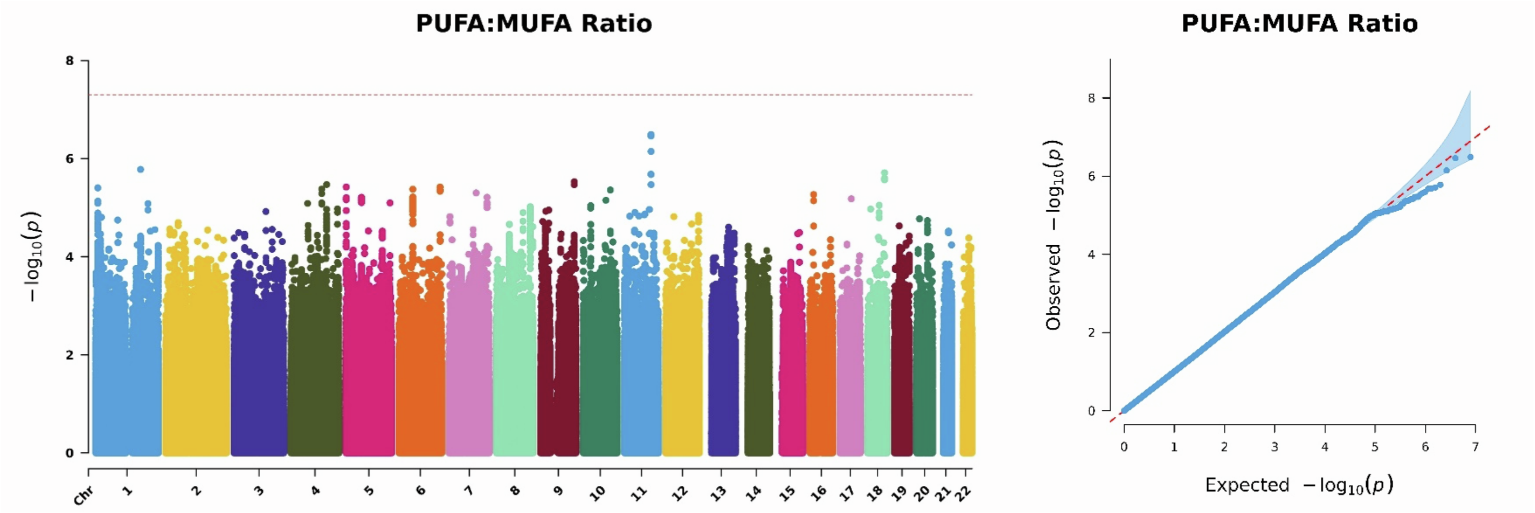
PUFA:MUFA Ratio
$-\log_{10}(p)$
Chr 1 2 3 4 5 6 7 8 9 10 11 12 13 14 15 16 17 18 19 20 21 22
PUFA:MUFA Ratio
Observed $-\log_{10}(p)$
Expected $-\log_{10}(p)$

K

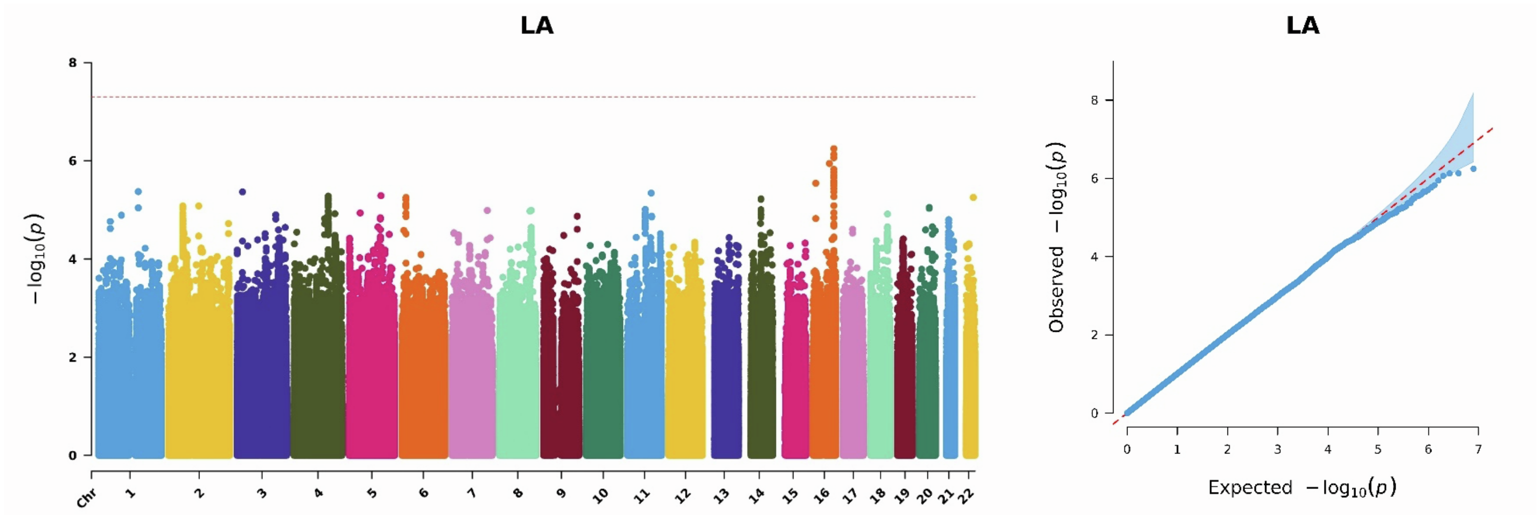

LA
LA
$-\log_{10}(p)$
Chr
1
2
3
4
5
6
7
8
9
10
11
12
13
14
15
16
17
18
19
20
21
22
Observed $-\log_{10}(p)$
Expected $-\log_{10}(p)$

L

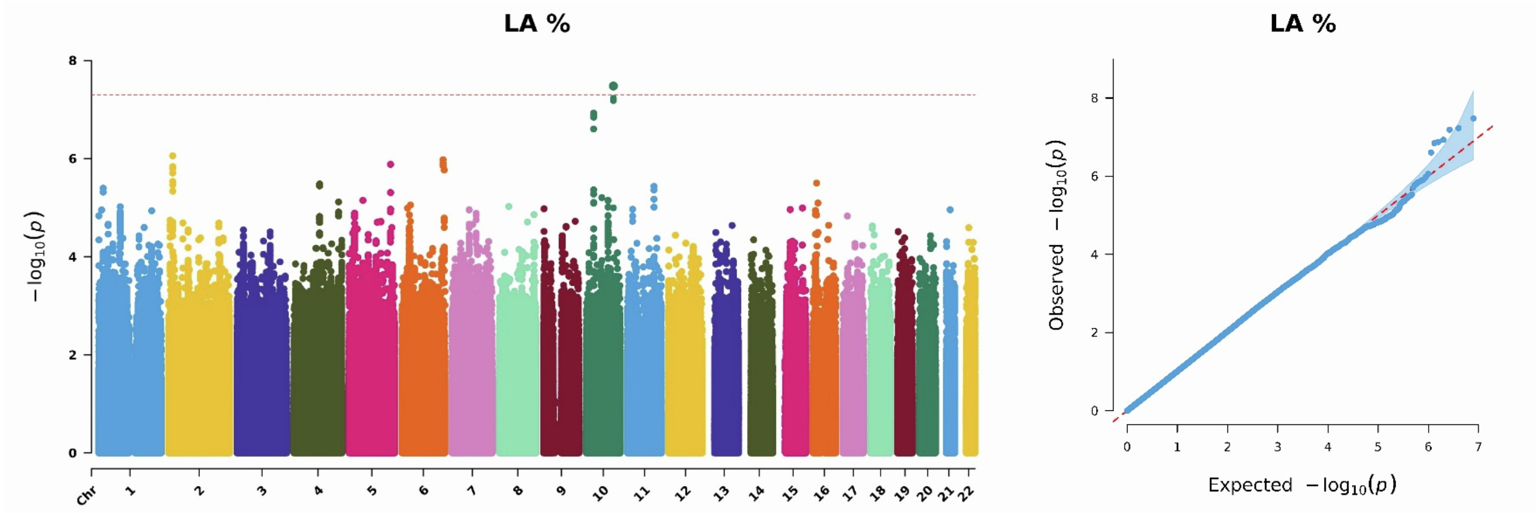

LA %
LA %
$-\log_{10}(p)$
Chr
1
2
3
4
5
6
7
8
9
10
11
12
13
14
15
16
17
18
19
20
21
22
Observed $-\log_{10}(p)$
Expected $-\log_{10}(p)$

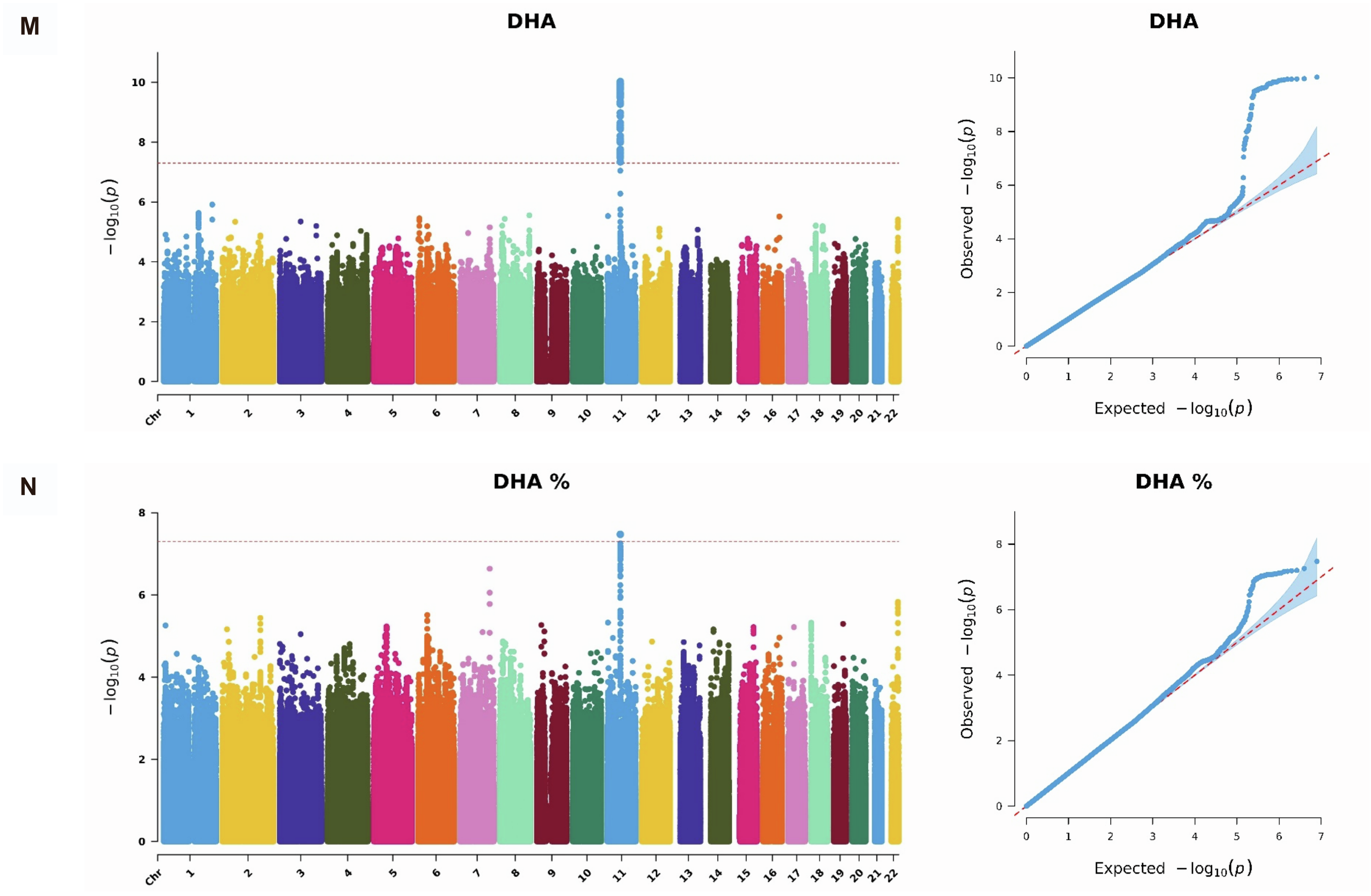

Figure S4. Manhattan and QQ plots of *p*-values for gene-fish oil interactions in 14 PUFAs and MUFAs-related phenotypes for 114,352 participants in the Phase Two dataset

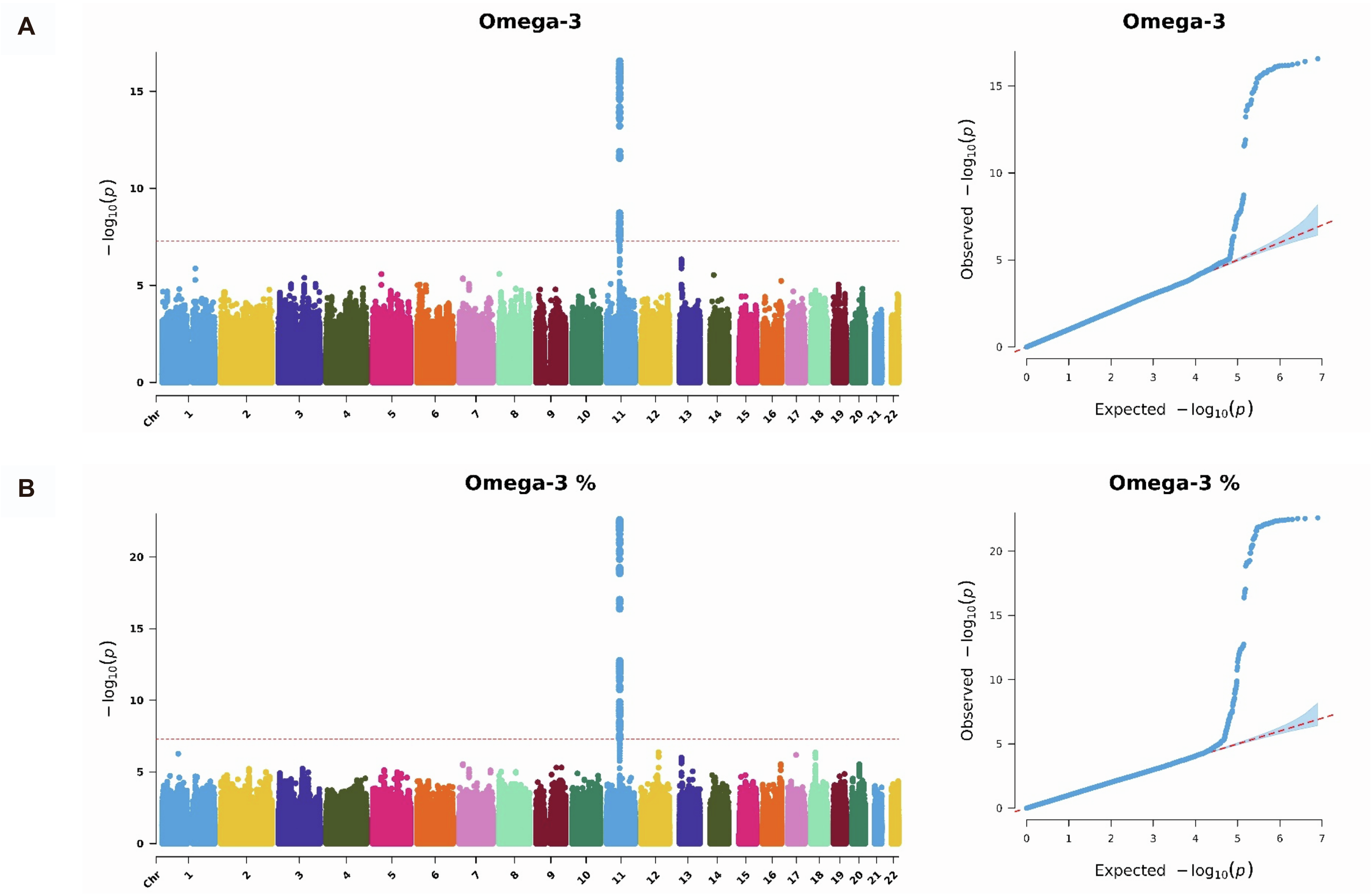
A
Omega-3
−log10(p)
Chr
Omega-3
Observed −log10(p)
Expected −log10(p)
B
Omega-3 %
−log10(p)
Chr
Omega-3 %
Observed −log10(p)
Expected −log10(p)

C

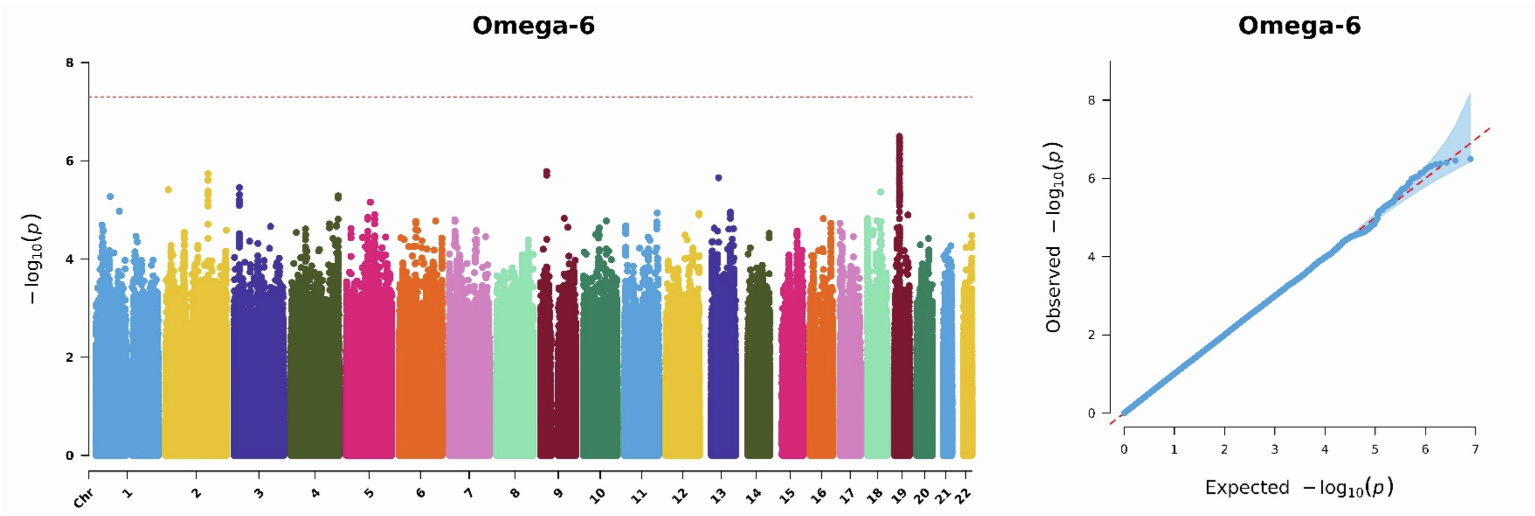
Omega-6
$-\log_{10}(p)$
Chr 1 2 3 4 5 6 7 8 9 10 11 12 13 14 15 16 17 18 19 20 21 22
Omega-6
Observed $-\log_{10}(p)$
Expected $-\log_{10}(p)$

D

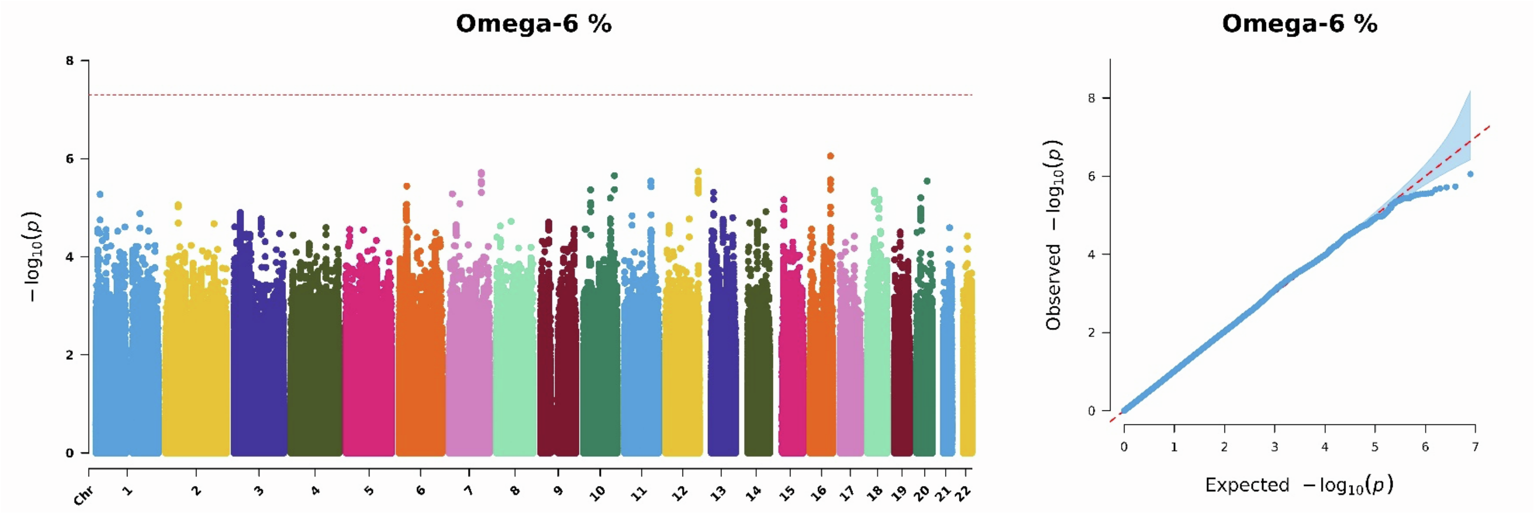
Omega-6 %
$-\log_{10}(p)$
Chr 1 2 3 4 5 6 7 8 9 10 11 12 13 14 15 16 17 18 19 20 21 22
Omega-6 %
Observed $-\log_{10}(p)$
Expected $-\log_{10}(p)$

E

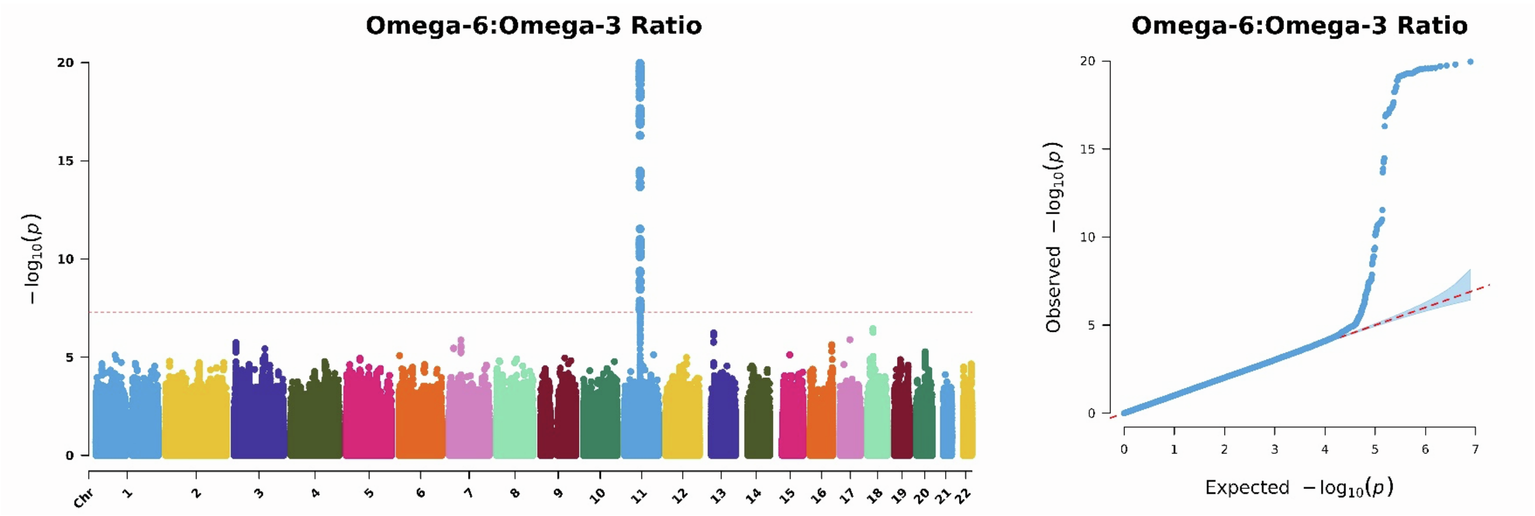
Omega-6:Omega-3 Ratio
$-\log_{10}(p)$
Chr
Omega-6:Omega-3 Ratio
Observed $-\log_{10}(p)$
Expected $-\log_{10}(p)$

F

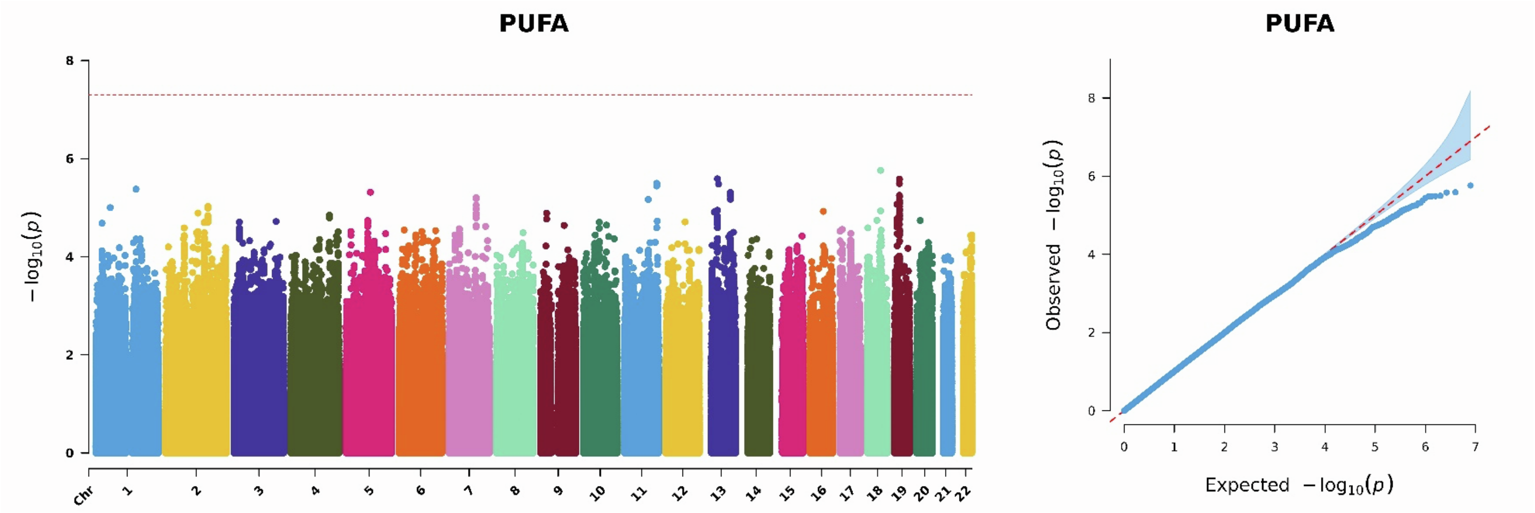
PUFA
$-\log_{10}(p)$
Chr
PUFA
Observed $-\log_{10}(p)$
Expected $-\log_{10}(p)$

G

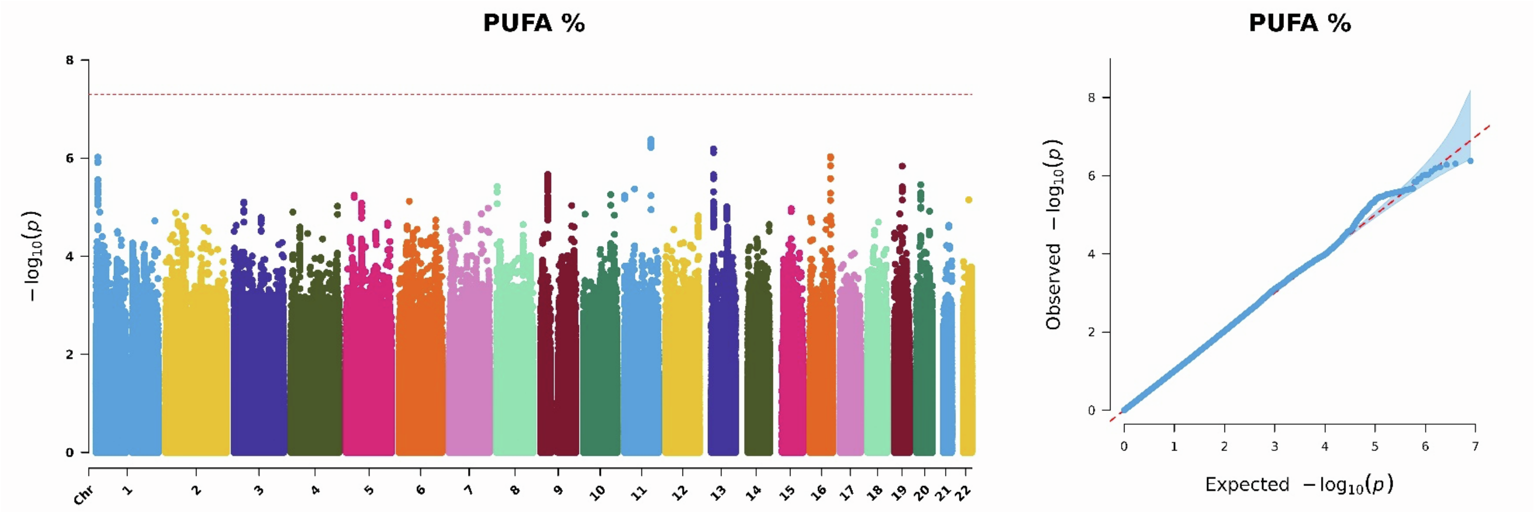
PUFA %
$-\log_{10}(p)$
Chr 1 2 3 4 5 6 7 8 9 10 11 12 13 14 15 16 17 18 19 20 21 22
PUFA %
Observed $-\log_{10}(p)$
Expected $-\log_{10}(p)$

H

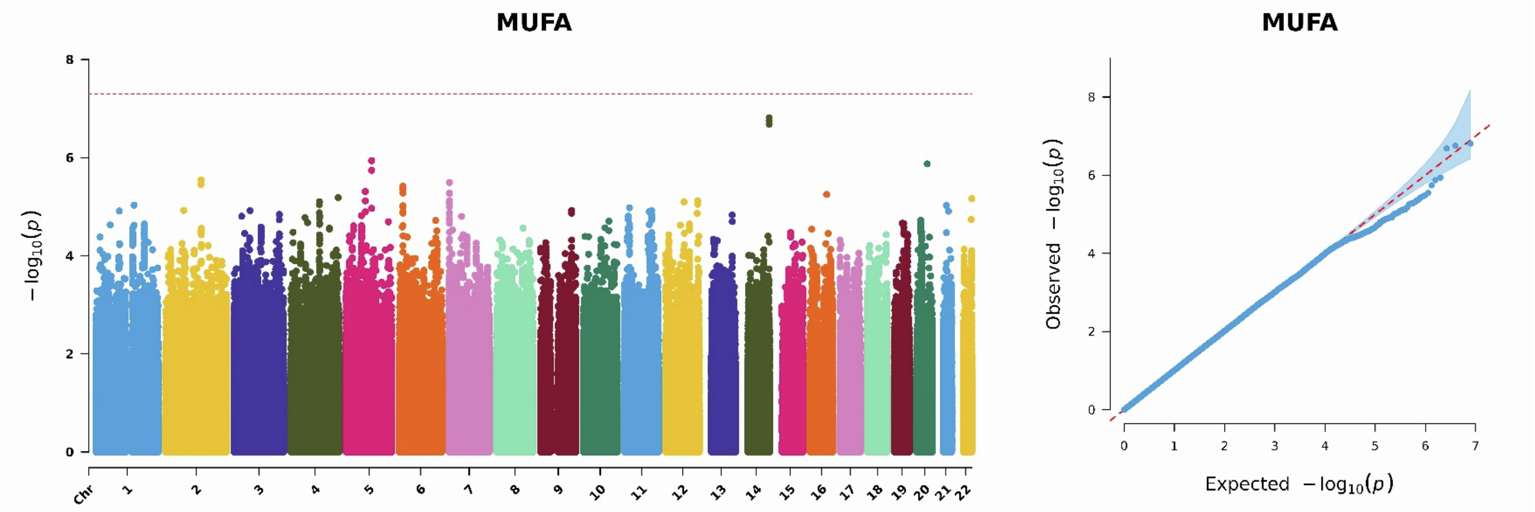
MUFA
$-\log_{10}(p)$
Chr 1 2 3 4 5 6 7 8 9 10 11 12 13 14 15 16 17 18 19 20 21 22
MUFA
Observed $-\log_{10}(p)$
Expected $-\log_{10}(p)$

I

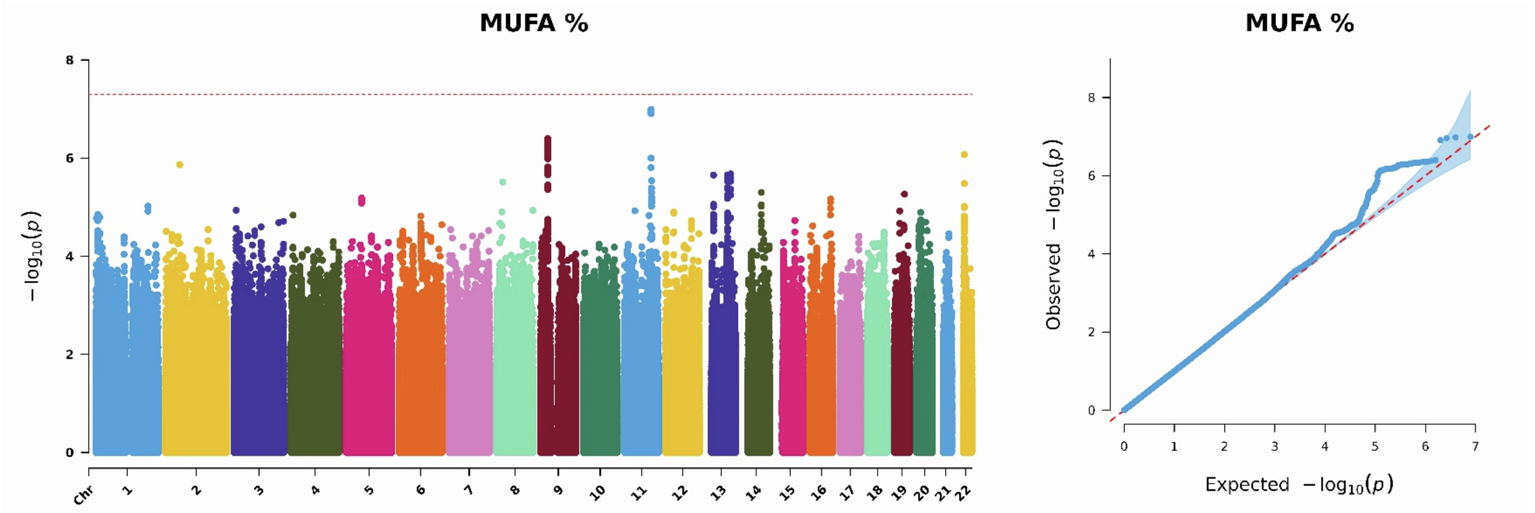

MUFA %
MUFA %
$-\log_{10}(p)$
Chr 1 2 3 4 5 6 7 8 9 10 11 12 13 14 15 16 17 18 19 20 21 22
Observed $-\log_{10}(p)$
Expected $-\log_{10}(p)$

J

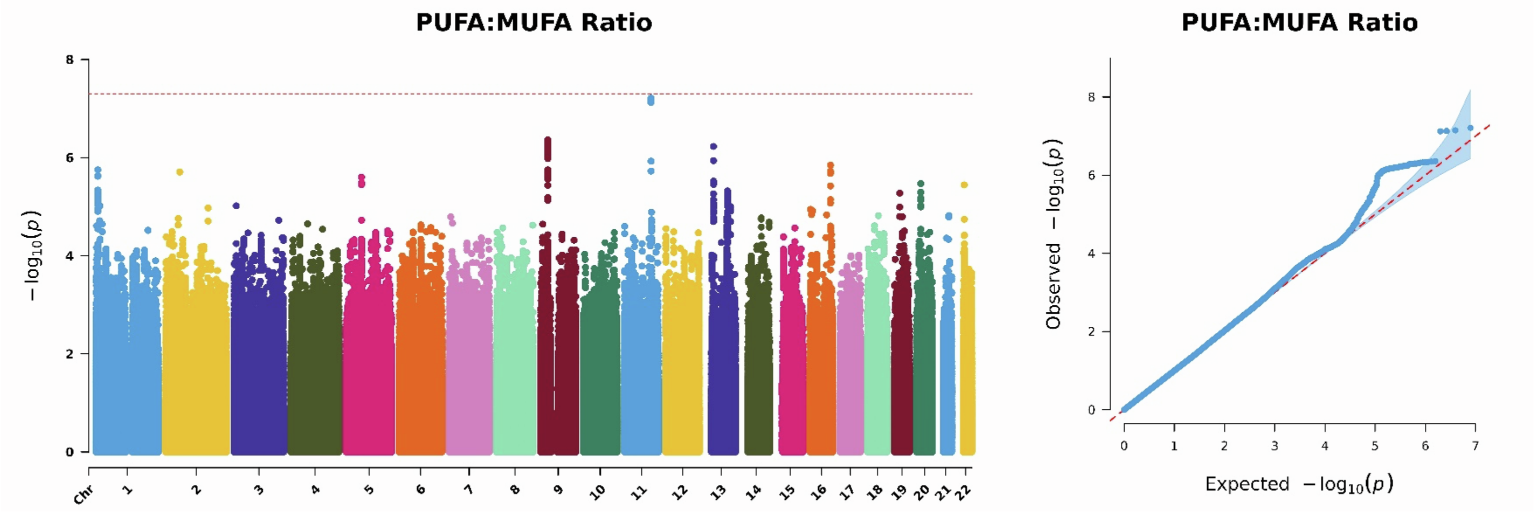

PUFA:MUFA Ratio
PUFA:MUFA Ratio
$-\log_{10}(p)$
Chr 1 2 3 4 5 6 7 8 9 10 11 12 13 14 15 16 17 18 19 20 21 22
Observed $-\log_{10}(p)$
Expected $-\log_{10}(p)$

K

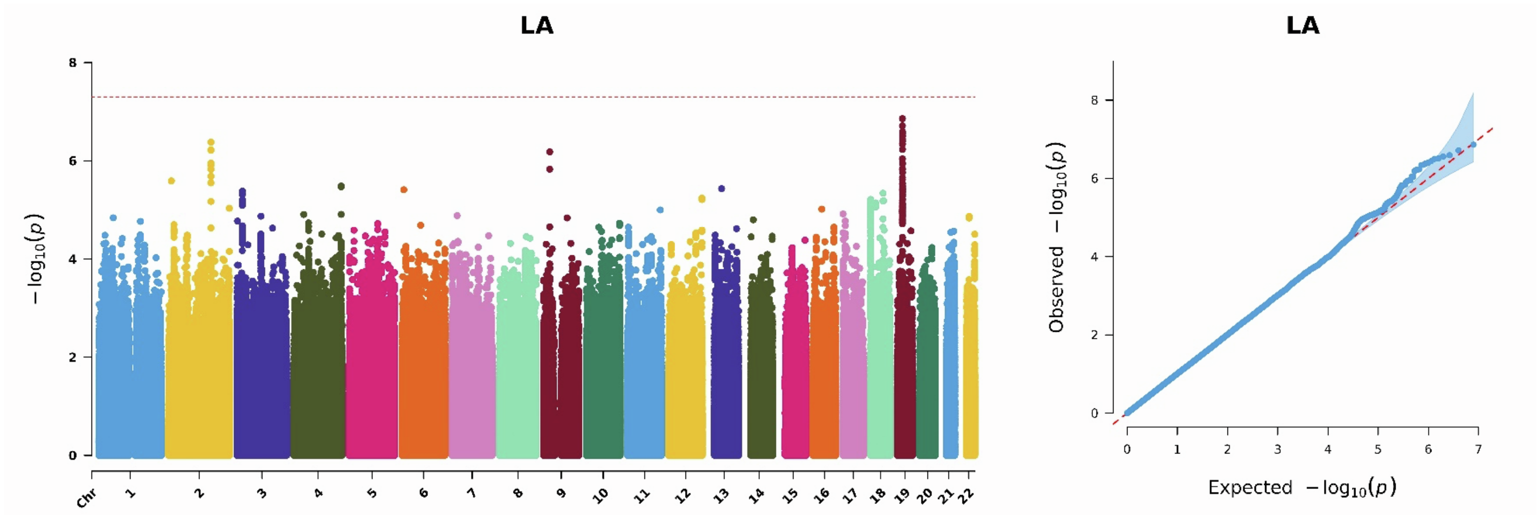
LA
LA
−log10(p)
Observed −log10(p)
Expected −log10(p)
Chr
1
2
3
4
5
6
7
8
9
10
11
12
13
14
15
16
17
18
19
20
21
22

L

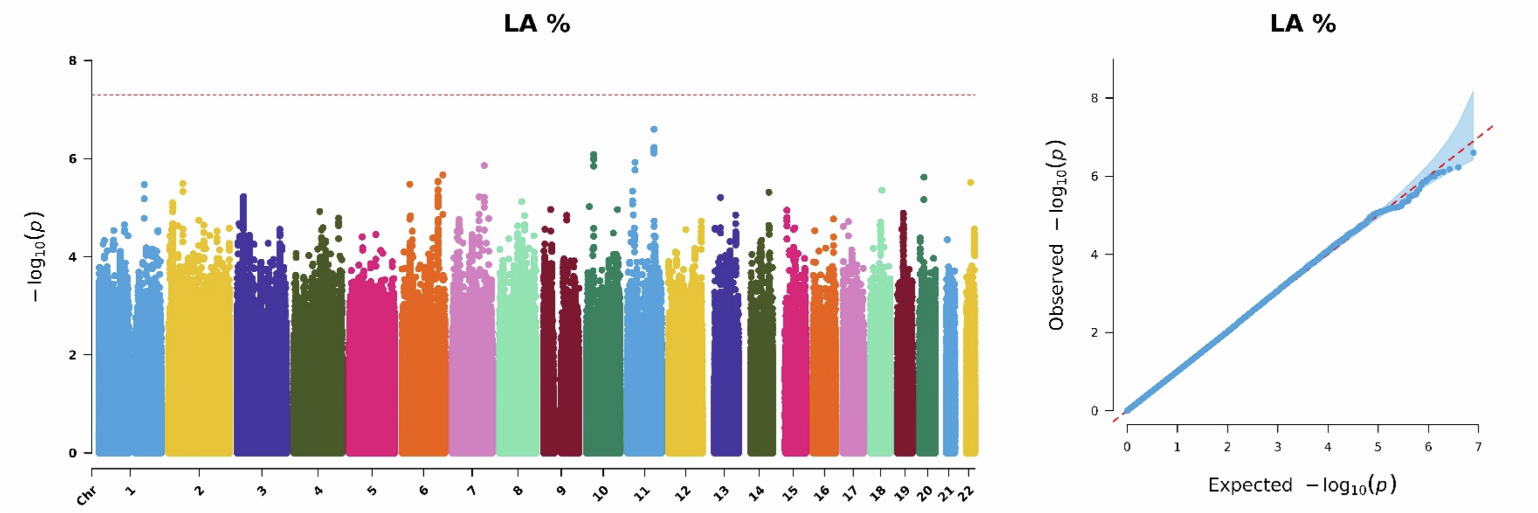
LA %
LA %
−log10(p)
Observed −log10(p)
Expected −log10(p)
Chr
1
2
3
4
5
6
7
8
9
10
11
12
13
14
15
16
17
18
19
20
21
22

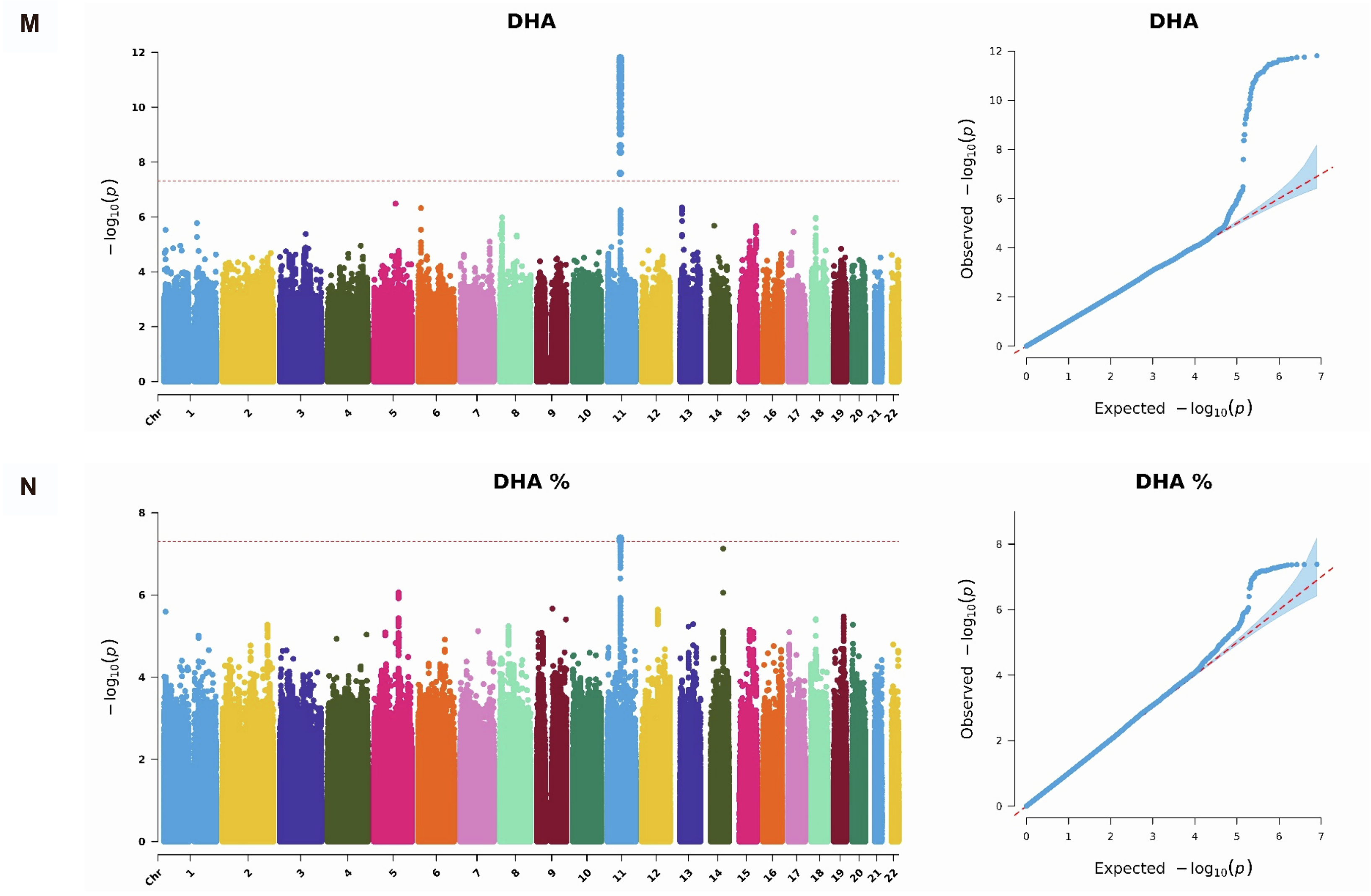

Figure S5. Manhattan and QQ plots of *p*-values for gene-fish oil interactions in 14 PUFAs and MUFAs-related phenotypes for 200,060 participants in the combined dataset

Table S1. Baseline characteristics of participants in Phase One, Phase Two, and combined releases of NMR metabolite data of individuals of European ancestry in UK Biobank

	Phase One (*n* = 85,708)	Phase Two (*n* = 114,352)	Combined phases (*n* = 200,060)
Age, years (SD)	57 (8.01)	57 (8.00)	57 (8.01)
Sex, female (%)	45,907 (54)	61,137 (54)	107,044 (54)
Fish oil intake, *n* (%)			
Yes	27,201 (32)	77,842 (32)	63,711 (32)
No	58,507 (68)	36,510 (68)	136,349 (68)
Absolute PUFA concentrations, mmoL/L (SD)			
Omega-3	0.52 (0.22)	0.54 (0.22)	0.53 (0.22)
Omega-6	4.44 (0.67)	4.56 (0.69)	4.51 (0.69)
DHA	0.23 (0.082)	0.24 (0.085)	0.24 (0.083)
LA	3.41 (0.67)	3.51 (0.69)	3.46 (0.69)
MUFA	2.83 (0.81)	2.98 (0.85)	2.92 (0.84)
PUFA	4.97 (0.79)	5.10 (0.82)	5.04 (0.81)
Proportion in total fatty acids, % (SD)			
Omega-3	4.39 (1.53)	4.35 (1.53)	4.37 (1.53)
Omega-6	37.97 (3.59)	37.57 (3.58)	37.74 (3.59)
DHA	2.00 (0.66)	1.98 (0.67)	1.99 (0.67)
LA	28.94 (3.40)	28.71 (3.38)	28.81 (3.39)
MUFA	23.55 (2.67)	23.97 (2.65)	23.79 (2.67)
PUFA	42.37 (3.73)	41.92 (3.72)	42.11 (3.73)
Ratios (SD)			
Omega-6 to Omega-3	9.82 (4.28)	9.81 (4.23)	9.81 (4.25)
PUFA to MUFA	1.84 (0.35)	1.78 (0.33)	1.81 (0.34)
